# Repercussion of a 1,3-Hydrogen Shift in a Hydride-Osmium-Allenylidene Complex

**DOI:** 10.1021/acs.organomet.1c00176

**Published:** 2021-05-12

**Authors:** Miguel A. Esteruelas, Enrique Oñate, Sonia Paz, Andrea Vélez

**Affiliations:** Departamento de Química Inorgánica − Instituto de Síntesis Química y Catálisis Homogénea (ISQCH) − Centro de Innovación en Química Avanzada (ORFEO−CINCA), Universidad de Zaragoza − CSIC, 50009 Zaragoza, Spain

## Abstract

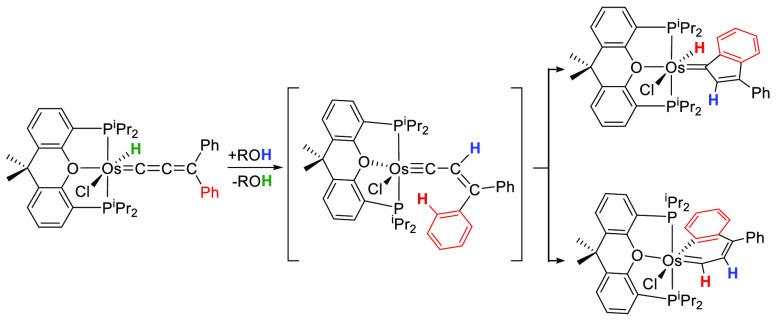

An unusual 1,3-hydrogen shift from the metal center to the C_β_ atom of the C_3_-chain of the allenylidene ligand in a hydride-osmium(II)-allenylidene complex is the beginning of several interesting transformations in the cumulene. The hydride-osmium(II)-allenylidene complex was prepared in two steps, starting from the tetrahydride dimer [(Os(H···H){κ^3^-*P*,*O*,*P*-[xant(P^*i*^Pr_2_)_2_]})_2_(μ-Cl)_2_][BF_4_]_2_ (**1**). Complex **1** reacts with 1,1-diphenyl-2-propyn-1-ol to give the hydride-osmium(II)-alkenylcarbyne [OsHCl(≡CCH=CPh_2_){κ^3^-*P*,*O*,*P*-[xant(P^*i*^Pr_2_)_2_]}]BF_4_ (**2**), which yields OsHCl(=C=C=CPh_2_){κ^3^-*P*,*O*,*P*-[xant(P^*i*^Pr_2_)_2_]} (**3**) by selective abstraction of the C_β_–H hydrogen atom of the alkenylcarbyne ligand with K^*t*^BuO. Complex **3** is metastable. According to results of DFT calculations, the migration of the hydride ligand to the C_β_ atom of the cumulene has an activation energy too high to occur in a concerted manner. However, the migration can be catalyzed by water, alcohols, and aldehydes. The resulting alkenylcarbyne-osmium(0) intermediate is unstable and evolves into a 7:3 mixture of the hydride-osmium(II)-indenylidene OsHCl(=C_IndPh_){κ^3^-*P*,*O*,*P*-[xant(P^*i*^Pr_2_)_2_]} (**4**) and the osmanaphthalene OsCl(C_9_H_6_Ph){κ^3^-*P*,*O*,*P*-[xant(P^*i*^Pr_2_)_2_]} (**5**). Protonation of **4** with HBF_4_ leads to the elongated dihydrogen complex [OsCl(η^2^-H_2_)(=C_IndPh_){κ^3^-*P*,*O*,*P*-[xant(P^*i*^Pr_2_)_2_]}]BF_4_ (**6**), while the protonation of **5** regenerates **2**. In contrast to **4**, complex **6** evolves to a half-sandwich indenyl derivative, [Os(η^5^-IndPh)H{κ^3^-*P*,*O*,*P*-[xant(P^*i*^Pr_2_)_2_]}][BF_4_]Cl (**7**). Phenylacetylene also provokes the 1,3-hydrogen shift in **3**. However, it does not participate in the migration. In contrast to water, alcohols, and aldehydes, it stabilizes the resulting alkenylcarbyne to afford [Os(≡CCH=CPh_2_)(η^2^-HC≡CPh){κ^3^-*P*,*O*,*P*-[xant(P^*i*^Pr_2_)_2_]}]Cl (**8**).

## Introduction

Transition metal unsaturated carbene complexes, particularly vinylidene and allenylidene derivatives, are modern and powerful tools in organic and organometallic synthesis. Their use is allowing the development of previously inaccessible or difficult transformations, which simplifies the building of a diverse range of types of carbon–carbon and carbon–heteroatom bonds.^1^ Other tools of paramount relevance are the transition metal hydride complexes. They are classical inorganic compounds,^2^ which are increasingly used in homogeneous catalysis.^3^ The reason for this fact is because they are ideal for setting unsaturated organic molecules at metal fragments,^4^ can generate radicals with Markovnikov selectivity by H· transfer,^5^ and have demonstrated a marked ability to functionalize C–H bonds as consequence of their capacity to activate σ-bonds.^6^ Thus, complexes bearing both classes of ligands have an enormous potentiality, being the stabilization and control over their chemical properties a challenge of first magnitude.

The main problem for the development of the stoichiometric chemistry of these bifunctional compounds, which enables to understand the catalytic performance, is their low stability, since they are thermodynamically unstable with regard to the 1,2-insertion products (eq 1).^7^ As a consequence, only a scarce number of hydride-vinylidene complexes of 8 group metals have been isolated and fully characterized so far,^8^ mainly osmium derivatives,^8a−8e,8g−8j^ whereas the known hydride-allenylidene compounds are reduced to the cations [OsH(=C=C=CPh_2_)(CH_3_CN)_2_(P^*i*^Pr_3_)_2_]^+^ and [OsH(=C=C=CPh_2_)(η^2^-HC≡CH)(P^*i*^Pr_3_)_2_]^+^^9^ and the neutral iridium(III) complexes IrHCl(=C=C=CPhR)(P^*i*^Pr_3_)_2_ (R = Ph, ^*t*^Bu),^10^ although only some reactivity of the first of them has been investigated.^11^
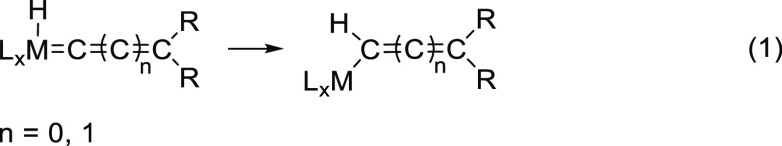
1

**Scheme 1:**
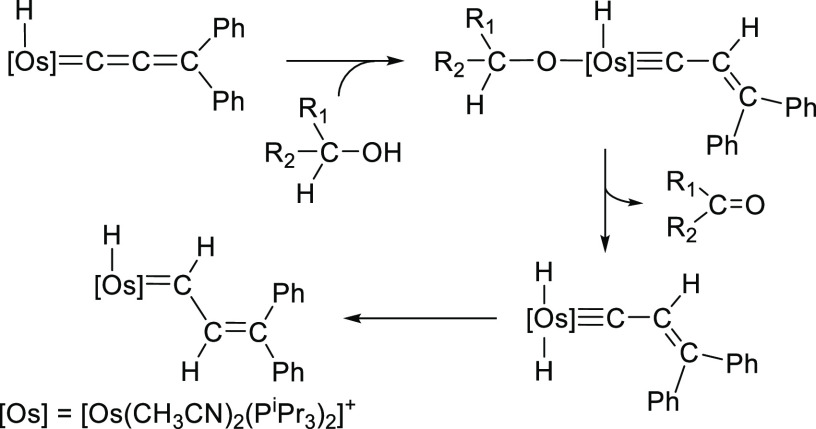
Reduction of [OsH(=C=C=CPh_2_)(CH_3_CN)_2_(P^*i*^Pr_3_)_2_]^+^ with Secondary Alcohols

Transition metal allenylidene complexes can be grouped into electrophiles and nucleophiles, according to the reactivity of the unsaturated C_3_-chain. While electrophiles have attracted great attention, nucleophiles have been scarcely studied. The nucleophilic allenylidenes are characterized by addition of electrophiles to C_β_. With alcohols, the great majority of them are inert.^1b^ However, the allenylidene ligand of cation [OsH(=C=C=CPh_2_)(CH_3_CN)_2_(P^*i*^Pr_3_)_2_]^+^ displays supernucleophilic behavior, which allows the reduction of the C_α_–C_β_ double bond of the unsaturated chain (Scheme 1). The 1,3-addition of the O–H alcohol bond to the metal center and the C_β_ atom affords an alkoxide-hydride-carbyne intermediate, which leads to dihydride-carbyne species by β-hydrogen elimination of the alcoholate. The subsequent migration of one of the hydrides to the carbyne C_α_ atom gives the reduction product hydride-alkenylcarbene.^11a,11d^

**Scheme 2:**
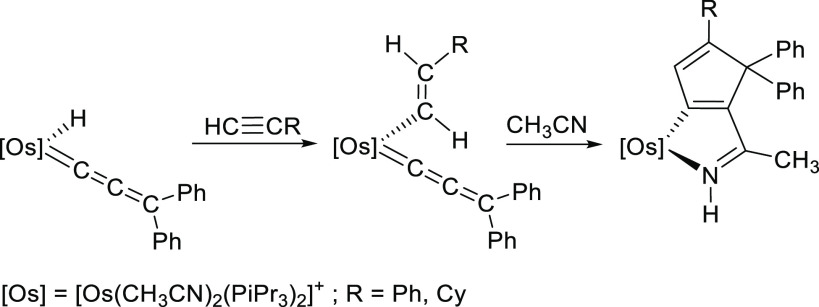
Formation of Metalacyclopentapyrrole Derivatives

The hydride ligand of [OsH(=C=C=CPh_2_)(CH_3_CN)_2_(P^*i*^Pr_3_)_2_]^+^ is certainly efficient for fixing unsaturated organic molecules beside the allenylidene ligand. It reacts with terminal alkynes to afford alkenyl-osmium(II)-allenylidene derivatives, which evolve into metalacyclopentapyrrole compounds in acetonitrile (Scheme 2). Their formation implies the genesis of three carbon–carbon bonds. The C_α_ and C_γ_ atoms of the cumulene are coupled with the C_α_ and C_β_ atoms of the alkenyl group, whereas the C_β_ atom of the C_3_-chain is attacked by the electrophilic C(sp) atom of the solvent.^9^

The mentioned reactions of [OsH(=C=C=CPh_2_)(CH_3_CN)_2_(P^*i*^Pr_3_)_2_]^+^ evoked us a question: what is the driving force of this unusual behavior, the charge of the complex, the weak coordinating ability of the acetonitrile ligand, or both? During some years, we unsuccessfully looked for a metal fragment that would allow us to address this question. In 2010, we reported the preparation of the ether-diphosphine 9,9-dimethyl-4,5-bis(diisopropylphosphino)xanthene (xant(P^*i*^Pr_2_)_2_), which can keep the *trans*-P–Os–P arrangement observed in the cation, by means of its coordination κ^3^-*P*,*O*,*P*-*mer*.^12^ In addition, Weller’s group has demonstrated in the past years that POP-diphosphines are flexible hemilabile ligands.^13^ According to such ability, xant(P^*i*^Pr_2_)_2_ is adapted to the requirements of the reactions in which its complexes participate, enabling the necessary geometrical transformations on the metal coordination sphere through changes in its coordination mode.^14^ As proof-of-concept validation, besides species bearing the diphosphine κ^3^-*P*,*O*,*P*-*mer* coordinated,^15^ complexes with the ligand in fashions κ^3^-*P*,*O*,*P*-*fac*,^16^ κ^2^-*P*,*P*-*cis*,^17^ and κ^2^-*P*,*P*-*trans*^14b,18^ have been also isolated. This is allowing to perform reactions^19^ and to isolate compounds^20^ initially forbidden, and as result, interesting catalysts for a wide range of processes are being discovered.^6b,6c,19,21^ The proved flexibility and great coordinative versatility of xant(P^*i*^Pr_2_)_2_ inspired us to use it to address the preparation of a neutral hydride-osmium(II)-allenylidene complex. Its stabilization would permit to study its behavior toward alcohols, water, and terminal alkynes and to in this way answer the question above.

This paper reports the preparation of a neutral hydride-osmium(II)-allenylidene complex structurally related to the cation [OsH(=C=C=CPh_2_)(CH_3_CN)_2_(P^*i*^Pr_3_)_2_]^+^ and, in order to address the question raised, analyzes its behavior toward alcohols, water, aldehydes, and phenylacetylene, which promote an unusual 1,3-hydrogen shift from the metal center to the C_β_ atom of the cumulene.

## Results and Discussion

### Preparation of the Neutral Hydride-Osmium(II)-Allenylidene Complex

**Scheme 3:**
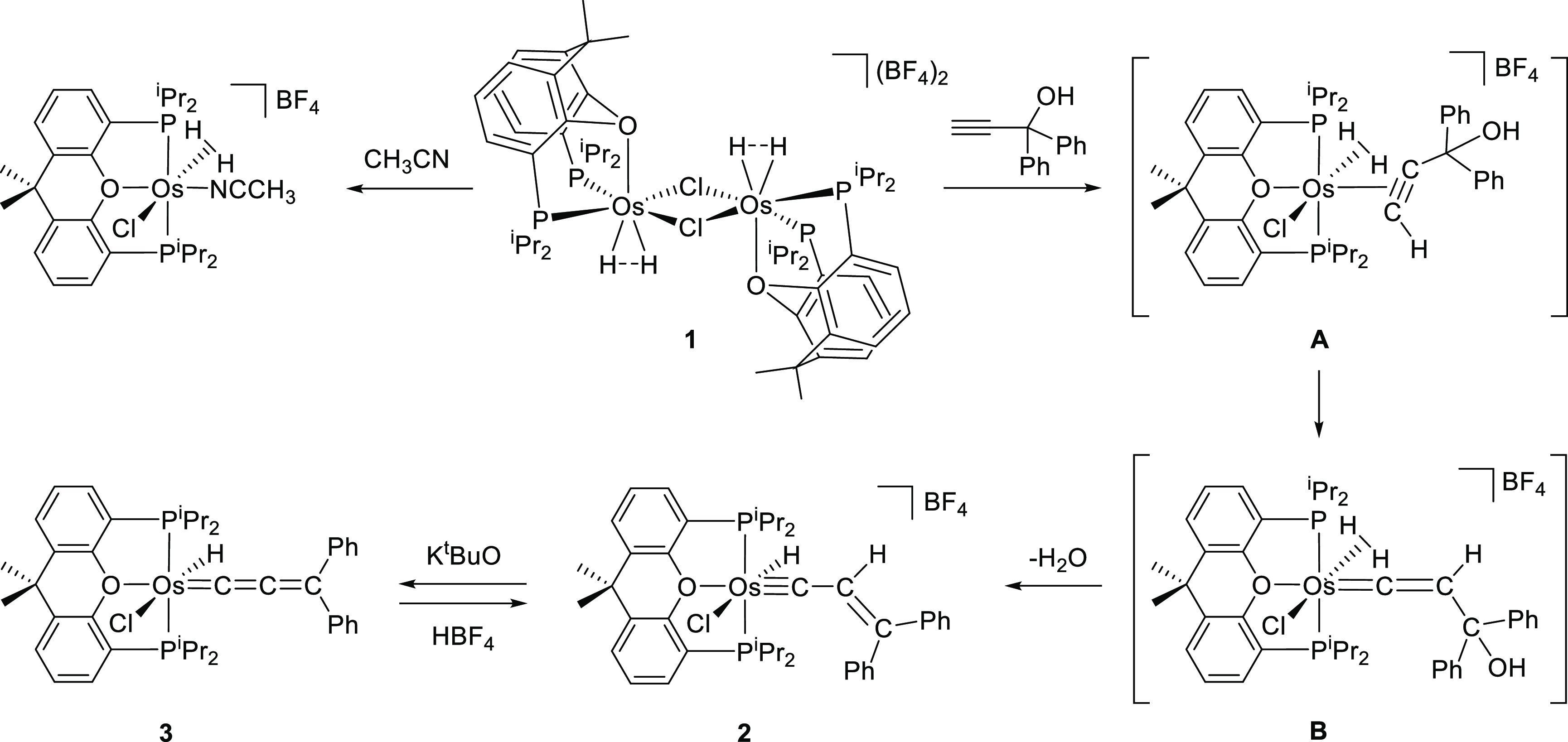
Synthesis of OsHCl(=C=C=CPh_2_){κ^3^-*P*,*O*,*P*-[xant(P^*i*^Pr_2_)_2_]}

Scheme 3 summarizes the strategy employed to obtain the target compound. We selected the tetrahydride dimer [(Os(H···H){κ^3^-*P*,*O*,*P*-[xant(P^*i*^Pr_2_)_2_]})_2_(μ-Cl)_2_][BF_4_]_2_ (**1**) as the starting point, despite the κ^3^-*P*,*O*,*P*-*fac* coordination of the ether-diphosphine, because it reacts with weak Lewis bases to give mononuclear six-coordinate elongated dihydrogen derivatives, displaying a tridentate ligand κ^3^-*P*,*O*,*P*-*mer* coordinated. For instance, acetonitrile yields [OsCl(η^2^-H_2_)(CH_3_CN){κ^3^-*P*,*O*,*P*-[xant(P^*i*^Pr_2_)_2_]}]BF_4_. The *fac*-disposition of the diphosphine in **1** stabilizes the dimeric structure with regard to the *mer*-coordination due to a decrease of the steric hindrance experienced by the isopropyl substituents of the unsaturated fragments. However, the coordination *mer* is favored over *fac* for mononuclear saturated metal centers. Acetonitrile breaks the chloride bridges of the dimer, saturating the osmium center. At a time, the compressed dihydrides are approached to form an elongated dihydrogen, whereas the ether-diphosphine changes its disposition from *fac* to *mer*, as a consequence of the disappearance of the steric hindrance.^16^ In this context, it should be mentioned that unsaturated osmium-dihydride complexes, which afford dihydrogen species by coordination of electron poor Lewis bases, react with propargyl alcohols to give hydride-osmium-alkenylcarbyne derivatives. The π-C≡C coordination of the alkynol at the vacancy promotes its tautomerization to hydroxyvinylidene, which undergoes dehydration and addition of the acidic atom of the generated dihydrogen.^11d^ According to this, 1,1-diphenyl-2-propyn-1-ol reacts with the tetrahydride dimer, in fluorobenzene at 80 °C, to form the expected hydride-osmium-alkenylcarbyne [OsHCl(≡CCH=CPh_2_){κ^3^-*P*,*O*,*P*-[xant(P^*i*^Pr_2_)_2_]}]BF_4_ (**2**), through intermediates **A** and **B**. Complex **2** was isolated as a red solid in 76% yield. Despite the expected acidity of the hydride ligand of **2**, the treatment of its tetrahydrofuran solutions with 1.1 equiv of K^*t*^BuO produces the selective abstraction of the C_β_–H hydrogen atom of the alkenylcarbyne ligand. The deprotonation affords the desired allenylidene ligand. Complex OsHCl(=C=C=CPh_2_){κ^3^-*P*,*O*,*P*-[xant(P^*i*^Pr_2_)_2_]} (**3**) was isolated as a green solid in 86% yield. The deprotonation is reversible; the addition of 1.0 equiv of HBF_4_ to dichloromethane solutions of **3** quantitatively regenerates **2**.

**Figure 1:**
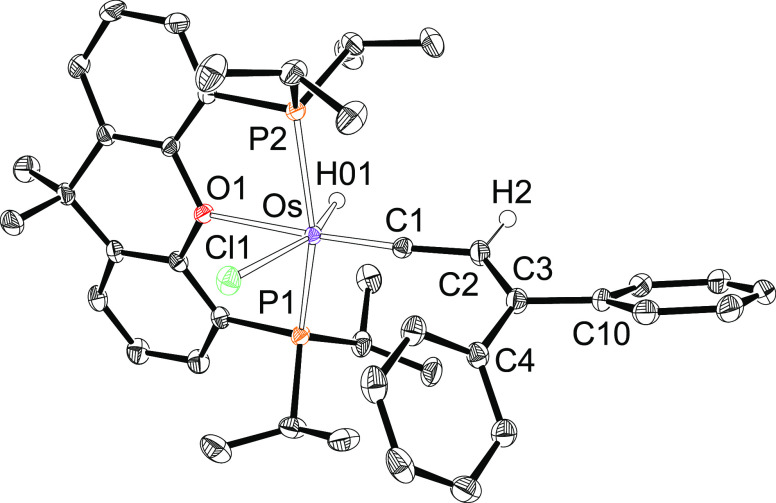
Molecular diagram of the cation of complex **2** (ellipsoids shown at 50% probability). All hydrogen atoms (except C_β_–H and the hydride) are omitted for clarity. Selected bond distances (Å) and angles (deg): Os–P(1) = 2.3487(6), Os–P(2) = 2.3601(6), Os–O(1) = 2.3150(15), Os–Cl(1) = 2.4403(6), Os–H(01) = 1.595(9), Os–C(1) = 1.731(2), C(1)–C(2) = 1.423(3), C(2)–C(3) = 1.360(3); P(1)–Os–P(2) = 155.95(2), C(1)–Os–O(1) = 173.09(8), C(1)–C(2)–C(3) = 127.8(2), C(2)–C(3)–C(4) = 124.0(2), C(2)–C(3)–C(10) = 119.3(2), P(1)–Os–O(1) = 79.32(4), P(2)–Os–O(1) = 79.57(4), H(01)–Os–Cl(1) = 158.8(9).

Complex **2** was characterized by X-ray diffraction analysis. Figure 1 shows a view of the cation. The structure supports the formation of the alkenylcarbyne ligand and the *mer*-disposition of the diphosphine, which coordinates with P(1)–Os–P(2), P(1)–Os–O(1), and P(2)–Os–O(1) angles of 155.95(2)°, 79.32(4)°, and 79.57(4)°, respectively. Thus, the coordination polyhedron around the osmium atom can be rationalized as a distorted octahedron with the carbyne group disposed *trans* to the oxygen atom of the diphosphine (C(1)–Os–O(1) = 173.09(8)°) and the hydride ligand situated *trans* to the chloride anion (H(01)–Os–Cl(1) = 158.8(9)°). The most conspicuous feature of the structure is the very short Os–C(1) bond length of 1.731(2) Å, which is fully consistent with an Os–C(1) triple bond formulation.^22^ The alkenylcarbyne proposal is supported by the bond lengths and angles within the carbon donor ligand. Carbons C(1) and C(2) are separated by 1.423(3) Å, whereas the C(2)–C(3) distance is 1.360(3) Å. The angles around C(2) and C(3) lie in the range 112–127°. In agreement with the presence of a hydride ligand, the ^1^H NMR spectrum, in dichloromethane-*d*_2_, at room temperature shows a triplet (^2^*J*_H–P_ = 16.4 Hz) at −5.59 ppm. In the low field region of the spectrum, the most noticeable signal is a singlet at 5.55 ppm corresponding to the C(sp^2^)–H hydrogen atom of the alkenyl group. In the ^13^C{^1^H} spectrum the Os–C(sp) resonance appears at 271.5 ppm, as a triplet with a C–P coupling constant of 5.6 Hz, whereas the alkenylcarbyne C(sp^2^) resonances are observed at 166.9 and 130.8 ppm as singlets. The ^31^P{^1^H} NMR spectrum contains a singlet at 52.9 ppm, as expected for equivalent P^*i*^Pr_2_ groups.

**Figure 2:**
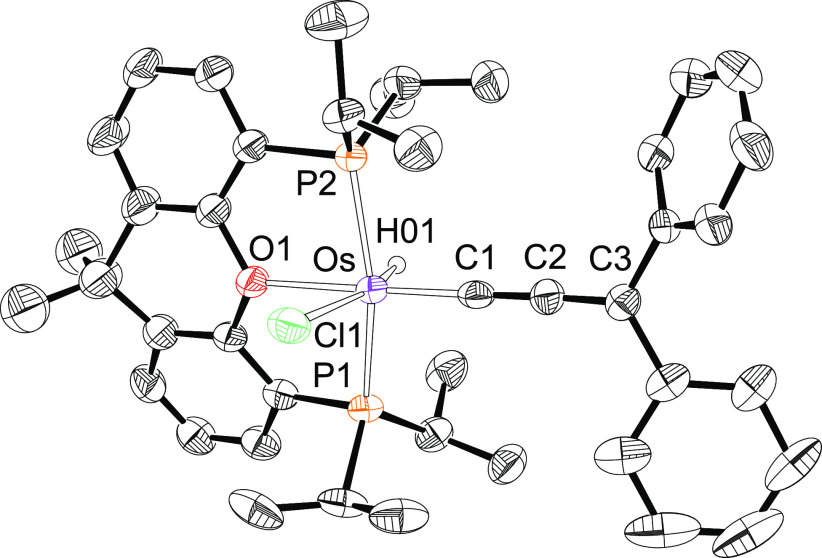
Molecular diagram of complex **3** (ellipsoids shown at 50% probability). All hydrogen atoms (except the hydride) are omitted for clarity. Selected bond distances (Å) and angles (deg): Os–P(1) = 2.3200(15), Os–P(2) = 2.3072(15), Os–O(1) = 2.239(4), Os–Cl(1) = 2.4846(16), Os–H(01) = 1.580(10), Os–C(1) = 1.858(6), C(1)–C(2) = 1.261(8), C(2)–C(3) = 1.351(8); P(1)–Os–P(2) = 160.88(6), C(1)–Os–O(1) = 177.8(2), H(01)–Os–Cl(1) = 164(2), Os–C(1)–C(2) = 175.1(5), C(1)–C(2)–C(3) = 175.1(6).

Complex **3** has been also characterized by X-ray diffraction analysis. Figure 2 shows a view of the molecule. The coordination around the osmium atom resembles that of **2**, with the allenylidene ligand in the position of the alkenylcarbyne group; i.e., a distorted octahedral arrangement with P(1)–Os–P(2), C(1)–Os–O(1), and H(01)–Os–Cl(1) angles of 160.88(6)°, 177.8(2)°, and 164(2)°, respectively. The diphenylallenylidene ligand is bonded to the metal center in a nearly linear fashion (Os–C(1)–C(2) = 175.1(5)° and C(1)–C(2)–C(3) = 175.1(6)°). The Os–C(1), C(1)–C(2), and C(2)–C(3) distances of 1.858(6), 1.261(8), and 1.351(8) Å, respectively, compare well with those reported for the previously structurally characterized osmium-allenylidene complexes.^9,21b,23^ In agreement with them, C(1)–C(2) and C(2)–C(3) are about 0.05 Å shorter and longer, respectively, than the bond length expected for a carbon–carbon double bond (about 1.30 Å), which suggests a notable contribution of the canonical form [M]^−^—C≡C—C^+^Ph_2_ to the structure of the C_3_-chain. In accordance with the presence of hydride and allenylidene ligands, the IR spectrum of the molecule contains the corresponding characteristic ν(Os–H) and ν(C=C=C) bands at 2090 and 1863 cm^–1^. In the ^1^H NMR spectrum, in dichloromethane-*d*_2_, at room temperature, the hydride resonance appears as a triplet (^2^*J*_H–P_ = 17.4 Hz) at −8.82 ppm. In the ^13^C{^1^H} NMR spectrum, the C_3_-chain gives rise to three triplets at 154.8, 242.5, and 256.1 ppm, with C–P coupling constants of 2.4, 10.4, and 4.1 Hz, which were assigned to the C_γ_, C_α_, and C_β_ atoms, respectively. The ^31^P{^1^H} spectrum displays a singlet at 27.6 ppm.

### Isomerization of **3** Promoted by Water, Alcohols, and Aldehydes

**Scheme 4:**
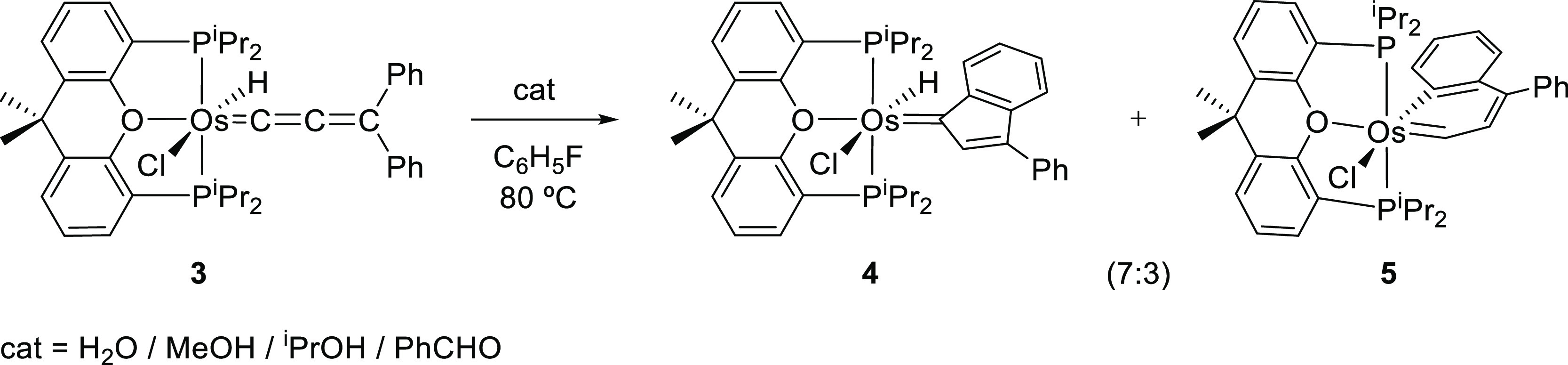
Isomerization of OsHCl(=C=C=CPh_2_){κ^3^-*P*,*O*,*P*-[xant(P^*i*^Pr_2_)_2_]}

Treatment of **3** with 1–2 equiv of water, methanol, 2-propanol, or benzaldehyde, in fluorobenzene, at 80 °C, for 16 h gives rise to its quantitative isomerization into a 7:3 mixture of the derivatives hydride-indenylidene OsHCl(=C_IndPh_){κ^3^-*P*,*O*,*P*-[xant(P^*i*^Pr_2_)_2_]} (**4**) and osmanaphthalene OsCl(C_9_H_6_Ph){κ^3^-*P*,*O*,*P*-[xant(P^*i*^Pr_2_)_2_]} (**5**) (Scheme 4), which were separated by using their different solubility in methanol and isolated as red and green crystals, respectively.

**Figure 3:**
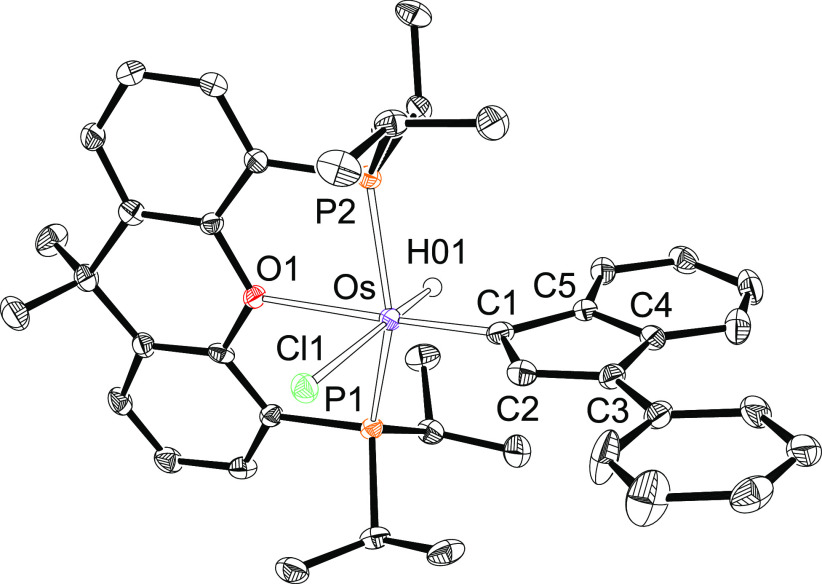
Molecular diagram of complex **4** (ellipsoids shown at 50% probability). All hydrogen atoms (except the hydride) are omitted for clarity. Selected bond distances (Å) and angles (deg): Os–P(1) = 2.3252(9), Os–P(2) = 2.2952(9), Os–O(1) = 2.377(2), Os–Cl(1) = 2.5254(8), Os–H(01) = 1.573(10), Os–C(1) = 1.901(3), C(1)–C(2) = 1.457(5), C(2)–C(3) = 1.360(5), C(3)–C(4) = 1.495(5), C(4)–C(5) = 1.412(4), C(5)–C(1) = 1.492(5); P(1)–Os–P(2) = 148.66(3), C(1)–Os–O(1) = 175.26(11), P(1)–Os–O(1) = 77.63(6), P(2)–Os–O(1) = 77.53(6), H(01)–Os–Cl(1) = 172.7(12).

Complex **4** is a notable example of stable hydride-indenylidene, which does not evolve to the half-sandwich indenyl species. Figure 3 shows its structure which proves the cyclization of the cumulene of **3** and the mutual disposition *cis* of the hydride ligand and the C(sp^2^) atom of the carbocycle. The coordination polyhedron around the osmium atom is the expected octahedron for a six-coordinate *d*^6^-ion with the ether-diphosphine κ^3^-*P*,*O*,*P*-*mer* coordinated (P(1)–Os–P(2) = 148.66(3)°, P(1)–Os–O(1) = 77.63(6)°, and P(2)–Os–O(1) = 77.53(6)°), the carbocycle disposed *trans* to the ether group (C(1)–Os–O(1) = 175.26(11)°), and the hydride ligand disposed *trans* to the chloride anion (H(01)–Os–Cl(1) = 172.7(12)°). The Os–C(1) bond length of 1.901(3) Å confirms the Os–C double bond.^24^ The presence of the hydride ligand in the molecule is also supported by the ^1^H NMR spectrum, in dichloromethane-*d*_2_, at 223 K, which contains a triplet (^2^*J*_H–P_ = 21.9 Hz) at −18.54 ppm. In the ^13^C{^1^H} NMR spectrum, the resonance corresponding to C(1) appears at 232.4 ppm. The ^31^P{^1^H} NMR spectrum displays a singlet at 53.3 ppm for the equivalent P^*i*^Pr_2_ groups.

**Figure 4:**
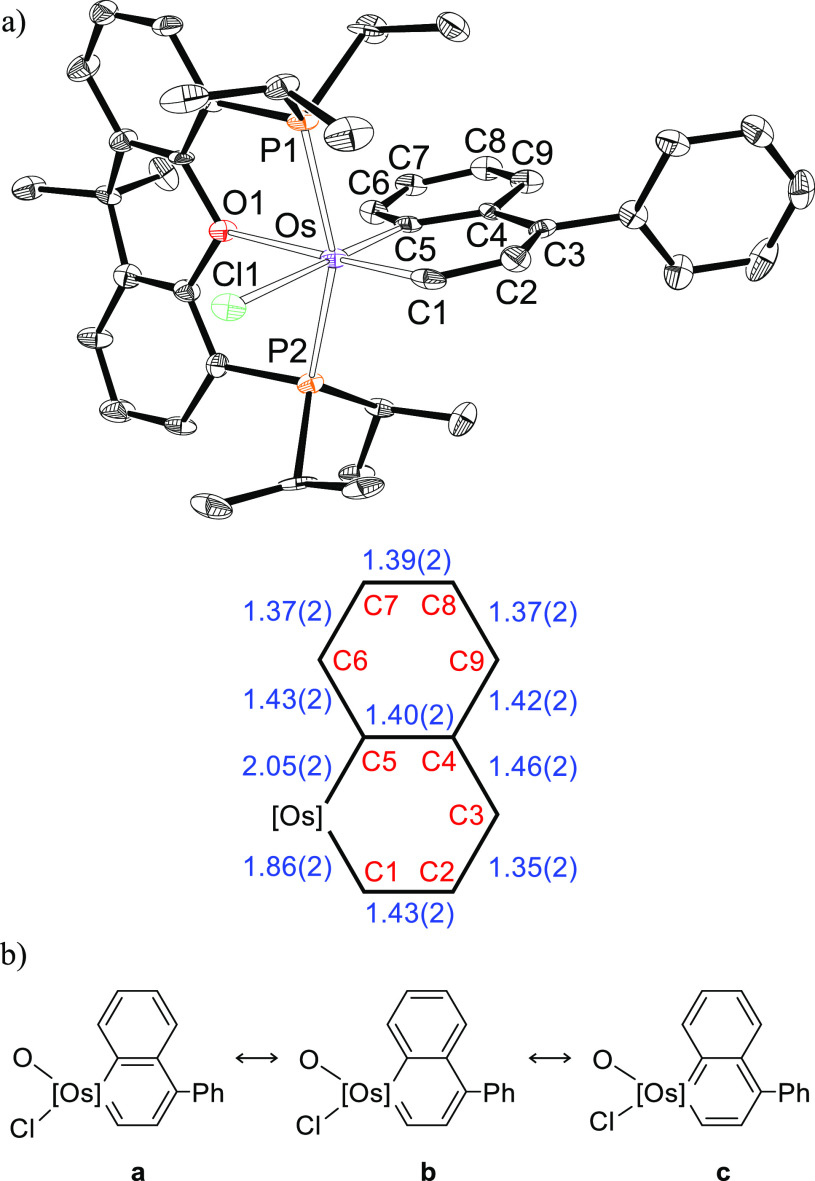
(a) Molecular diagram of complex **5** (ellipsoids shown at 50% probability). All hydrogen atoms are omitted for clarity. Selected bond distances (Å) and angles (deg): Os–P(1) = 2.353(4), Os–P(2) = 2.339(4), Os–O(1) = 2.322(10), Os–Cl(1) = 2.533(4), Os–C(1) = 1.861(18), Os–C(5) = 2.046(15), C(1)–C(2) = 1.43(2), C(2)–C(3) = 1.35(2), C(3)–C(4) = 1.46(2), C(4)–C(5) = 1.40(2), C(5)–C(6) = 1.431(19), C(6)–C(7) = 1.37(2), C(7)–C(8) = 1.39(2), C(8)–C(9) = 1.37(2), C(9)–C(4) = 1.42(2); P(1)–Os–P(2) = 156.79(13), C(1)–Os–O(1) = 178.1(5), C(5)–Os–Cl(1) = 177.5(4), P(1)–Os–O(1) = 78.9(3), and P(2)–Os–O(1) = 79.1(3). (b) Canonical forms that describe the metalacycle bonding situation.

Complex **5** is also certainly noticeable, since is a new member of the scarcely represented family of metalanaphthalene derivatives within the class of metalaaromatic compounds.^25^Figure 4a shows its structure, which proves the formation of the osmacycle. The coordination polyhedron around the osmium atom can be rationalized as a distorted octahedron with the ether-diphosphine κ^3^-*P*,*O*,*P*-*mer* coordinated (P(1)–Os–P(2) = 156.79(13)°, P(1)–Os–O(1) = 78.9(3)°, and P(2)–Os–O(1) = 79.1(3)°). The metalacycle is disposed perpendicular to an ideal P–Os–P direction with the C(1) atom of the OsC_5_ ring located *trans* to the oxygen atom (C(1)–Os–O(1) = 178.1(5)°) and the bridgehead C(5) atom situated *trans* to the chloride anion (C(5)–Os–Cl(1) = 177.5(4)°). The bond lengths in the bicycle reveal that from the three resonance forms contributing to its structure, **a**–**c** (Figure 4b), the form **a** is the most significant followed by **b**. Thus, the Os–C(1) distance of 1.861(18) Å is about 0.19 Å shorter than the Os–C(5) bond length of 2.046(15) Å, whereas bonds C(2)–C(3), C(4)–C(5), C(6)–C(7), and C(8)–C(9) are shorter than bonds C(3)–C(4), C(5)–C(6), C(7)–C(8), and C(9)–C(4) (1.35–1.40 Å versus 1.39–1.46 Å). With regard to the chloride anion, the ether linker of the diphosphine appears to have a stabilizing effect on the Os–C multiple bonds when it is situated *trans* to them. In this context, it should be noted that such disposition is observed in the four complexes, **2**–**5**, which could be related to the greater π-donor ability of oxygen with regard to chlorine and the π-acceptor capacity of the C-donor ligands. The existence of a markedly dominant resonance form can explain the low NICS(0) and NICS(1) values computed, 2.4 and −2.6 ppm, respectively, which are however in agreement with those found in other metalaaromatic complexes of this class.^26^ The ^13^C{^1^H} NMR spectrum of the green crystals, in dichloromethane-*d*_2_ at room temperature, also supports the dominant contribution of the resonance form **a** to the structure of the metalabicycle. In agreement with an almost double character of the Os–C(1) bond, the resonance corresponding to C(1) appears at 248.1 ppm, while the signal due to C(5) is observed at higher field, 168.9 ppm, as expected for an Os–C(sp^2^) almost single bond. The ^31^P{^1^H} NMR spectrum shows a singlet at 33.4 ppm, in accordance with the equivalence of the P^*i*^Pr_2_ groups disposed mutually *trans*.

**Scheme 5:**
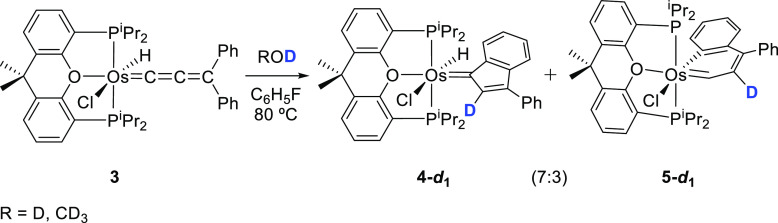
Isomerization of OsHCl(=C=C=CPh_2_){κ^3^-*P*,*O*,*P*-[xant(P^*i*^Pr_2_)_2_]} with Deuterated Water and Methanol-*d*_4_

Isomerization reactions from **3** into **4** and **5** are water-, alcohol-, and aldehyde-catalyzed competitive processes. The molar ratio between the isomeric products is independent of the catalyst. In order to understand this fact, we carried out the isomerization in the presence of D_2_O and methanol-*d*_4_. In both cases, we obtained the 7:3 mixture of the monodeuterated isomers **4**-***d***_**1**_ and **5**-***d***_**1**_ with the deuterium atom bonded to the C(2) atom of the compounds (Scheme 5); i.e., the C_β_ atom of the cumulene of **3**.

**Figure 5:**
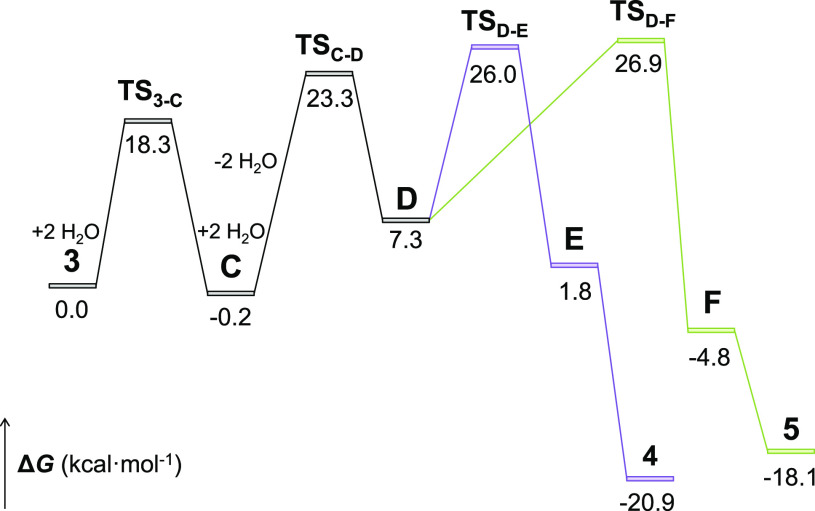
DFT-computed energy profile for complex **3** isomerization. Relative free energies (Δ*G* at 298.15 K) are given in kcal·mol^–1^ and were computed at the SMD(fluorobenzene)-B3LYP-D3//SDD(f)/6-31-G** level.

**Scheme 6:**
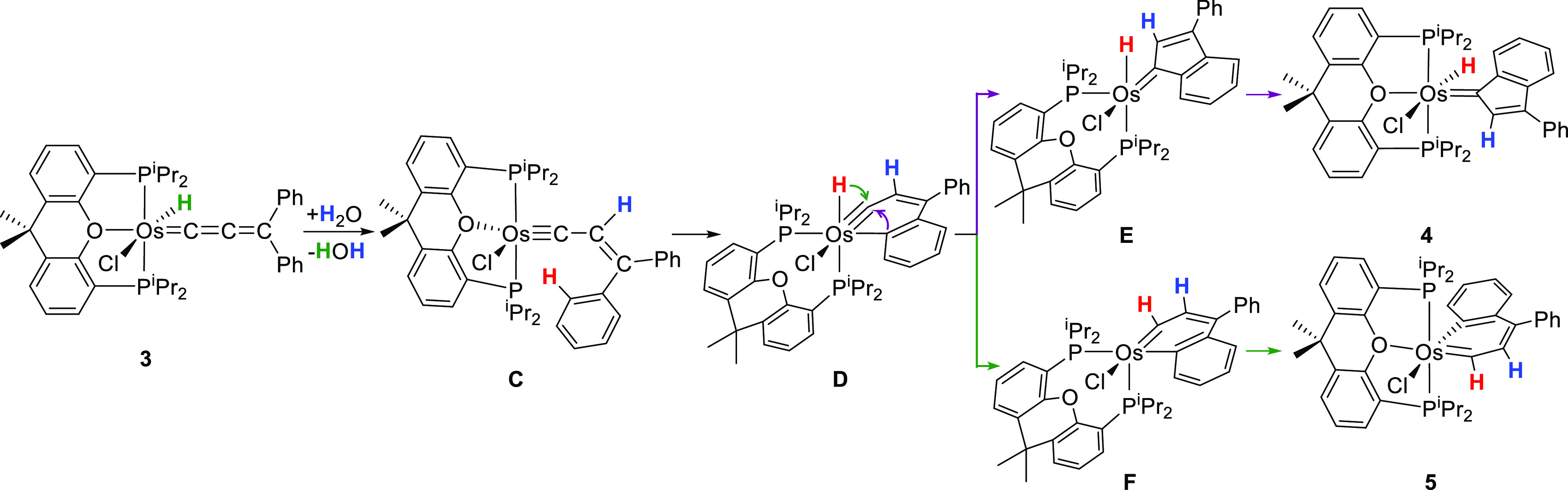
Mechanism of the Water-Catalyzed Isomerization of Complex **3**

The position of the deuterium atom, analogous in each compound, points out that the first step is common for both isomerization reactions and involves a catalyst-mediated 1,3-hydrogen shift from the metal center to the C_β_ atom of the cumulene of **3**. To gain insight about this unusual migration and the subsequent cyclization processes, we carried out DFT calculations at the dispersion-corrected SMD(fluorobenzene)-B3LYP-D3//SDD(f)/6-31-G** level (Figures S35–S37; see computational details in the Supporting Information). The changes in free energy (Δ*G*) were calculated at 298.15 K and 1 atm. Figure 5 shows the computed energy profile, whereas Scheme 6 gathers all the intermediates involved in the reaction.

The direct migration of the hydride to the C_β_ atom of the allenylidene ligand, through a four-center transition state, is energetically prohibited since it must be overcome a barrier of 71.4 kcal·mol^–1^ (Figure S35). The migration in two consecutive 1,2-hydrogen shifts, via an allenyl intermediate (Figure S36) is also energetically forbidden. Although the activation energy for the formation of the allenyl species is reduced to 28.1 kcal·mol^–1^, the transition state for the second migration lies 51.5 kcal·mol^–1^ over **3**. However, the proton shuttle formed by two water molecules consecutively associated by means of hydrogen bonds, significantly reduces the barrier for the 1,3-hydrogen shift to 18.3 kcal mol^–1^. The hydride ligand interacts with the oxygen atom of one of the water molecules to place a hydrogen atom of another close to the C_β_ atom of cumulene. This allows a cyclic transition state of eight-members (**TS**_**3-C**_; Figure S38), much less tensioned than that for the direct migration, which affords the five-coordinate osmium(0) intermediate **C**. In spite of its saturated character, the latter oxidatively adds the *ortho*-CH bond of a phenyl substituent of the alkenylcarbyne ligand in one step through the transition state **TS**_**C-D**_, which lies 23.3 kcal·mol^–1^ over **3**. The approach of the C–H bond to the metal center causes the dissociation of the oxygen atom of the ether-diphosphine, before of the C–H cleavage. The oxidative addition generates the osmanaphthalyne **D**, which is 7.3 kcal mol^–1^ less stable than **3**. The existence of this class of compounds has been experimentally demonstrated by Jia, Lin, and co-workers.^27^ Osmanaphthalyne **D** bears both fragments of the C–H bond activation disposed *cis* to the Os–C triple bond of the metalacycle. In agreement with the Jia and Lin calculations, the 1,2-carbon-migration leads to the hydride-osmium-indenylidene derivative **4**, whereas the 1,2-hydrogen-migration gives the osmanaphthalene **5**. The barriers are similar, 26.0 kcal mol^–1^ for the former and 26.9 kcal mol^–1^ for the second. As expected for the composition of the isomerization mixture, complex **4** is slightly more stable than **5**, 2.8 kcal mol^–1^. In spite of this small difference, they do not interconvert after isolation, as corresponds to the very high barrier for the isomerization on both sides. The intramolecular insertion reactions initially afford the respective five-coordinate species **E** and **F**, which subsequently coordinate the oxygen atom of the ether-diphosphine to yield the isomerization products.

The different behavior of **3** and the cation [OsH(=C=C=CPh_2_)(CH_3_CN)_2_(P^*i*^Pr_3_)_2_]^+^ toward alcohols is evident. On the basis of experimental observations and DFT results, this fact can be rationalized on both the difference in coordination ability between an acetonitrile ligand and the oxygen atom of the diphosphine and the difference in charge between the complexes. The greater coordination capacity of the ether group of the diphosphine with regard to the acetonitrile ligand prevents the coordination of the alcoholate, resulting from the protonation of the C_β_ atom of the cumulene. In this context, it should be mentioned that deuterium labeling experiments and theoretical calculations on the hydrogenation of the cation indicate that the β-hydrogen elimination in the coordinated alkoxide group is the key for the reduction, because the formation of a dihydride-carbyne species, with a *cis* disposition of the carbyne to both hydrides (*trans* between them), is essential to the 1,2-hydrogen shift from the metal center to the C_α_ atom of the carbyne (Scheme 1).^11a^ In addition, the neutral character of **3** with respect to the cationic nature of [OsH(=C=C=CPh_2_)(CH_3_CN)_2_(P^*i*^Pr_3_)_2_]^+^ increments the basicity of the metal center in the former, which increases the activation energy for the hydride migration from the metal center to the C-donor ligand^28^ and, at a time, favors the oxidative addition of the phenyl C–H bond in **C**.

### Protonation of **4** and **5**

Indenylidene complexes display a marked tendency to evolve to indenyl derivatives by 1,2-shift of an 1-e^–^ donor ligand, including chloride, from the metal center to the carbenic carbon atom.^27,29^ In addition, it has been argued that one of the difficulties in the synthesis of metalanaphthalene compounds could be due to its lower stability relative to the indenyl derivatives. So, the stability of **4** and **5** first surprised us and then encouraged us to promote their transformation to indenyl species, in particular that of **4**. In this context, we noted that the carbenic carbon atom of alkylidene-osmium(II) complexes has amphiphilic character, reacting with both nucleophiles and electrophiles, including H^+^.^30^ Thus, we decided to study the protonation of both **4** and **5**.

**Scheme 7:**
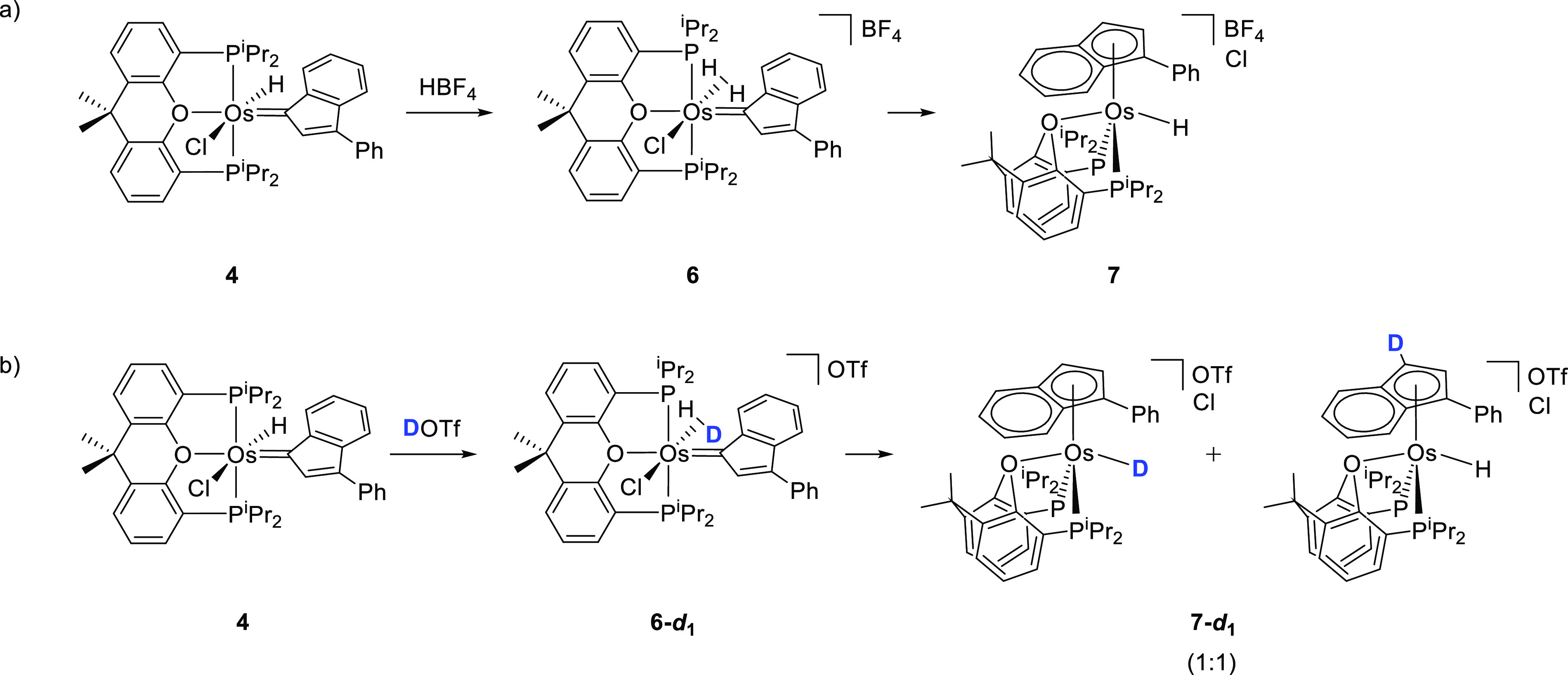
Protonation of Complex **4**

Addition of 1.0 equiv of HBF_4_ to a dichloromethane solution of **4** at 223 K immediate and quantitatively affords the elongated dihydrogen derivative [OsCl(η^2^-H_2_)(=C_IndPh_){κ^3^-*P*,*O*,*P*-[xant(P^*i*^Pr_2_)_2_]}]BF_4_ (**6**). In agreement with the presence of a coordinated hydrogen molecule in the complex, its ^1^H NMR spectrum shows a broad resonance at −6.04 ppm, which exhibits a 300 MHz *T*_1_(min) value of 31 ± 3 ms, at 217 K, whereas the H–D coupling constant in the partially deuterated species is 20 Hz. These values allow us to calculate H–H separations of 1.00 and 1.08 Å, respectively.^31^ A singlet at 257.0 ppm in the ^13^C{^1^H} NMR spectrum, due to the Os–C carbon atom, and a singlet at 46.5 ppm in the ^31^P{^1^H} NMR spectrum, corresponding to the equivalent P^*i*^Pr_2_ groups, are also characteristic features of this species. At room temperature, the acidic hydrogen atom of the dihydrogen ligand migrates to the carbenic carbon atom of the indenylidene, to generate an indenyl ligand. The migration is quantitative after 1 h and causes the formal oxidation of the metal center from Os^2+^ to Os^4+^. The generated indenyl ligand displaces the chloride anion from the metal coordination sphere to form [OsH(η^5^-IndPh){κ^3^-*P*,*O*,*P*-[xant(P^*i*^Pr_2_)_2_]}][BF_4_]Cl (**7** in Scheme 7a). In accordance with a dihydrogen-to-indenylidene migration of H^+^, the partially deuterated species **6**-***d***_**1**_ affords a 1:1 mixture of the **7**-***d***_**1**_ isomers shown in Scheme 7b.

**Figure 6:**
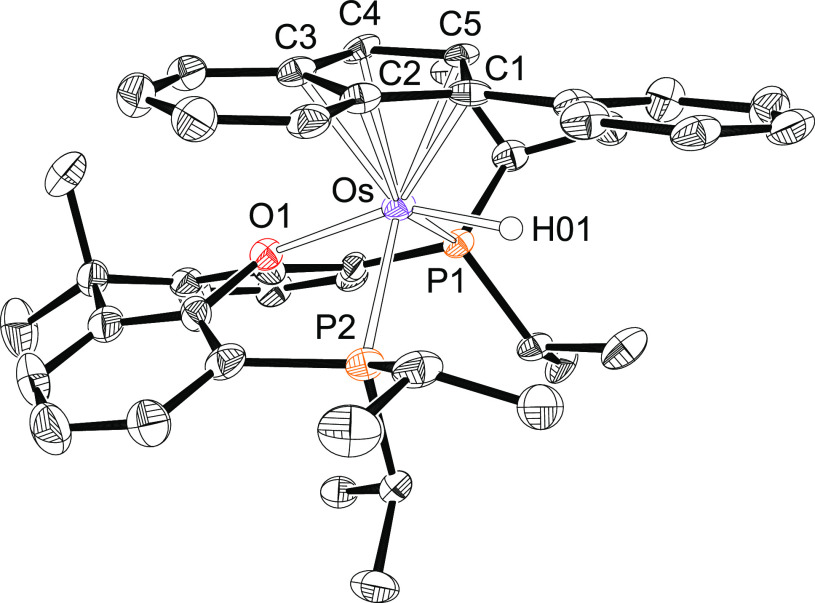
Molecular diagram of the cation of complex **7** (ellipsoids shown at 50% probability). All hydrogen atoms (except the hydride) are omitted for clarity. Selected bond distances (Å) and angles (deg): Os–P(1) = 2.313(2), Os–P(2) = 2.374(2), Os–O(1) = 2.163(5), Os–H(01) = 1.587(10), Os–C(1) = 2.255(7), Os–C(2) = 2.402(8), Os–C(3) = 2.358(8), Os–C(4) = 2.206(7), Os–C(5) = 2.180(7); P(1)–Os–P(2) = 107.32(7), O(1)–Os–H(01) = 150(3).

The double salt was isolated as orange crystals and characterized by X-ray diffraction analysis. Figure 6 gives a view of the cation. The distribution of ligands around the osmium atom can be described as a four-legged piano stool geometry. The indenyl ligand, which is coordinated by the five-membered ring, occupies the three-membered face while the ether-diphosphine and the hydride ligand lie in the four-membered face. The P^*i*^Pr_2_ groups are disposed in *transoid* position (P(1)–Os–P(2) = 107.32(7)°), and as a consequence, the oxygen atom and the hydride ligand must be situated in the other two vertices of the face (O(1)–Os–H(01) = 150(3)°). The P(1)–Os–P(2) angle compares well with the angles observed in other compounds bearing a κ^3^-*P*,*O*,*P*-*fac* coordinated ether-diphosphine,^14b,16^ even with those displaying a κ^2^-*P*,*P*-*cis* mode,^17^ and the P–Os–P angle usually found in osmium(IV) complexes with four-legged piano stool geometry and a *transoid* disposition of two phosphine ligands.^32^ However, it significantly deviates from the ideal angle for a P–Os–P *cis* disposition in an octahedral osmium(II) derivative. This difference could explain why the hydride complex **4** does not evolve to an indenyl-osmium(II) derivative, while the elongated dihydrogen compound **6** affords the double salt of the indenyl-osmium(IV) cation **7**. The influence of polydentate ligands bite angles on the coordination polyhedron of the complexes, and therefore on the stability of the different oxidation states of the central ion, is well demonstrated.^33^ The ^1^H and ^31^P{^1^H} NMR spectra, in dichloromethane-*d*_2_, at room temperature are consistent with the structure shown in Figure 6. Thus, the ^1^H contains at a doublet of doublets (^2^*J*_H–P_ = 36.1 and 32.4 Hz) at −12.57 ppm, due to the hydride ligand, whereas the ^31^P{^1^H} NMR displays two doublets (^2^*J*_P–P_ = 11.9 Hz) at −12.6 and −23.0 ppm, corresponding to the inequivalent P^*i*^Pr_2_ groups.

**Scheme 8:**
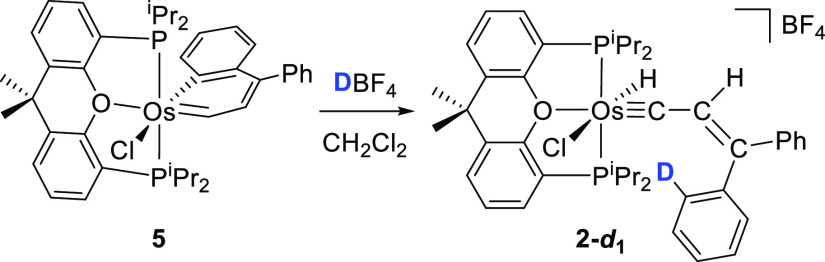
Protonation of Complex **5** Using DBF_4_

The osmanaphthalene complex **5** also reacts with HBF_4_. However, in contrast to **4**, the protonation regenerates the hydride-alkenylcarbyne **2** as a consequence of the attack of the proton to the bridgehead C(5) atom of the bicycle and an 1,2-hydrogen shift from C(1) to the metal center. According to this, the addition of DBF_4_ to the dichloromethane-*d*_2_ solution of **5** selectively leads to **2**-***d***_**1**_ containing a deuterium atom at one of the *ortho*-carbon atoms of a phenyl substituent of the alkenylcarbyne ligand (Scheme 8).

### Reaction of **3** with Phenylacetylene

**Scheme 9:**
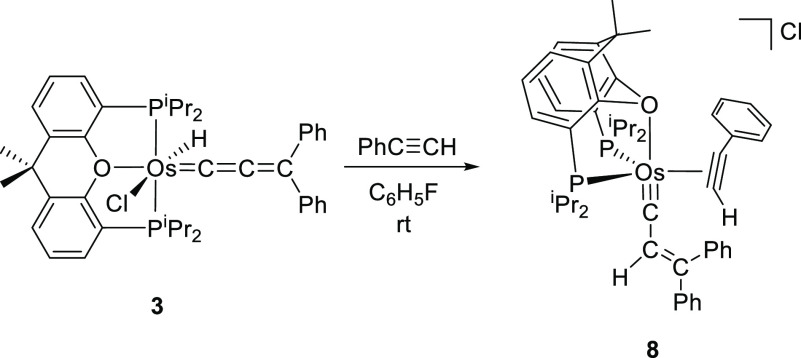
Reaction of Complex **3** with Phenylacetylene

Treatment of toluene solutions of hydride-osmium(II)-allenylidene complex with 4 equiv of the alkyne, for 2 days, at room temperature, leads to the π-alkyne-osmium(0)-alkenylcarbyne [Os(≡CCH=CPh_2_)(η^2^-HC≡CPh){κ^3^-*P*,*O*,*P*-[xant(P^*i*^Pr_2_)_2_]}]Cl (**8**). The salt was isolated as a brown solid in 58% yield. The reaction implies a 1,3-hydrogen shift from the metal to the C_β_ atom of the allenylidene ligand, which produces the Os^2+^-to-Os^0^ reduction of the central ion, the displacement of the chloride anion by the alkyne, and a change in the coordination of the ether-diphosphine from κ^3^-*P*,*O*,*P*-*mer* to κ^3^-*P*,*O*,*P*-*fac* (Scheme 9).

**Figure 7:**
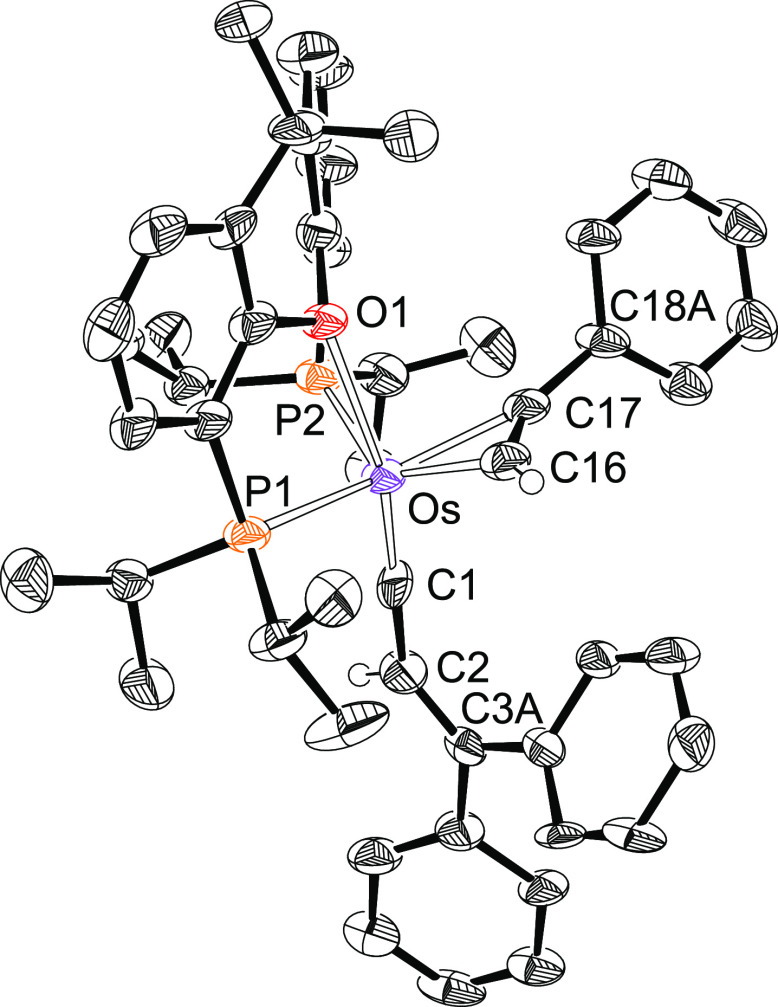
Molecular diagram of the cation of complex **8** (ellipsoids shown at 50% probability). All hydrogen atoms (except C_β_–H and the acetylenic hydrogen atoms) are omitted for clarity. Selected bond distances (Å) and angles (deg): Os–P(1) = 2.353(2), Os–P(2) = 2.363(3), Os–O(1) = 2.381(5), Os–C(1) = 1.708(10), Os–C(16) = 2.065(10), Os–C(17) = 2.098(9), C(1)–C(2) = 1.441(13), C(2)–C(3A) = 1.313(16), C(16)–C(17)) = 1.280(13); P(1)–Os–P(2) = 109.51(9), O(1)–Os–C(1) = 165.8(3).

Figure 7 shows a view of the structure of the cation, which proves the three previously mentioned transformations on the metal coordination sphere. The coordination polyhedron around the osmium atom can be rationalized as a distorted trigonal bipyramid with the oxygen atom of the diphosphine and the alkenylcarbyne ligand at the apexes (O(1)–Os–C(1) = 165.8(3)°), whereas the P^*i*^Pr_2_ groups and the C(16)–C(17) triple bond of the alkyne lie in the equatorial plane. The P(1)–Os–P(2) angle of 109.51(9) compares well with that of **7**, as expected, since the same coordination mode for the ether-diphosphine is observed in both complexes. The alkyne coordinates to the osmium atom with Os–C(16) and Os–C(17) distances of 2.065(10) and 2.098(9) Å, respectively. The coordination produces a slight elongation of the triple bond, according to the usual Chatt–Dewar–Ducanson bonding model. Thus, the C(16)–C(17) bond length of 1.280(13) Å is intermediate between triple and double bond. The osmium-carbyne bond length Os–C(1) of 1.708(10) Å and the C(1)–C(2) and C(2)–C(3A) distances of 1.441(13) and 1.313(16) Å, respectively, are similar to those of **2**. The ^1^H, ^13^C{^1^H}, and ^31^P{^1^H} NMR spectra, in dichloromethane-*d*_2_ at 253 K, are consistent with the structure shown in Figure 7. In the ^1^H, the most noticeable signals are a doublet (^3^*J*_H–P_ = 9.6 Hz) at 8.57 ppm, corresponding to the C(sp)–H hydrogen atom of the coordinated alkyne, and a singlet at 5.68 ppm due to the C(sp^2^)–H hydrogen atom of the alkenyl substituent of the carbyne. The ^13^C{^1^H} shows the resonance due to the C(1) alkenylcarbyne carbon atom at 265.0 ppm, whereas the signals assigned to the coordinated atoms of the alkyne are observed at 133.9 and 117.7 ppm. The ^31^P{^1^H} contains an AB spin system centered at 41.6 ppm and defined by Δν = 53.0 Hz and *J*_A–B_ = 16.9 Hz, in agreement with inequivalent P^*i*^Pr_2_ groups.

**Scheme 10:**
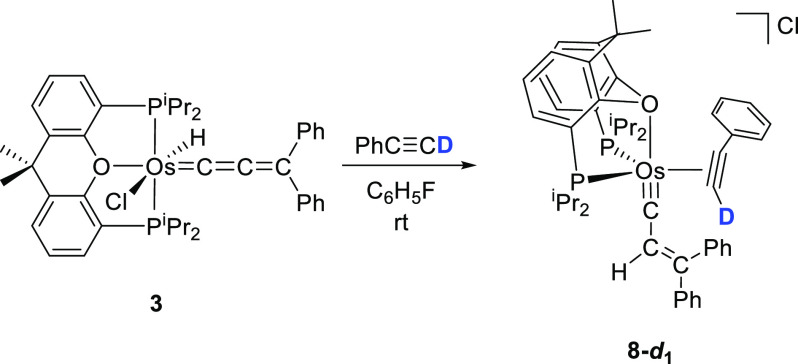
Reaction of **3** with Phenylacetylene-*d*_1_

The formation of **8** appears to be consistent with the isomerization of **3** to **4** and **5**, at least on an initial examination. Moreover, one could think that **8** is the result of trapping the intermediate **C** of Scheme 6 by means of the coordination of phenylacetylene. However, it should be noted that, although the C(sp)–H hydrogen atom of the alkyne is also fairly acidic, phenylacetylene has not an equivalent to the oxygen of the catalysts promoting the isomerization (water, alcohols, and aldehydes) to interact with the hydride ligand of **3** and to approach the acidic proton to the C_β_ atom of the cumulene. In view of this inconsistency, we decided to carry out the reaction of **3** with PhC≡CD. Under the same conditions as that employed to form **8**, **8**-***d***_**1**_ was quantitative and selectively obtained (Scheme 10).

**Scheme 11:**
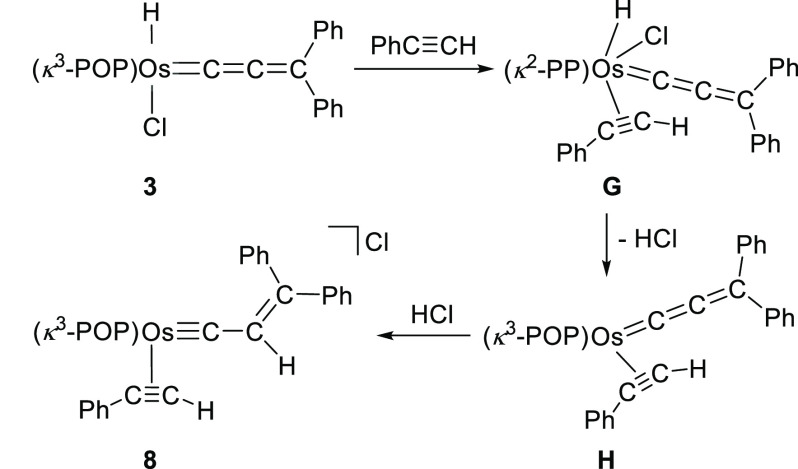
Proposed Mechanism for the Formation of Complex **8**

The position of the deuterium atom at the coordinated alkyne of **8**-***d***_**1**_ indicates that the 1,3-hydrogen shift in this case occurs by a different manner to those previously discussed. A feasible alternative could involve the reductive elimination of HCl as consequence of the acidification of the metal center, due to the initial replacement of the oxygen atom of the ether-diphosphine by the alkyne. Once the reduction has taken place, the recoordination of the ether linker, now κ^3^-*P*,*O*,*P*-*fac* with the oxygen atom *trans* to the cumulene, and the subsequent protonation of the C_β_ atom of the latter with the displaced HCl should yield **8** (Scheme 11).

## Concluding Remarks

This study has revealed the existence of a 1,3-hydrogen shift in the elusive hydride-metal-allenylidene complexes, which is responsible for the isomerization of the cumulene to indenylidene^34^ and the transformation of the hydride-allenylidene unit into the metalaaromatic bicycle metalanaphthalene. The hydrogen shift, which has an activation energy too high to occur in a concerted manner, is catalyzed by water, alcohols, and aldehydes. Phenylacetylene also provokes the 1,3-hydrogen shift; however, it does not participate in the migration. In contrast to water, alcohols, and aldehydes, it stabilizes the resulting alkenylcarbyne, preventing its evolution into indenylidene or metalanaphthalene.

This study has also illustrated a new behavior of transition metal allenylidene complexes toward alcohols. Until now, these compounds had shown three different conducts. Those with electrophilic nature form α,β-unsaturated alkoxycarbene derivatives, as a result of the 1,2-addition of the O–H bond of the alcohols to the C_α_–C_β_ double bond of the allenylidene, nucleophilic allenylidenes are inert,^1a^ and the cation [OsH(=C=C=CPh_2_)(CH_3_CN)_2_(P^*i*^Pr_3_)_2_]^+^ undergoes reduction of the C_α_–C_β_ double bond as a consequence of a hydrogen transfer reaction from the alcohol to the complex.^11a^ The transformation of **3** into **4** and **5** represents an alternative conduct, alcohol-induced isomerization.

It was thought so far that the reactivity of an allenylidene ligand was only a consequence of its nucleophilicity or electrophilicity, which is imposed by the coligands of the complex. Thus, allenylidene ligands of similar electronic nature should display analogous behavior. Complex **3** and the cation [OsH(=C=C=CPh_2_)(CH_3_CN)_2_(P^*i*^Pr_3_)_2_]^+^ bear allenylidene ligands, which have a common characteristic: the strong nucleophilic character of the central carbon atom of the C_3_-chain. Nevertheless, they show different behavior due to different abilities of the coligands. The poor coordinating capacity of the acetonitrile ligand of the cation allows the reduction of the C_α_–C_β_ double bond, while the association of the hydride ligand of **3** with the oxygen atom of the alcohol permits to lower the activation barrier for the 1,3-hydrogen shift from the metal to the C_β_ atom of the cumulene; i.e., the coligands of allenylidene complexes are not innocent; they can have a direct participation in the reactions of the C_3_-chain.

In summary, a new reactivity pattern for hydride-allenylidene complexes has been observed, which is of interest in connection with the isomerization phenylallenylidene-to-indenylidene and the transformation hydride, indenylidene-to-indenyl. Furthermore, it can help to systematize the preparation of metalanaphthalene derivatives.

## Experimental Section

### General Information

All reactions were carried out with exclusion of air using Schlenk-tube techniques or in a drybox. Instrumental methods and X-ray details are given in the Supporting Information. In the NMR spectra (Figures S1–S34) the chemical shifts (in ppm) are referenced to residual solvent peaks (^1^H, ^13^C{^1^H}) or external 85% H_3_PO_4_ (^31^P{^1^H}). Coupling constants *J* and *N* (*N* = *J*_P–H_ + *J*_P′–H_ for ^1^H and *N* = *J*_P–C_ + *J*_P′–C_ for ^13^C{^1^H}) are given in hertz. [(Os(H···H){κ^3^-*P*,*O*,*P*-[xant(P^*i*^Pr_2_)_2_]})_2_(μ-Cl)_2_][BF_4_]_2_ (**1**) was prepared as previously reported.^16^

### Preparation of [OsHCl(≡CCH=CPh_2_){κ^3^-*P*,*O*,*P*-[xant(P^*i*^Pr_2_)_2_]}]BF_4_ (**2**)

Complex **1** (500 mg, 0.33 mmol) in fluorobenzene (4 mL) was treated with 1,1-diphenyl-2-propyn-1-ol (550 mg, 2.64 mmol), in the presence of 4 Å molecular sieves (2 g). After 2h, at 80 °C, the resulting suspension was separated from the molecular sieves by decantation with a canule. Then, the liquid phase was removed and the dark red solid was washed with diethyl ether (3 × 2 mL) and pentane (6 × 3 mL) and dried under vacuum. Yield: 475 mg (76%). Crystals suitable for X-ray diffraction analysis were obtained by slow cooling of a fluorobenzene solution from 80 °C to room temperature. Anal. Calcd for C_42_H_52_BClF_4_OOsP_2_: C, 53.25; H, 5.53. Found: C, 52.85; H, 5.36. HRMS (electrospray, *m*/*z*) calcd. for C_42_H_52_ClOOsP_2_ [M]^+^: 861.2797, found 861.2764. IR (cm^–1^) ν(Os–H) 2142 (w); ν(C=C) 1531 (m); ν(BF_4_) 1048 (s). ^1^H NMR (400.16 MHz, CD_2_Cl_2_, 298 K) δ 7.70–7.35 (m, 16H, CH-arom), 5.55 (s, 1H, Os≡C—C*H*), 3.09 (m, 2H, PC*H*(CH_3_)_2_), 2.66 (m, 2H, PC*H*(CH_3_)_2_), 1.70 (s, 3H, C(CH_3_)_2_), 1.52 (s, 3H, C(CH_3_)_2_), 1.45 (dvt, ^3^*J*_H–H_ = 7.2, *N* = 15.9, 6H, PCH(C*H*_3_)_2_), 1.41 (dvt, ^3^*J*_H–H_ = 7.1, *N* = 15.2, 6H, PCH(C*H*_3_)_2_), 1.23 (dvt, ^3^*J*_H–H_ = 6.8, *N* = 18.5, 6H, PCH(C*H*_3_)_2_), 1.05 (dvt, ^3^*J*_H–H_ = 6.9, *N* = 16.9, 6H, PCH(C*H*_3_)_2_), −5.59 (t, ^2^*J*_H–P_ = 16.4, 1H, OsH). ^13^C{^1^H}-APT NMR (100.64 MHz, CD_2_Cl_2_, 298 K) δ 271.5 (t, ^2^*J*_C–P_ = 5.6, Os≡C), 166.9 (s, C(Ph)_2_), 154.2 (vt, *N* = 10.8, C-arom, POP), 139.3 and 137.8 (both s, C-ipso, Ph), 132.8 (vt, *N* = 5.5, C-arom, POP), 132.6 (s, CH-arom, POP), 132.1 and 132.0 (both s, *p*-CH, Ph), 131.8 and 131.7 (both s, *m*-CH, Ph), 130.8 (s, Os≡C—*C*H), 129.7 and 129.8 (both s, *o*-CH, Ph), 129.5 (s, CH-arom, POP), 127.5 (vt, *N* = 7.0, CH-arom, POP), 120.7 (vt, *N* = 41.5, C-arom, POP), 34.7 (s, *C*(CH_3_)_2_), 34.5 (s, C(*C*H_3_)_2_), 31.4 (s, C(*C*H_3_)_2_), 30.1 (vt, *N* = 34.6, P*C*H(CH_3_)_2_), 29.6 (vt, *N* = 27.5, P*C*H(CH_3_)_2_), 21.5 and 19.7 (both s, PCH(*C*H_3_)_2_). ^31^P{^1^H} NMR (121.49 MHz, CD_2_Cl_2_, 298 K) δ 52.9 (s). ^19^F{^1^H} NMR (376.49 MHz, CD_2_Cl_2_, 298 K) δ −153.1 (br).

### Preparation of OsHCl(=C=C=CPh_2_){κ^3^-*P*,*O*,*P*-[xant(P^*i*^Pr_2_)_2_]} (**3**)

Complex **2** (500 mg, 0.53 mmol) and K^*t*^BuO (65 mg, 0.58 mmol) were dissolved in precooled THF (10 mL, −30 °C). The green solution was stirred for 1 h at this temperature. Then, it was warmed to room temperature and the solvent was removed under a vacuum. The residue was treated with dichloromethane (5 mL). The resulting suspension was filtered through Celite. The dark solution was concentrated under reduced pressure. Addition of acetonitrile (5 mL) afforded a green solid, that was washed further with acetonitrile (3 × 3 mL) and dried under a vacuum. Yield: 390 mg (86%). Crystals suitable for X-ray diffraction analysis were obtained by slow diffusion of pentane into a saturated dichloromethane solution at −4 °C. Anal. Calcd for C_42_H_51_ClOOsP_2_: C, 58.69; H, 5.98. Found: C, 59.02; H, 6.04. HRMS (electrospray, *m*/*z*) calcd. for C_42_H_52_ClOOsP_2_ [M + H]^+^: 861.2797, found 861.2742. IR (cm^–1^) ν(Os–H) 2090 (w); ν(Os=*C*=*C*) 1863 (m); ν(C=C) 1395 (s). ^1^H NMR (400.13 MHz, CD_2_Cl_2_, 298 K) δ 7.72 (d, ^3^*J*_H–H_ = 7.2, 4H, *o*-CH, Ph), 7.60 (t, ^3^*J*_H–H_ = 7.4, 2H, *p-*CH, Ph), 7.55 (d, ^3^*J*_H–H_ = 7.7, 2H, CH-arom, POP), 7.52 (m, 2H, CH-arom, POP), 7.30 (t, ^3^*J*_H–H_ = 7.6, 2H, CH-arom, POP), 7.22 (t, ^3^*J*_H–H_ = 7.8, 4H, *m*-CH, Ph), 2.99 (m, 2H, PC*H*(CH_3_)_2_), 2.56 (m, 2H, PC*H*(CH_3_)_2_), 1.80 (s, 3H, C(CH_3_)_2_), 1.51 (dvt, ^3^*J*_H–H_ = 7.2, *N* = 14.8, 6H, PCH(C*H*_3_)_2_), 1.51 (s, 3H, C(CH_3_)_2_), 1.44 (dvt, ^3^*J*_H–H_ = 7.5, *N* = 15.8, 6H, PCH(C*H*_3_)_2_), 1.20 (dvt, ^3^*J*_H–H_ = 7.2, *N* = 16.1, 6H, PCH(C*H*_3_)_2_), 0.89 (dvt, ^3^*J*_H–H_ = 7.1, *N* = 14.9, 6H, PCH(C*H*_3_)_2_), −8.82 (t, ^2^*J*_H–P_ = 17.4, 1H, OsH). ^13^C{^1^H}-APT NMR (100.64 MHz, CD_2_Cl_2_, 298 K) δ 256.1 (t, ^3^*J*_C–P_ = 4.1, Os=C=*C*), 242.5 (t, ^2^*J*_C–P_ = 10.4, Os=C), 157.0 (vt, *N* = 12.4, C-arom, POP), 154.8 (t, ^4^*J*_C–P_ = 2.4, Os=C=C=*C*), 132.2 (vt, *N* = 5.4, C-arom, POP), 131.6 (s, CH-arom, POP), 129.1 (s, *m*-CH, Ph), 129.0 (s, C-arom, POP), 128.9 (s, CH-arom, POP), 126.3 (s, *o*-CH, Ph), 126.3 (s, *p*-CH, Ph), 125.9 (vt, *N* = 5.0, CH-arom, POP), 120.4 (s, C-ipso, Ph), 35.6 (s, C(*C*H_3_)_2_), 34.7 (s, *C*(CH_3_)_2_), 29.5 (s, C(*C*H_3_)_2_), 29.3 (vt, *N* = 23.4, P*C*H(CH_3_)_2_), 28.7 (vt, *N* = 30.9, P*C*H(CH_3_)_2_), 22.2 (s, PCH(*C*H_3_)_2_), 20.0 (s, PCH(*C*H_3_)_2_), 19.6 (vt, *N* = 5.6, PCH(*C*H_3_)_2_), 19.3 (vt, *N* = 2.8, PCH(*C*H_3_)_2_). ^31^P{^1^H} NMR (121.49 MHz, CD_2_Cl_2_, 298 K) δ 27.6 (s).

### Preparation of OsHCl(=C_IndPh_){κ^3^-*P*,*O*,*P*-[xant(P^*i*^Pr_2_)_2_]} (**4**)

Complex **3** (500 mg, 0.58 mmol) was dissolved in fluorobenzene (5 mL). Then, 10 μL of water, methanol, 2-propanol or benzaldehyde (1–2 equiv) were added. The solution was heated at 80 °C during 16 h, to form a 7:3 mixture of **4** and **5**. The solvent was removed under a vacuum and the residue was initially washed with methanol (3 × 2 mL) and subsequently with diethyl ether (3 mL) and dried under a vacuum, to afford **4** as a brownish solid. Yield: 320 mg (64%). Crystals suitable for X-ray diffraction analysis were obtained from a saturated diethyl ether solution at −18 °C. Anal. Calcd for C_42_H_51_ClOOsP_2_: C, 58.69; H, 5.98. Found: C, 58.72; H, 5.89. HRMS (electrospray, *m*/*z*) calcd. for C_42_H_51_OOsP_2_ [M – Cl]^+^: 825.3024, found 825.3005. IR (cm^–1^) ν(Os–H) 2164 (w); ν(C=C) 1402 (s). ^1^H NMR (300.13 MHz, CD_2_Cl_2_, 223 K) δ 8.29 (d, ^3^*J*_H–H_ = 7.4, 1H, CH-arom, Ind), 7.77 (d, ^3^*J*_H–H_ = 7.4, 2H, CH-arom, POP), 7.54 (m, 3H, CH-arom, Ph + Ind), 7.52 (s, 1H, C_β_–H), 7.43–7.34 (m, 6H, CH-arom, POP + Ind), 7.25 (t, ^3^*J*_H–H_ = 7.5, 2H, CH-arom, POP), 7.19 (t, ^3^*J*_H–H_ = 7.4, 1H, Ind), 3.06 (m, 2H, PC*H*(CH_3_)_2_), 2.45 (m, 2H, PC*H*(CH_3_)_2_), 1.70, 1.61 (both s, 3H, C(CH_3_)_2_), 1.03 (m, 12H, PCH(C*H*_3_)_2_), 0.89 (dvt, ^3^*J*_H–P_ = 7.3, *N* = 16.1, 6H, PCH(C*H*_3_)_2_), 0.62 (dvt, ^3^*J*_H–P_ = 7.4, *N* = 16.5, 6H, PCH(C*H*_3_)_2_), −18.54 (t, ^2^*J*_H–P_ = 21.9, 1H, OsH). ^13^C{^1^H}-APT NMR (75.48 MHz, CD_2_Cl_2_, 223 K) δ 232.4 (s, Os=C), 158.9 (s, C, Ind), 154.6 (vt, *N* = 11.9, C-arom, POP), 146.7 (s, C_β_–H, Ind), 143.1 (s, C, Ind), 138.1 (s, C-ipso, Ph), 135.4 (s, C, Ind), 131.2 (vt, *N* = 2.4, C-arom, POP), 130.4, 129.3 (both s, CH, Ph), 129.2 (s, CH-arom, POP), 127.3, 127.0 (both s, CH, Ind), 126.3 (s, CH-arom, POP), 125.9, 125.5 (both s, CH, Ind), 124.8 (vt, *N* = 5.2, CH-arom, POP), 123.4 (vt, *N* = 35.6, C-arom, POP), 118.5 (s, CH, Ph), 34.5 (s, C(*C*H_3_)_2_), 34.3 (s, *C*(CH_3_)_2_), 31.9 (vt, *N* = 21.0, P*C*H(CH_3_)_2_), 30.9 (s, C(*C*H_3_)_2_), 29.6 (vt, *N* = 38.2, P*C*H(CH_3_)_2_), 19.0, 18.8, 17.5 (all s, PCH(*C*H_3_)_2_), 17.0 (vt, N = 6.2, PCH(*C*H_3_)_2_). ^31^P{^1^H} NMR (121.50 MHz, CD_2_Cl_2_, 223 K) δ 53.3 (s).

### Preparation of OsCl[C_9_H_6_Ph]{κ^3^-*P*,*O*,*P*-[xant(P^*i*^Pr_2_)_2_]} (**5**)

Complex **5** was isolated from the residue obtained as previously mentioned, by silica gel column chromatography using diethyl ether as eluent. Green solid; yield: 120 mg (24%). Crystals suitable for X-ray diffraction analysis were obtained from a saturated methanol solution at −18 °C. Anal. Calcd for C_42_H_51_ClOOsP_2_: C, 58.69; H, 5.98. Found: C, 58.30; H, 5.85. HRMS (electrospray, *m*/*z*) calcd. for C_42_H_51_OOsP_2_ [M – Cl]^+^: 825.3024, found 825.3022. IR (cm^–1^) ν(C–O–C) 1105 (m); ν(C=C) 1398 (s). ^1^H NMR (300.13 MHz, CD_2_Cl_2_, 298 K) δ 7.70 (m, 3H, CH-arom, POP + PhN), 7.56 (d, ^3^*J*_H–H_ = 8.1, 1H, PhN), 7.40 (m, 6H, CH-arom, POP + PhN), 7.28 (m, 5H, POP + PhN), 6.54 (t, ^3^*J*_H–H_ = 7.3, 1H, PhN), 6.23 (t, ^3^*J*_H–H_ = 7.1, 1H, PhN), 2.92 (m, 2H, PC*H*(CH_3_)_2_), 2.26 (m, 2H, PC*H*(CH_3_)_2_), 1.88 (s, 3H, C(CH_3_)_2_), 1.84 (s, 3H, C(CH_3_)_2_), 1.35 (dvt, ^3^*J*_H–P_ = 7.6, *N* = 15.8, 6H, PCH(C*H*_3_)_2_), 1.27 (dvt, ^3^*J*_H–P_ = 7.7, *N* = 15.3, 6H, PCH(C*H*_3_)_2_), 0.59 (dvt, ^3^*J*_H–P_ = 7.2, *N* = 15.3, 6H, PCH(C*H*_3_)_2_), −0.01 (dvt, ^3^*J*_H–P_ = 7.3, *N* = 14.9, 6H, PCH(C*H*_3_)_2_). ^13^C{^1^H}-APT NMR (75.48 MHz, CD_2_Cl_2_, 298 K) δ 248.1 (s, CH, Os=CH), 168.9 (s, C, Os–C), 158.7 (s, C, PhN), 154.6 (s, C-arom, POP), 145.8 (s, C, PhN), 144.6, 140.4, 139.0, 136.2 (all s, CH, PhN), 133.7 (s, CH-arom, POP), 132.4 (s, C-arom, POP), 130.0 (s, CH, PhN), 129.7 (s, CH-arom, POP), 129.1 (s, CH, PhN), 128.3 (s, CH-arom, POP), 127.0 (s, CH, PhN), 124.8 (s, CH-arom, POP), 124.3 (vt, *N* = 32.5, C-arom, POP), 117.7 (s, CH, PhN), 35.7 (s, C(*C*H_3_)_2_), 35.1 (s, *C*(CH_3_)_2_), 32.8 (s, C(*C*H_3_)_2_), 30.4 (vt, *N* = 28.1, P*C*H(CH_3_)_2_), 24.1 (vt, *N* = 28.0, P*C*H(CH_3_)_2_), 22.1, 19.2, 18.8 (all s, PCH(*C*H_3_)_2_). ^31^P{^1^H} NMR (121.50 MHz, CD_2_Cl_2_, 298 K) δ 33.4 ppm (s).

### Formation of a Mixture of OsHCl(=C_IndPh_){κ^3^-*P*,*O*,*P*-[xant(P^*i*^Pr_2_)_2_]}-*d*_1_ (4-*d*_1_) and OsCl[C_9_H_5_DPh]{κ^3^-*P*,*O*,*P*-[xant(P^*i*^Pr_2_)_2_]} (**5**-*d*_1_)

Two screw-top NMR tubes were charged with **3** (50 mg, 0.058 mmol). Then, 0.4 mL of dichloromethane-*d*_2_ were added to one of them and 0.4 mL of dichloromethane to the other. Subsequently, the solutions were treated with 2 μL of D_2_O or methanol-*d*_4_ (1–2 equiv). After 16 h, at 80 °C, ^31^P{^1^H} NMR spectra showed quantitative formation of **4**-***d***_**1**_ and **5**-***d***_**1**_ in a 7:3 molar ratio. HRMS (TIMS-electrospray-TOF, *m*/*z*) calcd. for C_42_H_50_DOOsP_2_ [M – Cl]^+^: 826.3087, found 826.3073 and CSS (Å^2^): 264.9 (**4**-***d***_**1**_) and 256.6 (**5**-***d***_**1**_). ^2^H NMR (61.42 MHz, CH_2_Cl_2_, 298 K) δ 7.60 (br, **4**-***d***_**1**_), 7.36 (br, **5**-***d***_**1**_).

### Preparation of [Os(η^2^-H_2_)Cl(=C_IndPh_){κ^3^-*P*,*O*,*P*-[xant(P^*i*^Pr_2_)_2_]}]BF_4_ (**6**)

A solution of **4** (50 mg, 0.058 mmol) in dichloromethane-*d*_2_ (0.5 mL), contained in a screw top NMR tube, was cooled at 195 K and treated with HBF_4_·OEt_2_ (8.8 μL, 0.064 mmol). Immediately it was introduced into a precooled NMR probe at 223 K and its spectra were recorded at this temperature. Quantitative and immediate formation of **6** was observed. ^1^H NMR (400.13 MHz, CD_2_Cl_2_, 223 K) δ 8.04 (s, 1H, CH-arom), 7.82 (m, 2H, CH-arom), 7.75 (m, 2H, CH-arom), 7.63 (t, ^3^*J*_H–H_ = 8.1, 2H, CH-arom), 7.51–7.40 (m, 7H, CH-arom), 7.37 (m, 2H, CH-arom), 3.30 (m, 2H, PC*H*(CH_3_)_2_), 2.56 (m, 2H, PC*H*(CH_3_)_2_), 1.74 (s, 3H, C(CH_3_)_2_), 1.57 (s, 3H, C(CH_3_)_2_), 1.42–1.30 (m, 6H, PCH(C*H*_3_)_2_), 1.10–0.87 (m, 18H, PCH(C*H*_3_)_2_), −6.04 (br, 2H, OsH). *T*_1_(min) (ms, OsH, 300 MHz, CD_2_Cl_2_, 217 K): 31 ± 3 (−6.04 ppm). ^13^C{^1^H}-APT NMR (100.63 MHz, CD_2_Cl_2_, 223 K) δ 257.0 (s, Os=C), 153.9 (vt, *N* = 9.6, C-arom, POP), 150.9, 146.7 (both s, CH-arom), 143.1, 141.1 (both s, C-arom), 133.8, 132.7 (both s, CH-arom), 132.3 (vt, *N* = 5.1, CH-arom, POP), 131.5, 131.3, 130.3, 129.5 (all s, CH-arom), 129.2, 127.0 (both s, C-arom), 126.8 (s, CH-arom), 126.6 (m, CH-arom, POP), 122.6 (s, CH-arom), 118.8 (vt, *N* = 42.9, CH-arom, POP), 34.2 (s, *C*(CH_3_)_2_), 33.7, 30.8 (both s, C(*C*H_3_)_2_), 29.7 (vt, *N* = 33.8, P*C*H(CH_3_)_2_), 28.8 (vt, *N* = 26.8, P*C*H(CH_3_)_2_), 19.7, 18.7, 18.0, 17.4 (all s, PCH(*C*H_3_)_2_). ^31^P{^1^H} NMR (161.98 MHz, CD_2_Cl_2_, 223 K) δ 46.5 (s).

### Preparation of [Os(η^2^-H-D)Cl(=C_IndPh_){κ^3^-*P*,*O*,*P*-[xant(P^*i*^Pr_2_)_2_]}]OTf (**6**-*d*_1_)

Two screw-top NMR tubes were charged with **4** (50 mg, 0.058 mmol). Then, 0.4 mL of dichloromethane-*d*_2_ was added to one of them and 0.4 mL of dichloromethane was added to the other. Subsequently, the solutions were cooled at 195 K and treated with trifluoromethanesulfonic acid-*d* (5.7 μL, 0.064 mmol). Immediately, they were introduced into a precooled NMR probe at 223 K and their spectra were recorded at this temperature. Quantitative and immediate conversion to **6-***d*_1_ was observed. ^1^H NMR (400.13 MHz, CD_2_Cl_2_, 253 K, high-field region) δ −6.00 (m, ^1^*J*_H–D_ = 20.1, ^2^*J*_H–P_ = 8.5, 1H, OsH). ^1^H{^31^P} NMR (400.13 MHz, CD_2_Cl_2_, 253 K, high-field region) δ −6.00 (t, ^1^*J*_H–D_ = 20.1, 1H, OsH). ^2^H NMR (61.42 MHz, CH_2_Cl_2_, 223 K) δ −5.86 (m).

### Preparation of [OsH(η^5^-IndPh){κ^3^-*P*,*O*,*P*-[xant(P^*i*^Pr_2_)_2_]}][BF_4_]Cl (**7**)

A solution of **4** (200 mg, 0.23 mmol) in dichloromethane (5 mL) was treated with HBF_4_·OEt_2_ (35 μL, 0.26 mmol) and stirred at room temperature for 1 h. After that time, the solution was concentrated to ca. 0.5 mL and diethyl ether (3 mL) was added to form an orange precipitate, which was washed with diethyl ether (3 × 3 mL) and dried under a vacuum. Orange solid; yield: 75 mg (34%). Crystals suitable for X-ray diffraction analysis were obtained from slow diffusion of pentane into a saturated 1,2-dichloroethane solution. Anal. Calcd for C_42_H_52_BClF_4_OOsP_2_: C, 53.25; H, 5.53. Found: C, 53.27; H, 5.69. HRMS (electrospray, *m*/*z*) calcd. for C_42_H_52_ClOOsP_2_ [M + Cl]^+^: 861.2791, found 861.2812. IR (cm^–1^) ν(Os–H) 1938 (w); ν(BF_4_) 1049 (s). ^1^H NMR (300.13 MHz, CD_2_Cl_2_, 298 K) δ 7.93 (t, ^3^*J*_H–H_ = 7.5, 1H, CH-arom), 7.84 (d, ^3^*J*_H–H_ = 8.4, 1H, CH-arom), 7.70–7.59 (m, 2H, CH-arom), 7.49 (d, ^3^*J*_H–H_ = 7.4, 1H, CH-arom), 7.43–7.30 (m, 3H, CH-arom), 7.21 (t, ^3^*J*_H–H_ = 7.2, 1H, CH-arom), 7.07 (t, ^3^*J*_H–H_ = 7.0, 1H, CH-arom), 6.89 (t, ^3^*J*_H–H_ = 7.2, 1H, CH-arom), 6.56 (t, ^3^*J*_H–H_ = 7.5, 2H, CH-arom), 6.26 (s, 1H, CH-arom), 5.96 (d, ^3^*J*_H–H_ = 7.4, 2H, CH-arom), 5.73 (s, 1H, CH-arom), 3.48–3.30 (m, 2H, PC*H*(CH_3_)_2_), 3.30–3.10 (m, 2H, PC*H*(CH_3_)_2_), 2.10 (dd, ^3^*J*_H–P_ = 16.3, ^3^*J*_H–H_ = 6.8, 3H, PCH(C*H*_3_)_2_), 1.79 (s, 3H, C(CH_3_)_2_), 1.85–1.69 (m, 3H, PCH(C*H*_3_)_2_), 1.85–1.69 (m, 3H, PCH(C*H*_3_)_2_) 1.58 (dd, ^3^*J*_H–P_ = 15.5, ^3^*J*_H–H_ = 7.2, 3H, PCH(C*H*_3_)_2_), 1.64–1.52 (m, 3H, PCH(C*H*_3_)_2_), 1.44 (s, 3H, C(CH_3_)_2_), 1.12 (dd, ^3^*J*_H–P_ = 15.5, ^3^*J*_H–H_ = 7.2, 3H, PCH(C*H*_3_)_2_), 0.97 (dd, ^3^*J*_H–P_ = 14.7, ^3^*J*_H–H_ = 7.2, 3H, PCH(C*H*_3_)_2_), 0.41 (dd, ^3^*J*_H–P_ = 18.0, ^3^*J*_H–H_ = 7.2, 3H, PCH(C*H*_3_)_2_), −12.57 (dd, ^2^*J*_H–P_ = 36.1, ^2^*J*_H–P_ = 32.4, 1H, OsH). ^13^C{^1^H}-APT NMR (75.48 MHz, CD_2_Cl_2_, 298 K) δ 155.2 (d, ^1^*J*_C–P_ = 31.4, C-arom, POP), 138.3 (d, ^1^*J*_C–P_ = 36.3, C-arom, POP), 136.6, 132.3 (both s, CH-arom), 129.8 (d, ^1^*J*_C–P_ = 35.4, C-arom), 129.3 (s, C-arom), 129.0, 128.8, 127.9, 127.8, 127.5, 127.0, 126.8 (all s, CH-arom), 126.6, 125.9 (both s, C-arom), 125.2, 125.1, 125.1, 124.9 (all s, CH-arom), 123.2, 106.8, 86.0 (all s, C-arom), 80.3 (s, CH-arom), 75.0 (d, ^1^*J*_C–P_ = 12.2, CH-arom), 37.4 (s, *C*(CH_3_)_2_), 34.2 (d, ^1^*J*_C–P_ = 24.2, P*C*H(CH_3_)_2_), 33.6 (d, ^1^*J*_C–P_ = 21.5, P*C*H(CH_3_)_2_), 32.6 (d, ^1^*J*_C–P_ = 23.0, P*C*H(CH_3_)_2_), 30.2 (d, ^1^*J*_C–P_ = 28.0, P*C*H(CH_3_)_2_), 29.1 (s, C(*C*H_3_)_2_), 23.2 (d, ^2^*J*_C–P_ = 7.1, PCH(*C*H_3_)_2_), 22.9–22.6 (m, PCH(*C*H_3_)_2_), 22.5 (d, ^2^*J*_C–P_ = 6.7, PCH(*C*H_3_)_2_), 21.8 (s, C(*C*H_3_)_2_), 21.4–21.2 (m, PCH(*C*H_3_)_2_), 21.0 (d, ^2^*J*_C–P_ = 3.8, PCH(*C*H_3_)_2_). ^31^P{^1^H} NMR (121.49 MHz, CD_2_Cl_2_, 298 K) δ −12.6 (d, ^2^*J*_P–P_ = 11.9), −23.0 (d, ^2^*J*_P–P_ = 11.9). ^19^F{^1^H} NMR (282.38 MHz, CD_2_Cl_2_, 298 K) δ −153.1 (br).

### Preparation of [Os(η^5^-IndPh)H{κ^3^-*P*,*O*,*P*-[xant(P^*i*^Pr_2_)_2_]}][OTf]Cl-*d*_1_(**7**-*d*_1_) Isomers

Two screw-top NMR tubes were charged with **4** (50 mg, 0.058 mmol). Then, 0.4 mL of dichloromethane-*d*_2_ were added to one of them and 0.4 mL of dichloromethane to the other. Subsequently, the solutions were treated with trifluoromethanesulfonic acid-*d* (5.7 μL, 0.064 mmol). After 1 h at room temperature their NMR spectra were recorded. HRMS (electrospray, *m*/*z*) calcd. for C_42_H_51_OOsP_2_ [M – D]^+^: 825.3024, found 825.3043 and calcd. for C_42_H_50_DOOsP_2_ [M – H]^+^: 826.3087, found 826.3078. ^2^H NMR (61.42 MHz, CH_2_Cl_2_, 298 K) δ 6.31 (m), −12.43 (m).

### Protonation of OsCl[C_9_H_6_Ph]{κ^3^-*P*,*O*,*P*-[xant(P^*i*^Pr_2_)_2_]} (**5**) with HBF_4_

Crystals of **5** (20 mg, 0.023 mmol) were introduced into a NMR tube and dissolved in 0.4 mL of dichloromethane-*d*_2_. Then HBF_4_·OEt_2_ was added (3.1 μL, 0.023 mmol). Quantitative and immediate formation of **2** was inferred from the ^1^H and ^31^P{^1^H} NMR spectra of the solution.

### Protonation of OsCl[C_9_H_6_Ph]{κ^3^-*P*,*O*,*P*-[xant(P^*i*^Pr_2_)_2_]} (**5**) with DBF_4_

Two screw-top NMR tubes were charged with crystals of **5** (20 mg, 0.023 mmol). Then, 0.4 mL of dichloromethane-*d*_2_ were added to one of them and 0.4 mL of dichloromethane to the other. Subsequently, both solutions were treated with DBF_4_ (6.3 μL, 0.023 mmol). Quantitative and immediate conversion to **2**-***d***_**1**_ was inferred from the ^1^H and ^2^H NMR spectra of the solutions. HRMS (electrospray, *m*/*z*) calcd. for C_42_H_51_ClDOOsP_2_ [M]^+^: 862.2854, found 862.2838. ^2^H NMR (61.42 MHz, CH_2_Cl_2_, 298 K) δ 7.71 (br).

### Preparation of Os(≡CCH=CPh_2_)(η^2^-HC≡CPh){κ^3^-*P*,*O*,*P*-[xant(P^*i*^Pr_2_)_2_]}]Cl (**8**)

A solution of complex **3** (100 mg, 0.116 mmol) in toluene (5 mL) was treated with phenylacetylene (50 μL, 0.455 mmol), room temperature, for 2 days. After that time, a brown precipitate was formed, which was filtered off, washed with pentane (3 × 2 mL), and dried under vacuum. Brown solid; yield: 65 mg (58%). HRMS (electrospray, *m*/*z*) calcd. for C_50_H_57_OOsP_2_ [M – Cl]^+^: 927.3494, found 927.3464. IR (cm^–1^) ν(C≡C) 1685 (w); ν(C=C) 1541 (m). ^1^H NMR (300.13 MHz, CD_2_Cl_2_, 253 K) δ 8.57 (d, *J*_H–P_ = 9.6, 1H, *H*C≡CPh), 7.62–7.00 (m, 19H, CH-arom), 6.38 (d, ^3^*J*_H–H_ = 6.8, 2H, CH-arom), 5.68 (s, 1H, Os≡C—CH), 3.42 (m, 1H, PC*H*(CH_3_)_2_), 2.99 (m, 1H, PC*H*(CH_3_)_2_), 2.57 (m, 1H, PC*H*(CH_3_)_2_), 2.35 (m, 1H, PC*H*(CH_3_)_2_), 1.76 (s, 3H, C(CH_3_)_2_), 1.46–1.26 (m, 9H, PCH(C*H*_3_)_2_), 1.21–1.00 (m, 9H, PCH(C*H*_3_)_2_), 1.06 (s, 3H, C(CH_3_)_2_), 0.96 (dd, ^3^*J*_H–P_ = 14.5, ^3^*J*_H–H_ = 6.7, 3H, PCH(C*H*_3_)_2_), 0.71 (dd, ^3^*J*_H–P_ = 18.3, ^3^*J*_H–H_ = 6.9, 3H, PCH(C*H*_3_)_2_). ^13^C{^1^H}-APT plus HSQC and HMBC NMR (75.48 MHz, CD_2_Cl_2_, 253 K) δ 265.0 (dd, ^2^*J*_C–P_ = 6.2, ^2^*J*_C–P_ = 4.4, Os≡C), 158.5 (dd, ^4^*J*_C–P_ = 2.2, ^4^*J*_C–P_ = 1.0,=C(Ph)_2_), 155.1 (d, ^2^*J*_C–P_ = 9.1, C-arom, POP), 154.5 (dd, ^2^*J*_C–P_ = 9.1, ^2^*J*_C–P_ = 0.8, C-arom, POP), 140.2 (s, C-ipso, Ph), 140.1 (d, *J*_C–P_ = 2.3, C-ipso, *Ph*-C≡CH), 137.7 (s, C-ipso, Ph), 134.8 (d, ^3^*J*_C–P_ = 4.2, C-arom, POP), 134.1 (d, ^3^*J*_C–P_ = 4.1, C-arom, POP), 133.9 (dd, *J*_C–P_ = 21.6, *J*_C–P_ = 6.6, Ph-*C*≡CH), 131.7 (d, *J*_C–P_ = 1.0, Os≡C-*C*H), 131.6, 130.9, 130.6, 130.3, 129.7, 129.2, 128.8 (all s, CH-arom), 128.7 (d, *J*_C–P_ = 1.6, CH-arom), 128.5, 128.1 (both s, CH-arom), 127.3 (d, *J*_C–P_ = 1.3, CH-arom), 127.0 (s, CH-arom), 126.7 (d, ^2^*J*_C–P_ = 5.9, CH-arom, POP), 126.6 (d, ^2^*J*_C–P_ = 6.0, CH-arom, POP), 122.3 (d, ^1^*J*_C–P_ = 38.0, C-arom, POP), 122.0 (d, ^1^*J*_C–P_ = 39.0, C-arom, POP), 117.7 (resonance inferred from the HSQC spectrum, Ph—C≡*C*H), 36.4 (dd, ^4^*J*_C–P_ = 1.0, ^4^*J*_C–P_ = 1.0, *C*(CH_3_)_2_), 35.5 (d, ^1^*J*_C–P_ = 21.0, P*C*H(CH_3_)_2_), 34.7 (d, ^1^*J*_C–P_ = 23.3, P*C*H(CH_3_)_2_), 32.9 (s, C(*C*H_3_)_2_), 28.0 (d, ^1^*J*_C–P_ = 33.3, P*C*H(CH_3_)_2_), 27.1 (d, ^1^*J*_C–P_ = 32.7, P*C*H(CH_3_)_2_), 23.1 (s, C(*C*H_3_)_2_), 20.6 (s, PCH(*C*H_3_)_2_), 20.4 (d, ^2^*J*_C–P_ = 4.1, PCH(*C*H_3_)_2_), 20.3 (d, ^2^*J*_C–P_ = 6.3, PCH(*C*H_3_)_2_), 20.2 (d, ^2^*J*_C–P_ = 1.5, PCH(*C*H_3_)_2_), 20.1 (d, ^2^*J*_C–P_ = 1.4, PCH(*C*H_3_)_2_), 19.3 (d, ^2^*J*_C–P_ = 1.8, PCH(*C*H_3_)_2_), 19.1 (d, ^2^*J*_C–P_ = 6.7, PCH(*C*H_3_)_2_), 18.5 (d, ^2^*J*_C–P_ = 3.5, PCH(*C*H_3_)_2_). ^31^P{^1^H} NMR (121.49 MHz, CD_2_Cl_2_, 298 K) δ 41.6 ppm (AB spin system, Δν = 53.0 Hz, *J*_A–B_ = 16.9 Hz).

### Preparation of [Os(≡CCH=CPh_2_)(η^2^-HC≡CPh){κ^3^-*P*,*O*,*P*-[xant(P^*i*^Pr_2_)_2_]}]BF_4_

A mixture of **8** (50 mg, 0.052 mmol) and NaBF_4_ (0.034 mg, 0.031 mmol) in 7 mL of acetone was stirred for 3 h. After this time, the solvent was removed under a vacuum and 8 mL of dichloromethane were added. The suspension was filtered through Celite and the resulting solution was concentrated until dryness. The residue was washed with diethyl ether (5 × 8 mL) and vacuum-dried. Crystals of **8**-**BF**_**4**_ suitable for X-ray diffraction analysis were obtained by slow diffusion of pentane into a solution of the solid in dichloromethane. Yield: 46 mg (88%). Anal. Calcd for C_50_H_57_BF_4_OOsP_2_: C, 59.29; H, 5.67. Found: C, 59.16; H, 5.79. The ^1^H and ^31^P{^1^H} NMR data were identical with those reported for the Cl-salt.

### Preparation of [Os(≡CCH=CPh_2_)(DC≡CPh){xant(P^*i*^Pr_2_)_2_}]Cl (**8**-*d*_1_)

This compound was synthesized following the procedure described for compound **8** but using phenylacetylene-*d* instead of phenylacetylene. Brown solid, yield: 72 mg (64%). HRMS (electrospray, *m*/*z*) calcd. for C_50_H_56_DOOsP_2_ [M – Cl]^+^: 928.3557, found 928.3563. ^2^H NMR (61.42 MHz, CH_2_Cl_2_, 298 K) δ 8.71 (br).

## Supporting Information Available

Experimental details, crystallographic data, NMR spectra, and theoretical studies (PDF)
Cartesian coordinates of the optimized structures (XYZ)

## Supplementary Material

om1c00176_si_001.pdf

om1c00176_si_002.xyz
