# Supplementary material for: Repercussion of a 1,3-Hydrogen Shift in a Hydride-Osmium-Allenylidene Complex
Source: Organometallics. 2021 May 12;40(10):1523–37. doi: 10.1021/acs.organomet.1c00176 (PMC9180373; doi:10.1021/acs.organomet.1c00176)
Supplement: Supplementary file 1 — om1c00176_si_001.pdf [file om1c00176_si_001.pdf]

SUPPORTING INFORMATION

Repercussion of a 1,3-Hydrogen Shift in a Hydride-Osmium-Allenylidene Complex

Miguel A. Esteruelas,* Enrique Oñate, Sonia Paz, and Andrea Vélez

Departamento de Química Inorgánica – Instituto de Síntesis Química y Catálisis Homogénea (ISQCH) – Centro de Innovación en Química Avanzada (ORFEO-CINQA), Universidad de Zaragoza – CSIC, 50009 Zaragoza, Spain

* e-mail: maester@unizar.es

Contents:

– Experimental Details S2

– Structural Analysis of Complexes **2**, **3**, **4**, **5**, **7**, and **8**. S3

– NMR Spectra S6

– Computational Details S23

– DFT computed energy profiles S24

– Energies of Optimized Structures S26

– References S33

– Experimental Details

General Information. All reactions were carried out with exclusion of air using Schlenk-tube techniques or in a drybox. Pentane, dichloromethane, diethyl ether, acetonitrile, and toluene were obtained oxygen- and water-free from an MBraun solvent purification apparatus, while methanol, 2-propanol, tetrahidrofuran (THF), acetone, 1,2-dichloroethane, and fluorobenzene were dried and distilled under argon prior to use. ^{1}H, ^{13}C{^{1}H}, and ^{31}P{^{1}H}, ^{2}H, and ^{19}F NMR spectra were recorded on Bruker 300 ARX, Bruker Avance 300 MHz or Bruker Avance 400 MHz. Chemical shifts (expressed in ppm) are referenced to residual solvent peaks (^{1}H, ^{13}C{^{1}H}, ^{2}H), external 85% H_3PO_4 (^{31}P{^{1}H}), or $CFCl_3$ (^{19}F). Coupling constants J and N ($N = J_{P\text{-}H} + J_{P'\text{-}H}$ for ^{1}H and $N = J_{P\text{-}C} + J_{P'\text{-}C}$ for ^{13}C{^{1}H}) are given in hertz. Attenuated total reflection infrared spectra (ATR-IR) of solid samples were run on a PerkinElmer Spectrum 100 FT-IR spectrometer. Elemental analyses were carried out in a PerkinElmer 2400 CHNS/O analyzer. High-resolution electrospray mass spectra and CSS measurements were acquired using a MicroTOF-Q and a timsTOF hybrid quadrupole time-of-flight spectrometers (Bruker Daltonics, Bremen, Germany). [(Os(H···H){κ^3-*P,O,P*-[xant(P^{i}Pr$_2$)$_2$]})$_2$(μ-Cl)$_2$][BF_4]$_2$ (**1**) was prepared as reported previously.[1]

– Structural Analysis of Complexes 2, 3, 4, 5, 7, and 8.

X-ray data were collected for the complexes on a Bruker Smart APEX or Bruker Apex DUO diffractometers equipped with a normal focus, and 2.4 kW sealed tube source (Mo radiation, λ = 0.71073 Å). Data were collected over the complete sphere covering 0.3° in ω. Data were corrected for absorption by using a multiscan method applied with the SADABS program.[2] The structures were solved by Patterson or direct methods and refined by full-matrix least squares on F^2 with SHELXL2016,[3] including isotropic and subsequently anisotropic displacement parameters. The hydrogen atoms were observed in the last Fourier Maps or calculated, and refined freely or using a restricted riding model. The hydride ligands were located in the last Fourier Maps and refined with restrained distance to osmium atoms.

Complex **8** was observed disordered 80:20 about a virtual bisecting plane containing the osmium atom and carbyne ligand that divides the POP ligand in two. The disordered groups were refined with restrained geometry, isotropic thermal parameters and complementary occupancy factors.

Crystal data for **2** (CCDC 2067293): $C_{42}H_{52}ClOOsP_2$, BF_4, M_W 947.23, red, irregular block (0.204 x 0.173 x 0.055 mm^3), monoclinic, space group $P2_1/c$, *a*: 12.9131(12) Å, *b*: 21.345(2) Å, *c*: 14.3459(13) Å, β: 90.3440(10)°, V = 3954.1(6) Å^3, Z = 4, Z' = 1, D_{calc}: 1.591 g cm^{-3}, F(000): 1904, T = 100(2) K, μ 3.425 mm^{-1}. 62891 measured reflections (2θ: 3-57°, ω scans 0.3°), 10757 unique (R_{int} = 0.0315); min./max. transm. factors 0.676/0.862. Final agreement factors were R^1 = 0.0224 (9555 observed reflections, $I > 2\sigma(I)$) and wR^2 = 0.0555; data/restraints/parameters 10757/1/485; GoF = 1.035. Largest peak and hole: 2.041 (close to osmium atoms) and -0.454 e/ Å^3.

Crystal data for **3** (CCDC 2067294): $C_{42}H_{51}ClOOsP_2$, 0.25(C_5H_{12}), M_W 877.45, black, irregular block (0.300 x 0.113 x 0.103 mm^3), tetragonal, space group $I4_1/a$, *a*: 31.078(3) Å, *b*: 31.078(3) Å, *c*: 16.8061(15) Å, *V* = 16232(3) Å^3, *Z* = 16, *Z'* = 1, D_{calc}: 1.436 g cm^{-3}, F(000): 7112, T = 100(2) K, μ 3.318 mm^{-1}. 128250 measured reflections (2θ: 3-57°, ω scans 0.3°), 11258 unique (R_{int} = 0.0840); min./max. transm. factors 0.660/0.862. Final agreement factors were R^1 = 0.0462 (7416 observed reflections, I > 2σ(I)) and wR^2 = 0.1361; data/restraints/parameters 11258/13/446; GoF = 1.040. Largest peak and hole: 3.635 (close to osmium atoms) and -1.045 e/ Å^3.

Crystal data for **4** (CCDC 2067295): $C_{42}H_{51}ClOOsP_2$, M_W 859.41, red, irregular block (0.301 x 0.163 x 0.042 mm^3), monoclinic, space group $P2_1/n$, *a*: 11.5230(6) Å, *b*: 17.2484(9) Å, *c*: 19.0100(10) Å, *β*: 98.1410(10)°, *V* = 3740.2(3) Å^3, *Z* = 4, *Z'* = 1, D_{calc}: 1.526 g cm^{-3}, F(000): 1736, T = 100(2) K, μ 3.598 mm^{-1}. 8955 measured reflections (2θ: 3-57°, ω scans 0.3°), 7030 unique (R_{int} = 0.0513); min./max. transm. factors 0.639/0.862. Final agreement factors were R^1 = 0.0297 (7030 observed reflections, I > 2σ(I)) and wR^2 = 0.0707; data/restraints/parameters 8955/1/440; GoF = 1.038. Largest peak and hole: 1.331 (close to osmium atoms) and -1.440 e/ Å^3.

Crystal data for **5** (CCDC 2067296): $C_{42}H_{51}ClOOsP_2$, CH_4O, M_W 891.46, green, irregular block (0.272 x 0.158 x 0.025 mm^3), orthorhombic, space group $Pca2_1$, *a*: 15.7790(17) Å, *b*: 17.2147(18) Å, *c*: 14.1813(15) Å, *V* = 3852.1(7) Å^3, *Z* = 4, *Z'* = 1, D_{calc}: 1.537 g cm^{-3}, F(000): 1808, T = 100(2) K, μ 3.499 mm^{-1}. 37412 measured reflections (2θ: 3-57°, ω scans 0.3°), 9162 unique (R_{int} = 0.0952); min./max. transm. factors 0.6709/0.862. Final agreement factors were R^1 = 0.0603 (6953 observed reflections, I > 2σ(I)) and wR^2 = 0.1325; Flack parameter -0.008(17); data/restraints/parameters 9162/20/457; GoF = 1.063. Largest peak and hole: 1.732 (close to osmium atoms) and -2.903 e/ Å^3.

Crystal data for **7** (CCDC 2067297): $C_{42}H_{52}OOsP_2$, $2(BF_4)$, $1.7(C_2H_4Cl_2)$, M_W 1166.81, yellow, irregular block (0.300 x 0.081 x 0.052 mm^3), monoclinic, space group $P2_1/c$, *a*: 11.5720(16) Å, *b*: 28.274(4) Å, *c*: 15.423(2) Å, *β*: 94.207(2)°, *V* = 5032.5(12) $Å^3$, *Z* = 4, *Z'* = 1, D_{calc}: 1.540g cm^{-3}, F(000): 2340, T = 120(2) K, μ 2.841 mm^{-1}. 58444 measured reflections (2θ: 3-57°, ω scans 0.3°), 14551 unique (R_{int} = 0.0972); min./max. transm. factors 0.568/0.862. Final agreement factors were R^1 = 0.0731 (9482 observed reflections, I > 2σ(I)) and wR^2 = 0.1845; data/restraints/parameters 14551/23/557; GoF = 1.096. Largest peak and hole: 2.788 (close to osmium atoms) and -2.265 e/ $Å^3$.

Crystal data for **8** (CCDC 2067298): $C_{50}H_{57}OOsP_2$, BF_4, $0.5(C_5H_{12})$, $1.4(CH_2Cl_2)$, H_2O, M_W 1185.89, orange, irregular block (0.168 x 0.154 x 0.131 mm^3), triclinic, space group P-1, *a*: 13.5493(11) Å, *b*: 13.7777(12) Å, *c*: 16.4316(14) Å, *α*: 67.7460(10)°, *β*: 74.4620(10)°, *γ*: 86.5410(10)°, *V* = 2732.3(4)) $Å^3$, *Z* = 2, *Z'* = 1, D_{calc}: 1.441 g cm^{-3}, F(000): 1204, T = 100(2) K, μ 2.581 mm^{-1}. 12603 measured reflections (2θ: 3-57°, ω scans 0.3°), 9981 unique (R_{int} = 0.0472); min./max. transm. factors 0.698/0.862. Final agreement factors were R^1 = 0.0788 (9981 observed reflections, I > 2σ(I)) and wR^2 = 0.2066; data/restraints/parameters 12603/42/627; GoF = 1.108. Largest peak and hole: 3.092 (close to osmium atoms) and -3.225 e/ $Å^3$.

– NMR Spectra

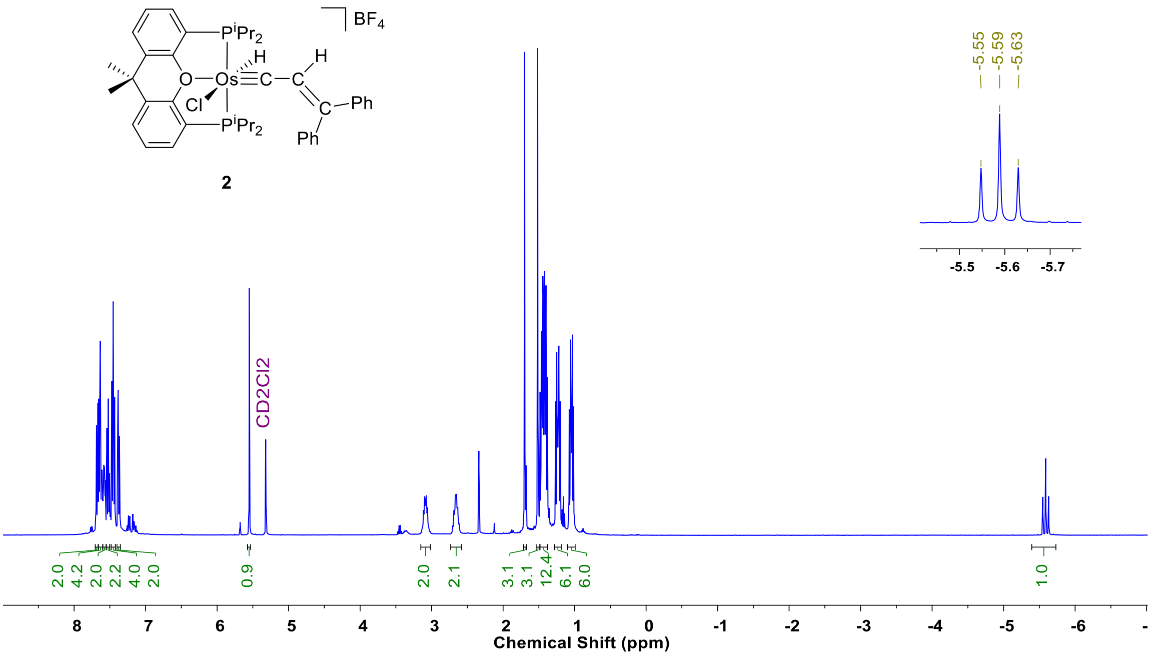

Figure S1. ^{1}H NMR spectrum (400.16 MHz, CD_2Cl_2, 298 K) of compound **2**.

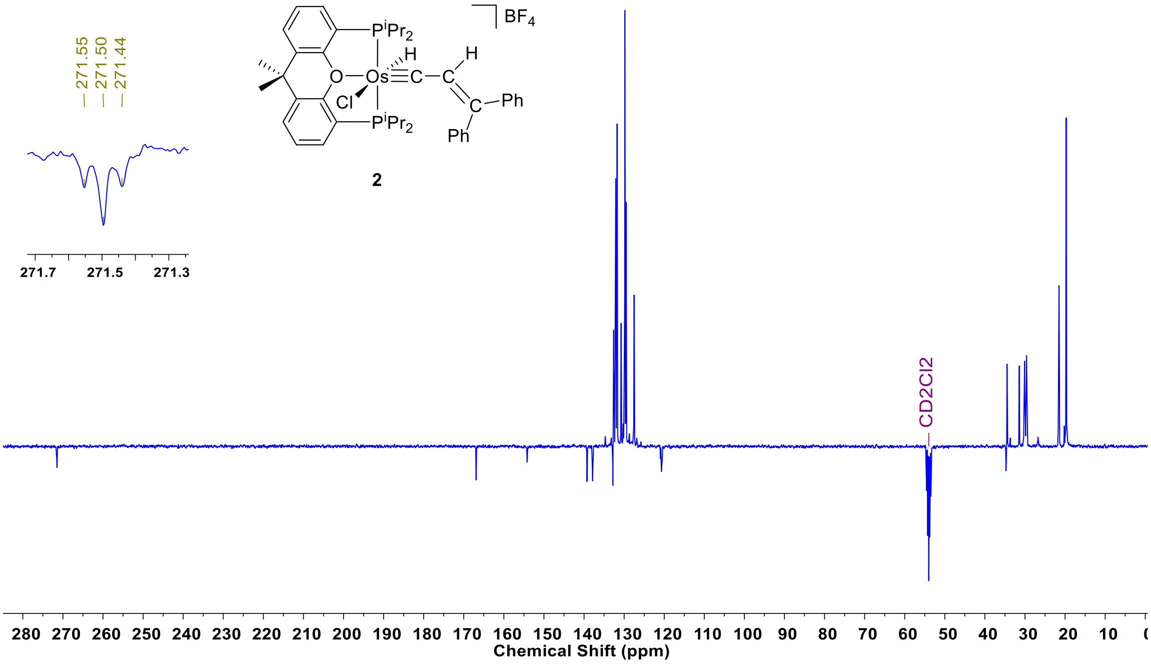

Figure S2. $^{13}C\{^{1}H\}$-APT NMR spectrum (100.64 MHz, CD_2Cl_2, 298 K) of compound **2**.

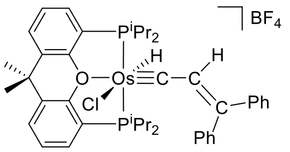

2

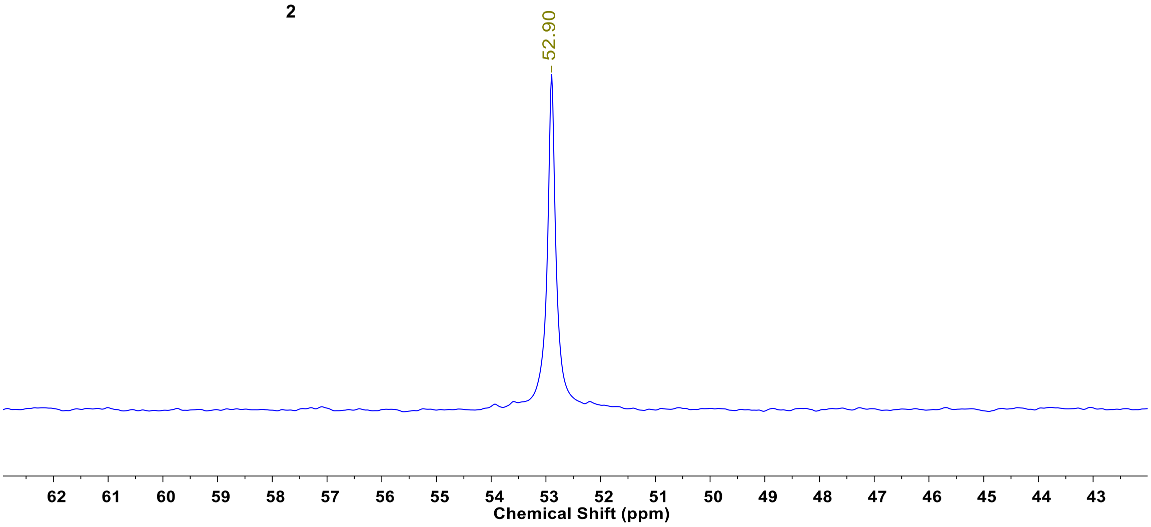

Figure S3. $^{31}P\{^{1}H\}$ NMR spectrum (121.49 MHz, CD_2Cl_2, 298 K) of compound **2**.

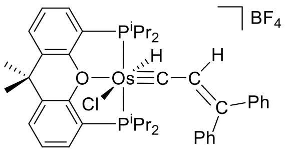

2

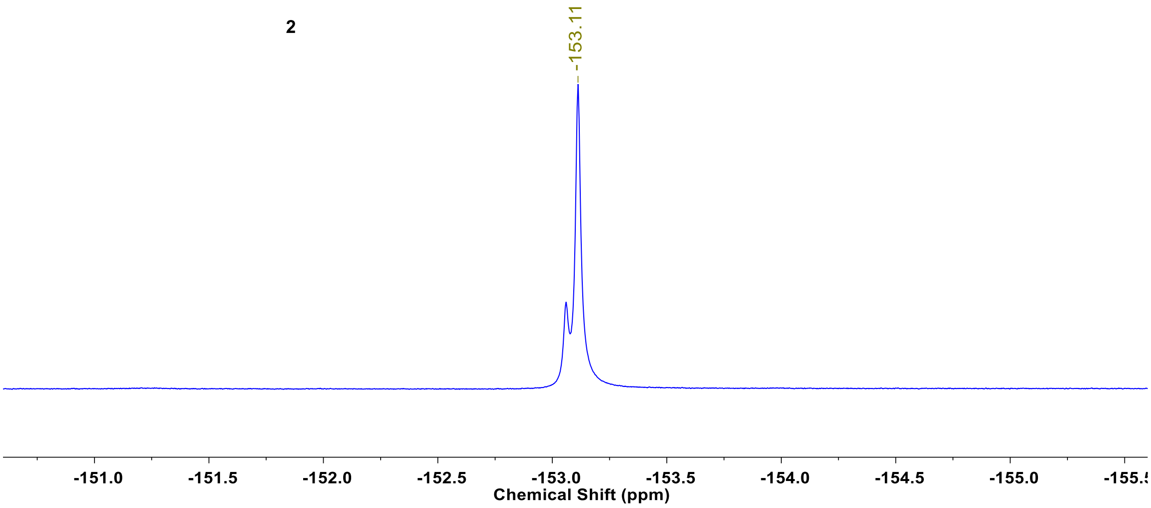

Figure S4. $^{19}F\{^{1}H\}$ NMR spectrum (376.49 MHz, CD_2Cl_2, 298 K) of compound **2**.

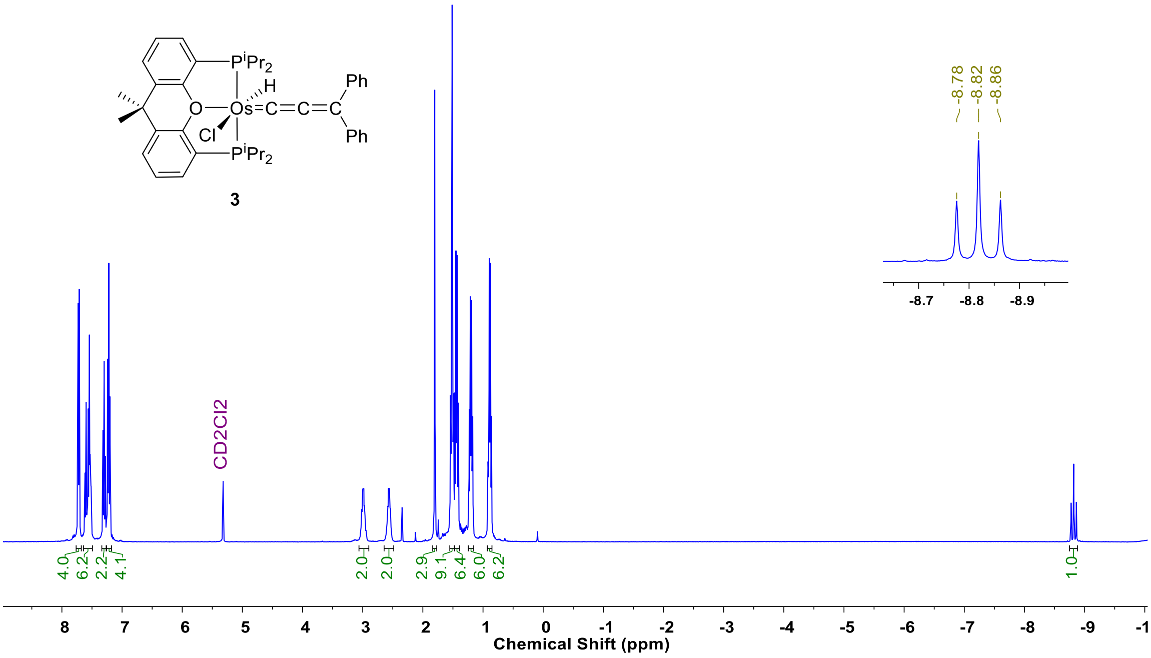

Figure S5. ^{1}H NMR spectrum (400.13 MHz, CD_2Cl_2, 298 K) of compound **3.**

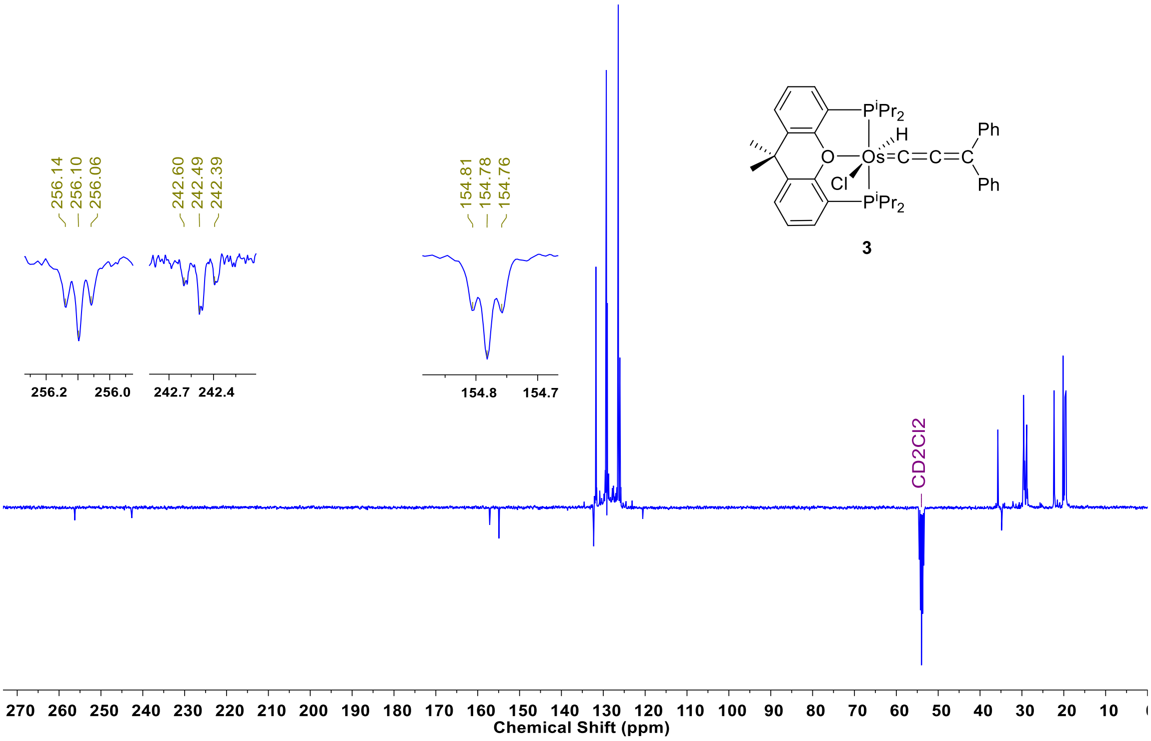

Figure S6. $^{13}C\{^{1}H\}$-APT NMR spectrum (100.64 MHz, CD_2Cl_2, 298 K) of compound **3**.

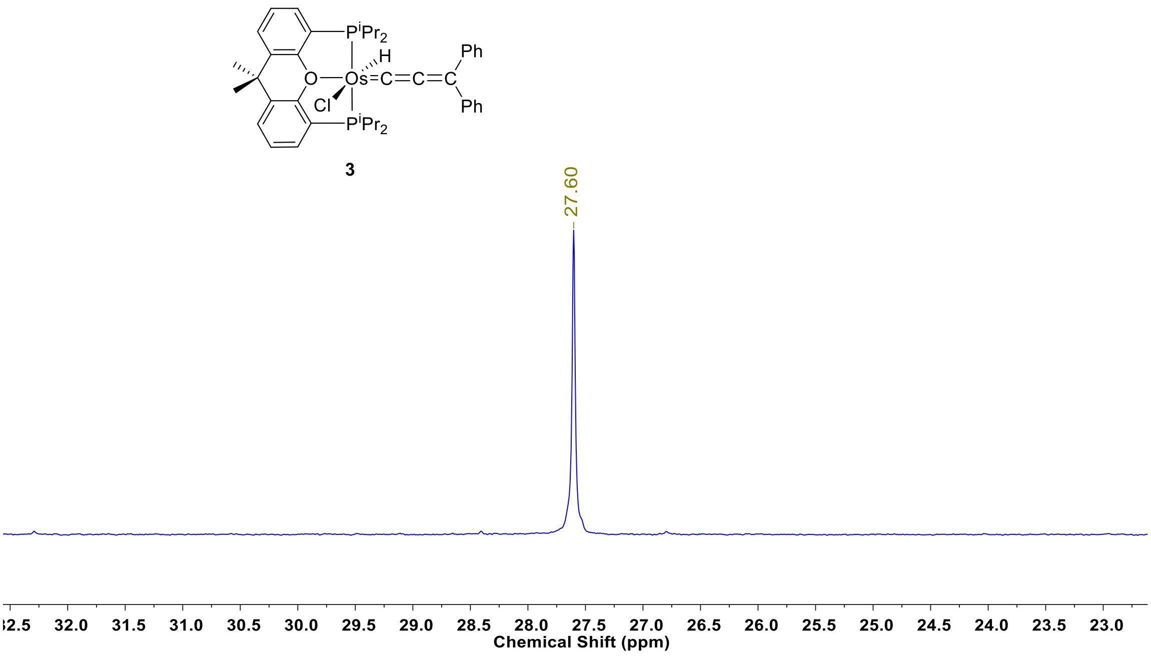

Figure S7. $^{31}P\{^{1}H\}$ NMR spectrum (121.49 MHz, CD_2Cl_2, 298 K) of compound **3**.

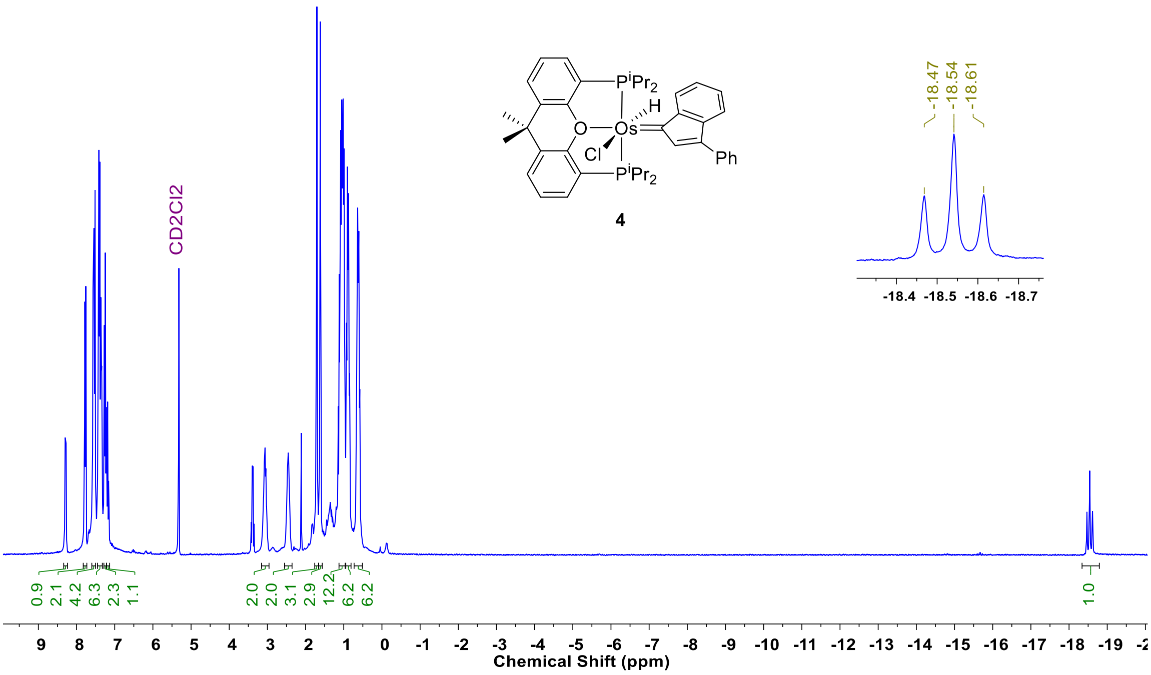

Figure S8. ^{1}H NMR spectrum (300.13 MHz, CD_2Cl_2, 223 K) of compound **4**.

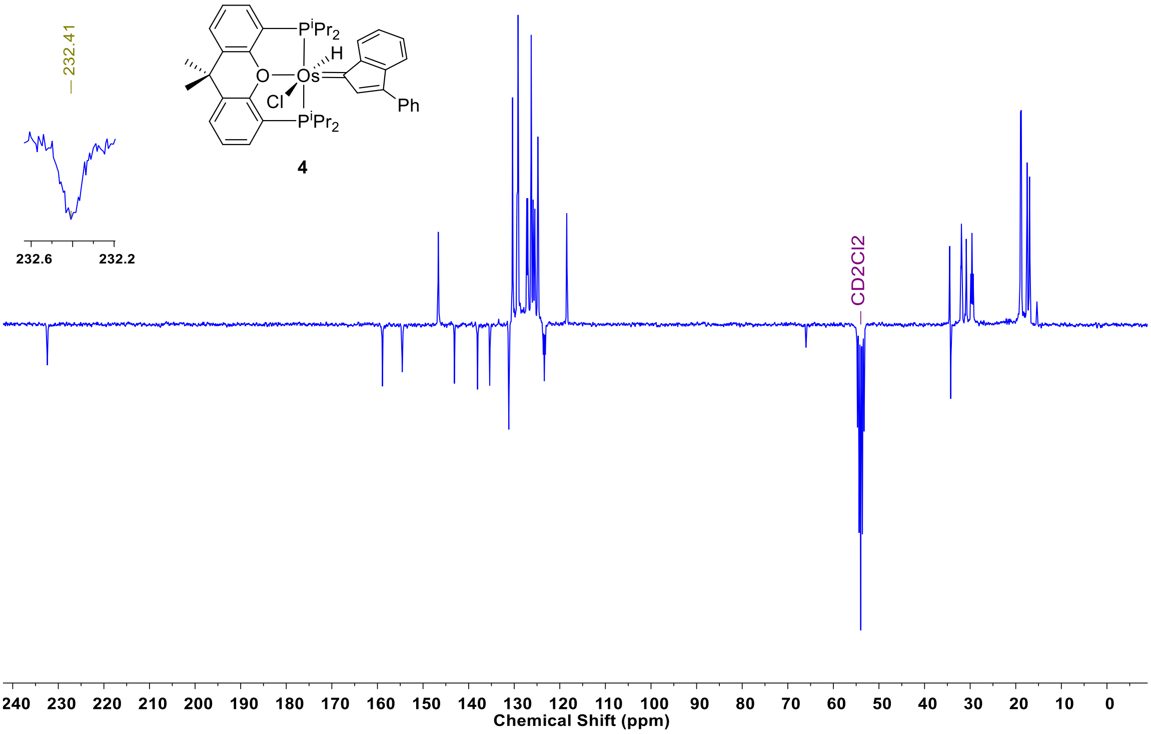

Figure S9. $^{13}C\{^1H\}$-APT NMR spectrum (75.48 MHz, CD_2Cl_2, 223 K) of compound **4**.

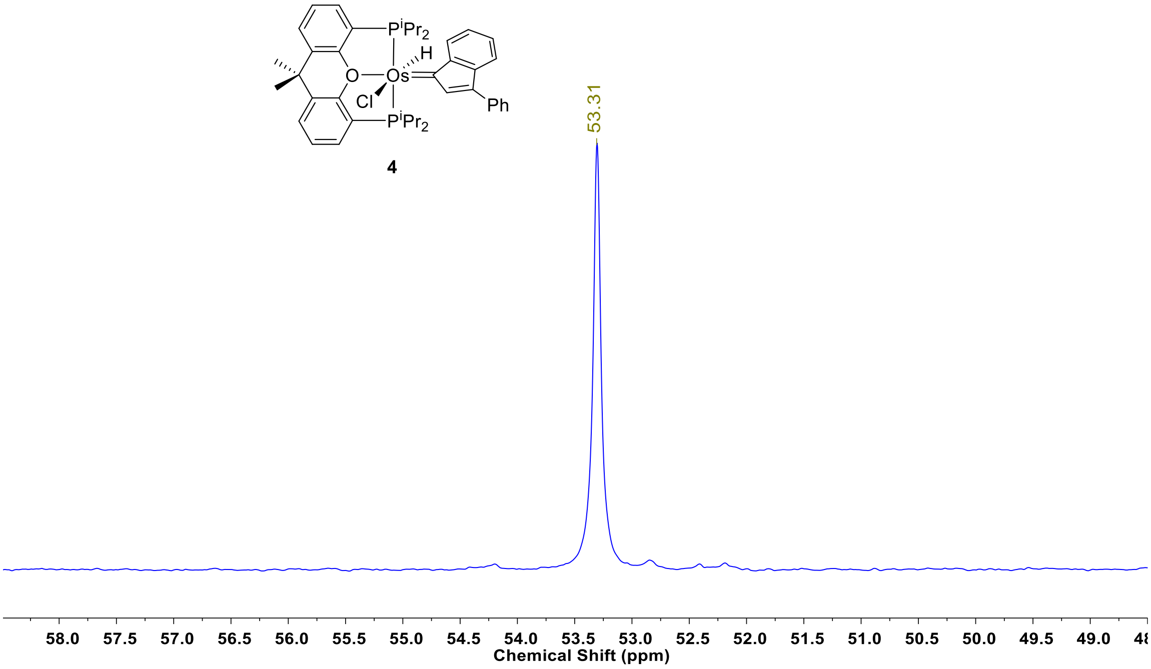

Figure S10. $^{31}P\{^1H\}$ NMR spectrum (121.50 MHz, CD_2Cl_2, 223 K) of compound **4**.

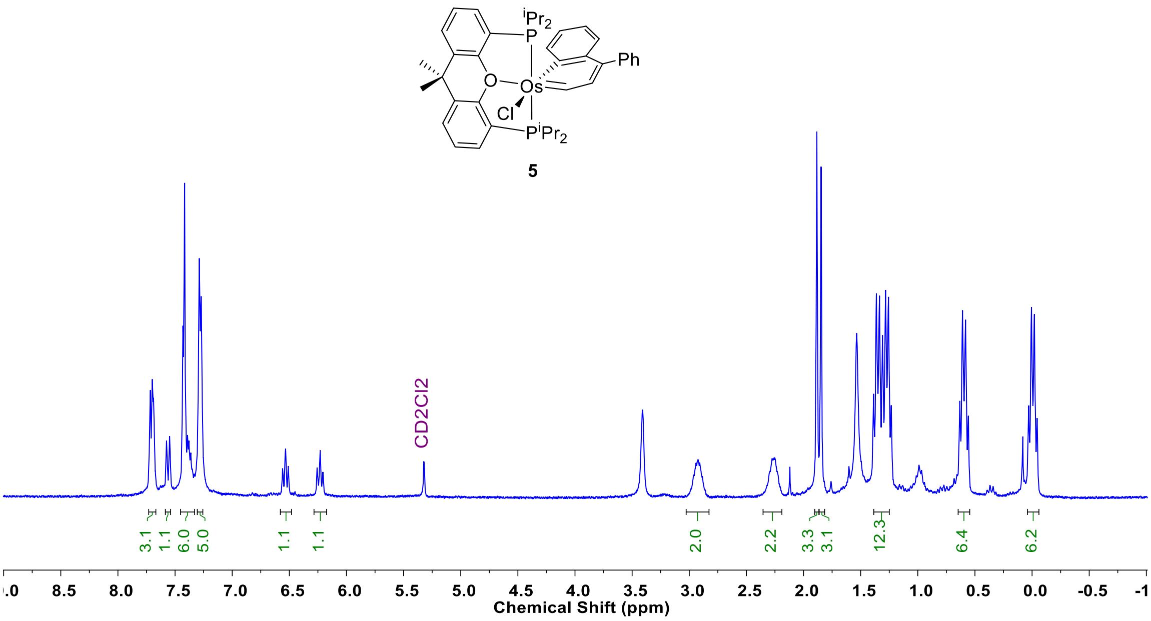

Figure S11. ^{1}H NMR spectrum (300.13 MHz, CD_2Cl_2, 298 K) of compound **5**.

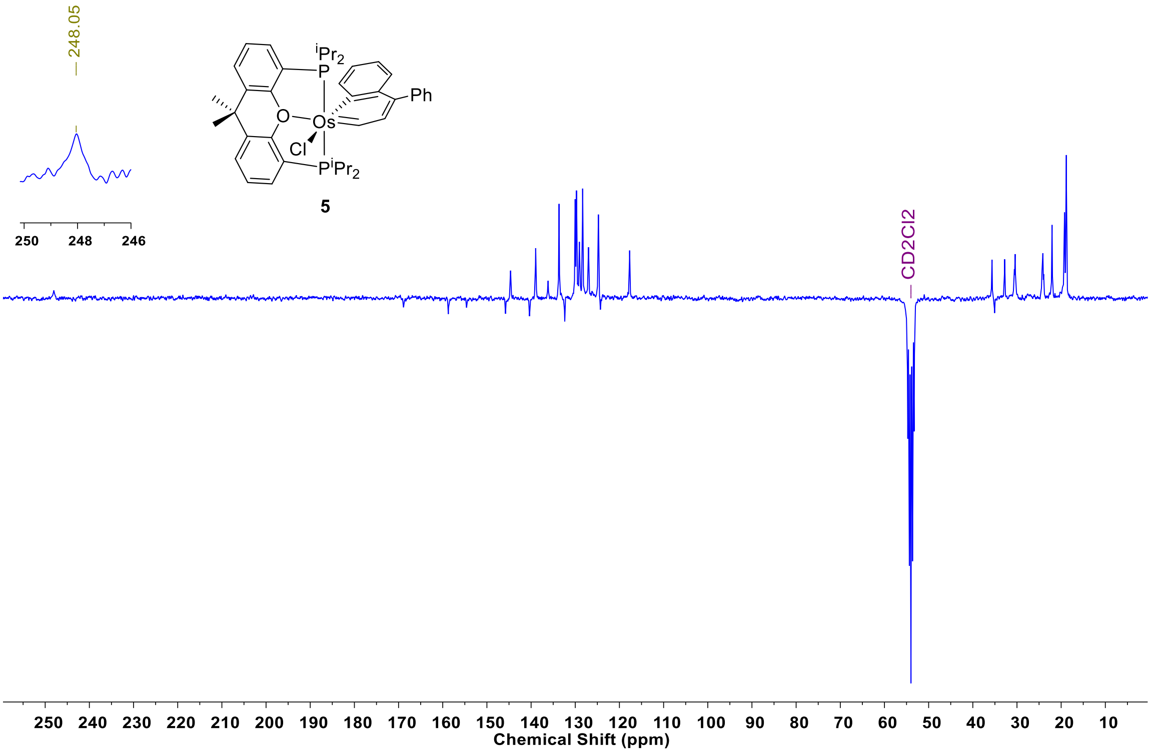

Figure S12. $^{13}C\{^{1}H\}$-APT NMR spectrum (75.48 MHz, CD_2Cl_2, 298 K) of compound **5**.

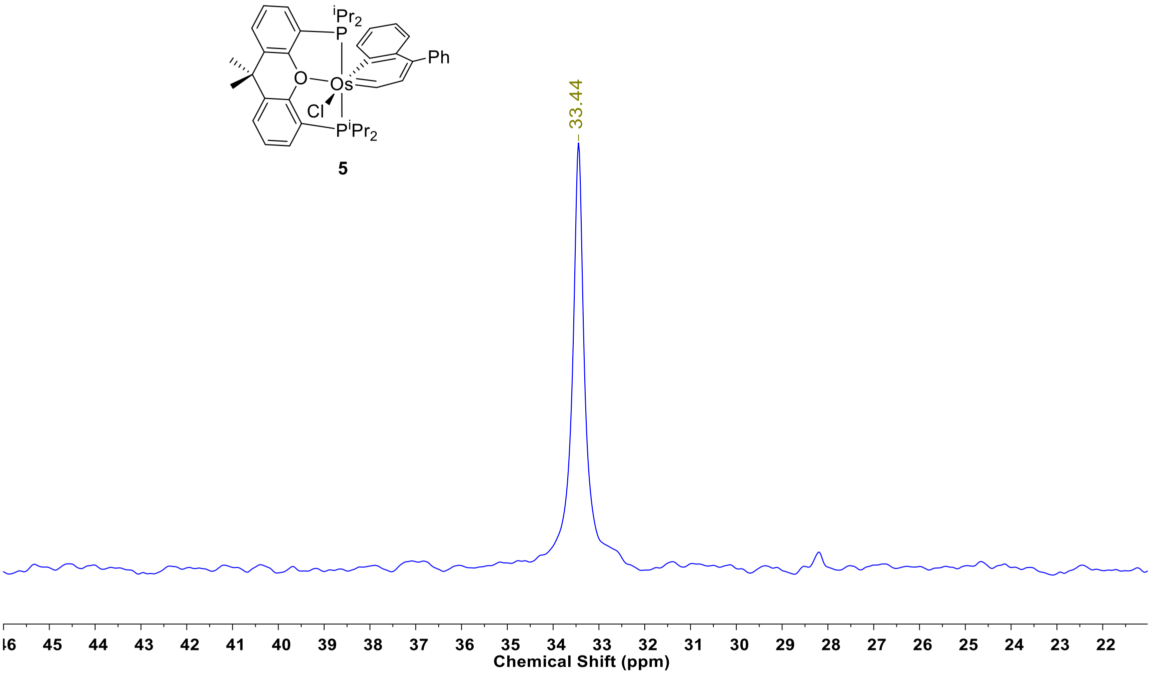

Figure S13. $^{31}P\{^{1}H\}$ NMR spectrum (121.50 MHz, CD_2Cl_2, 298 K) of compound **5**.

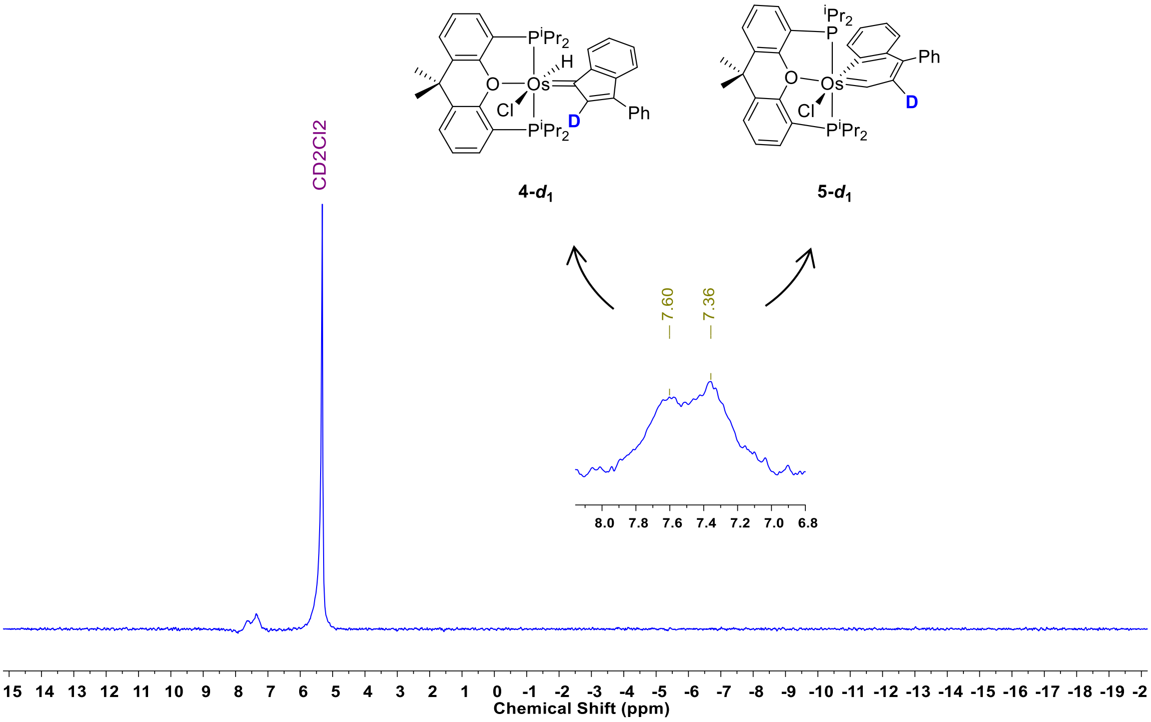

Figure S14. ^{2}H NMR spectrum (61.42 MHz, CH_2Cl_2, 298 K) of compounds **4-*d*$_1$** and **5-*d*$_1$**.

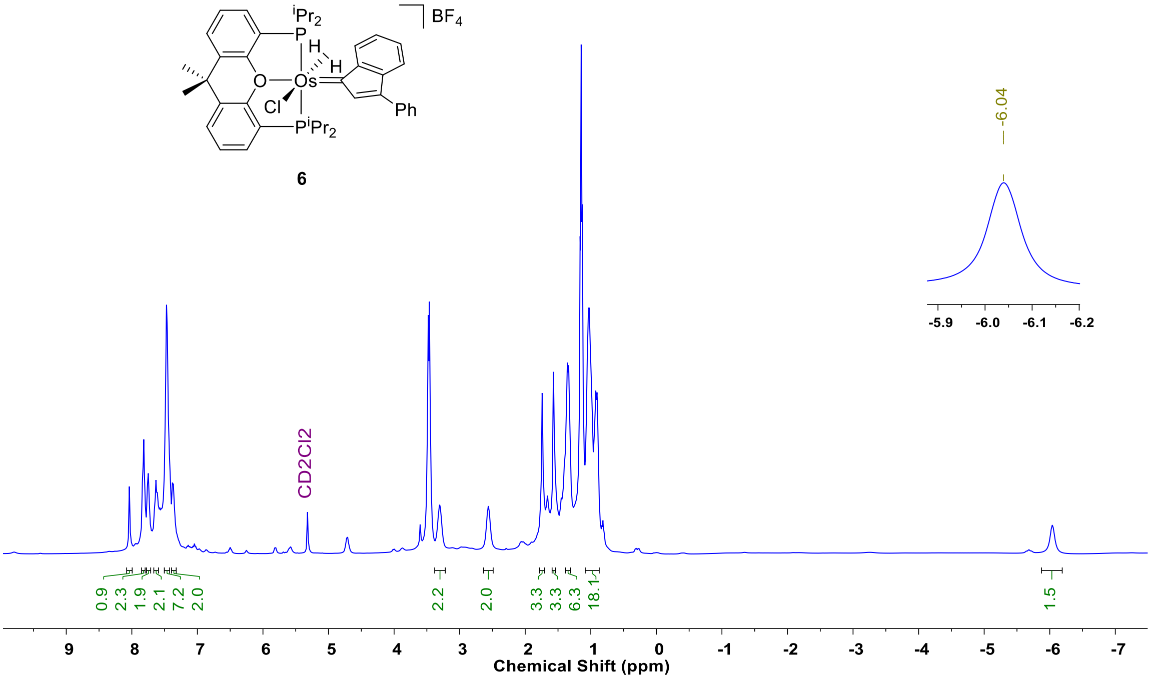

Figure S15. ^{1}H NMR spectrum (400.13 MHz, CD_2Cl_2, 223 K) of compound **6**.

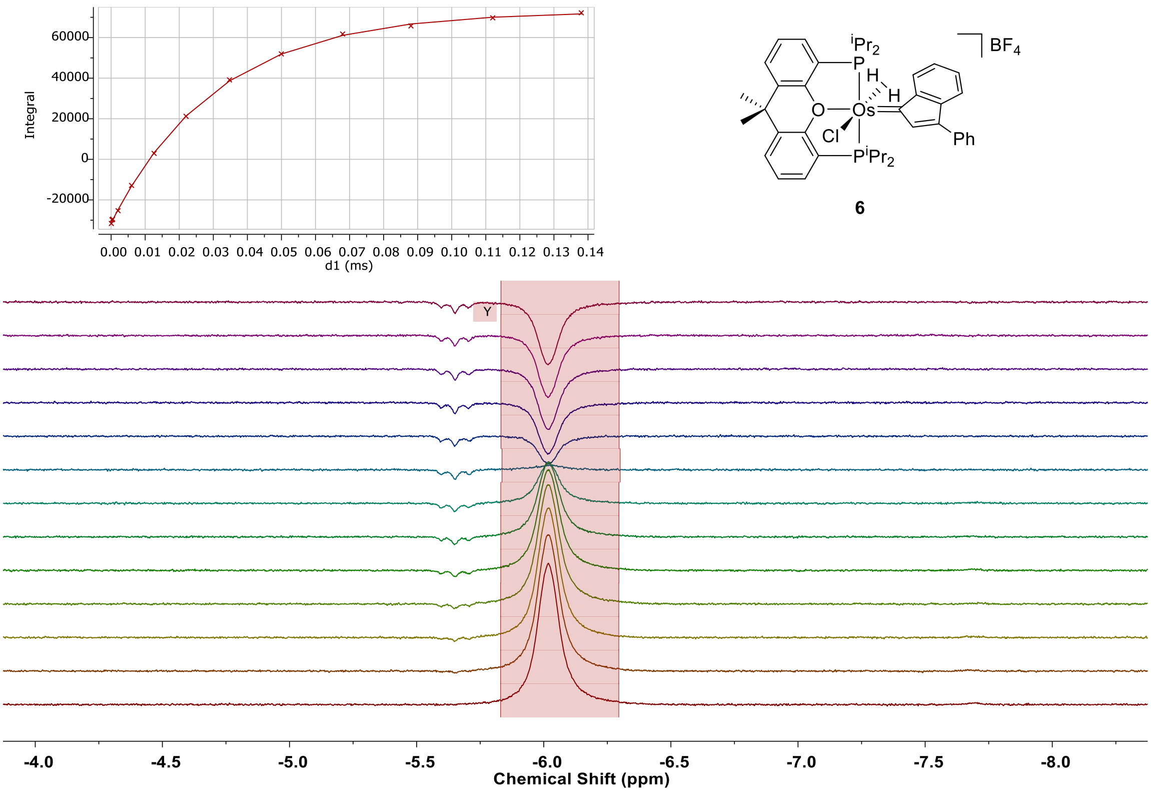

Figure S16. ^{1}H NMR T_1(min) relaxation time measurement (400.13 MHz, CD_2Cl_2, 217 K, OsH) of compound **6**.

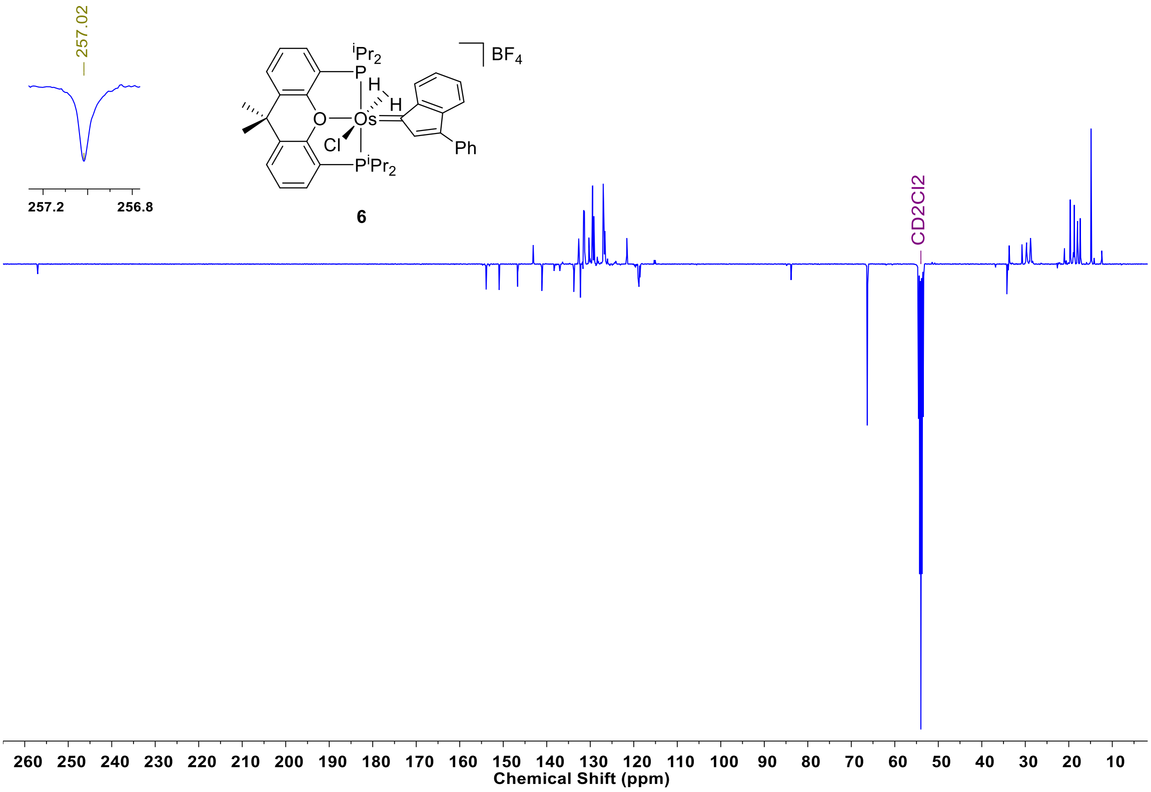

Figure S17. $^{13}C\{^{1}H\}$-APT NMR spectrum (100.63 MHz, CD_2Cl_2, 223 K) of compound **6**.

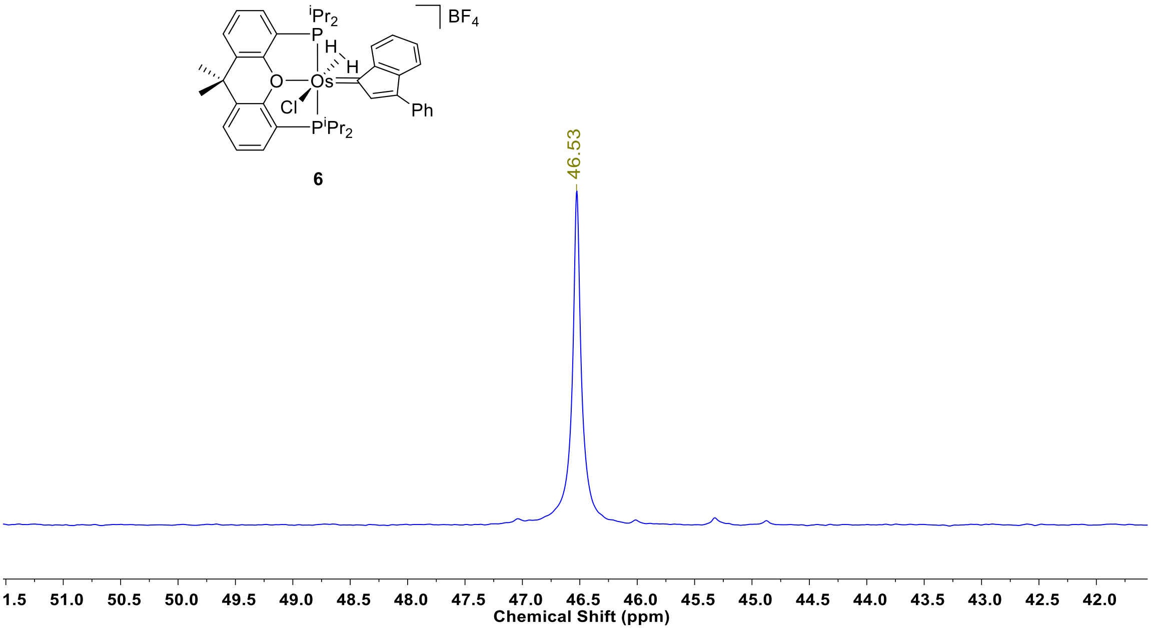

Figure S18. $^{31}P\{^{1}H\}$ NMR spectrum (161.98 MHz, CD_2Cl_2, 223 K) of compound **6**.

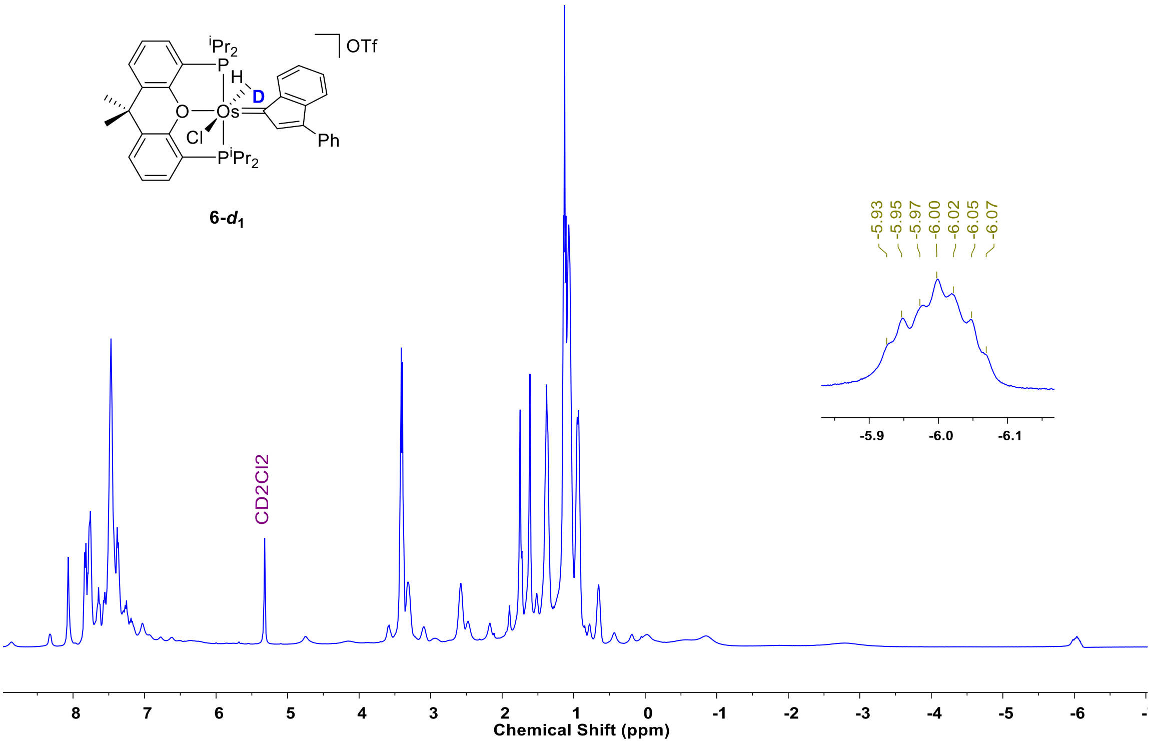

Figure S19. ^{1}H NMR spectrum (400.13 MHz, CD_2Cl_2, 253 K) of compound **6-*d*₁**.

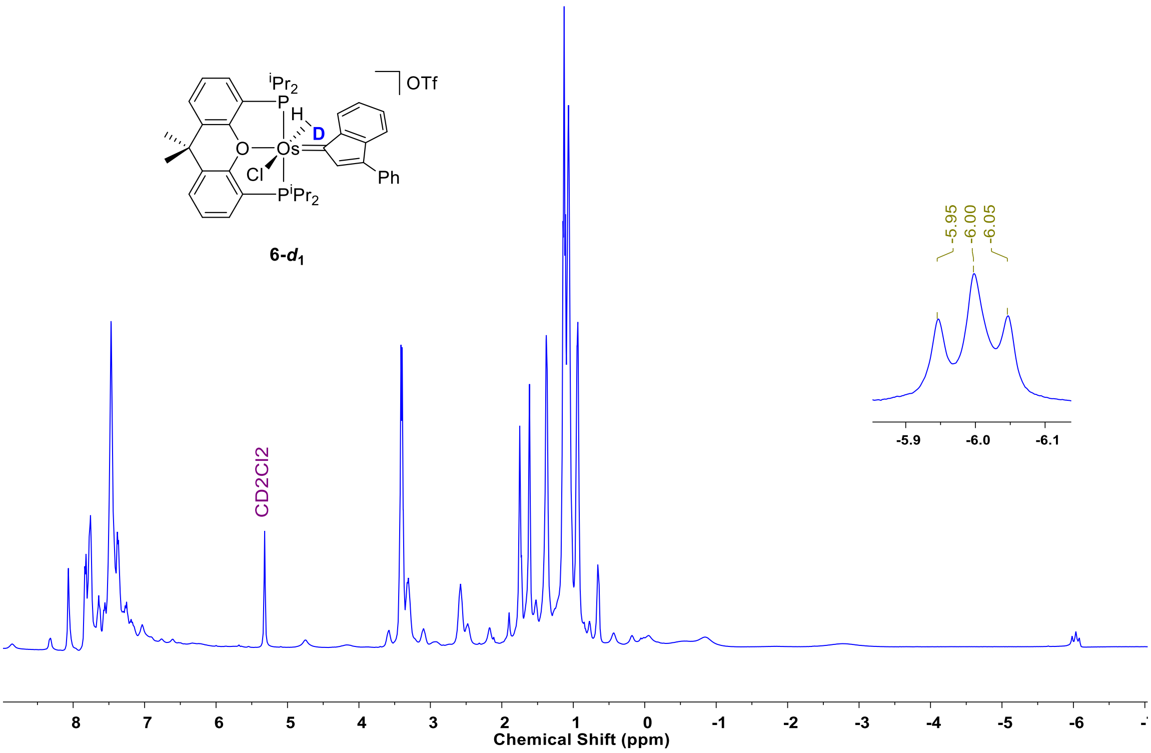

Figure S20. $^{1}H\{^{31}P\}$ NMR spectrum (400.13 MHz, CD_2Cl_2, 253 K) of compound **6-d_1**.

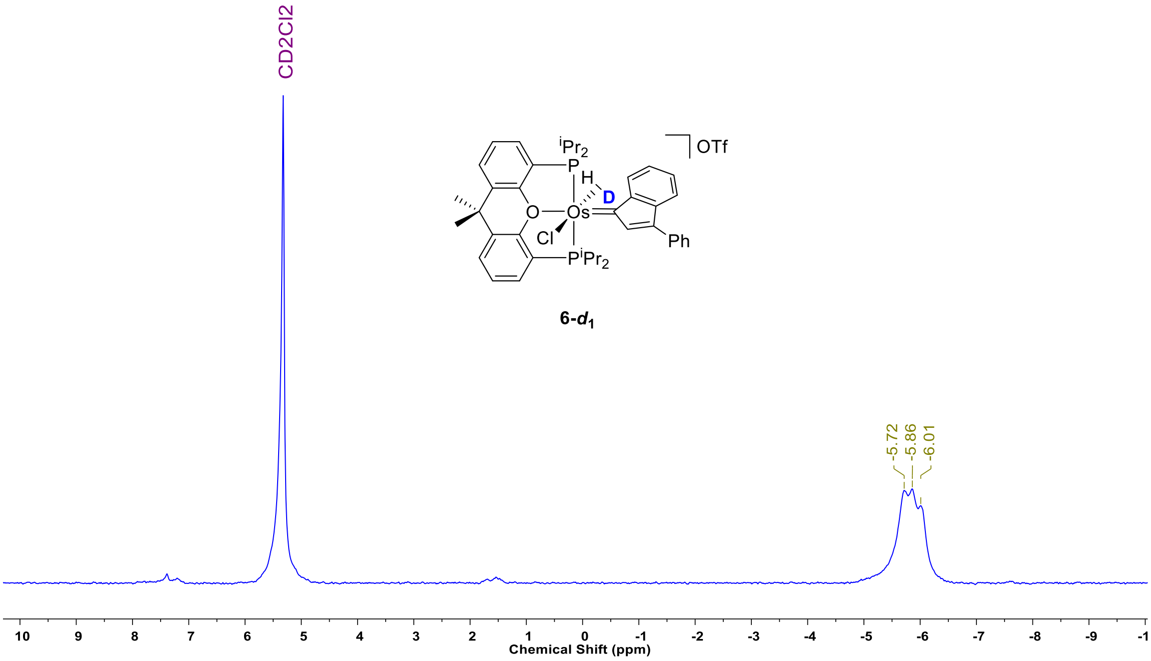

Figure S21. ^{2}H NMR spectrum (61.42 MHz, CH_2Cl_2, 223 K) of compound **6-*d*$_1$**.

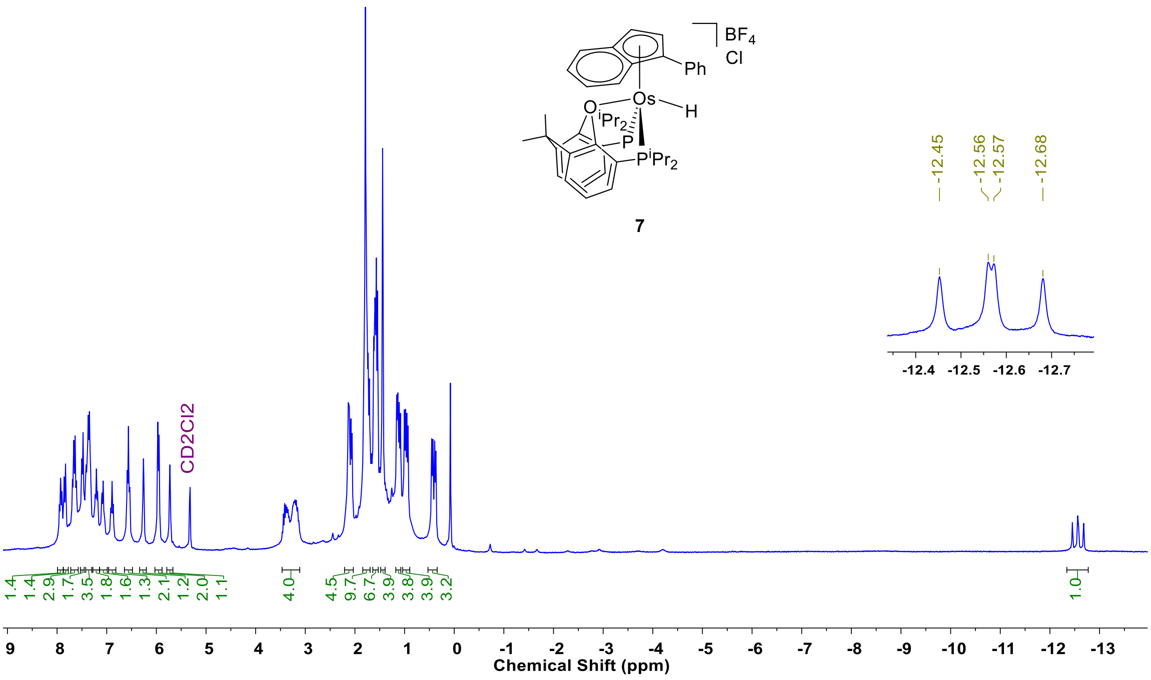

Figure S22. ^{1}H NMR spectrum (300.13 MHz, CD_2Cl_2, 298 K) of compound **7**.

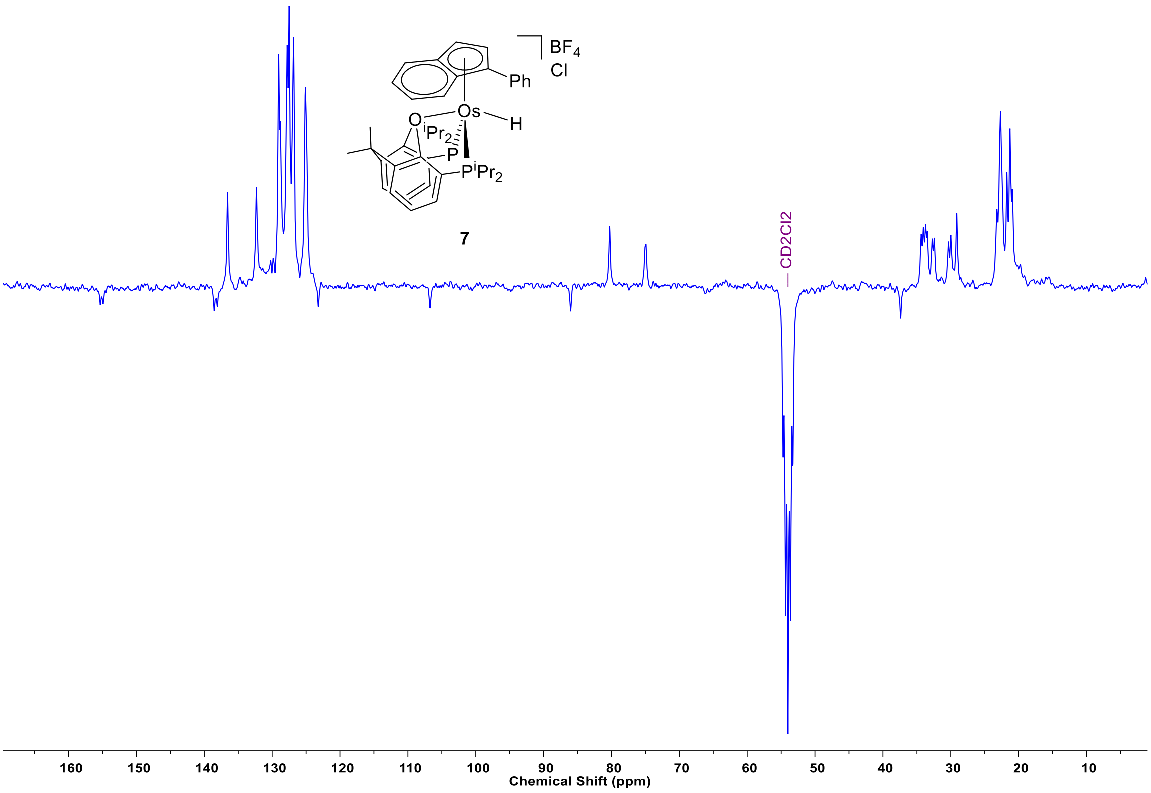

Figure S23. $^{13}C\{^{1}H\}$-APT NMR spectrum (75.48 MHz, CD_2Cl_2, 298 K) of compound **7**.

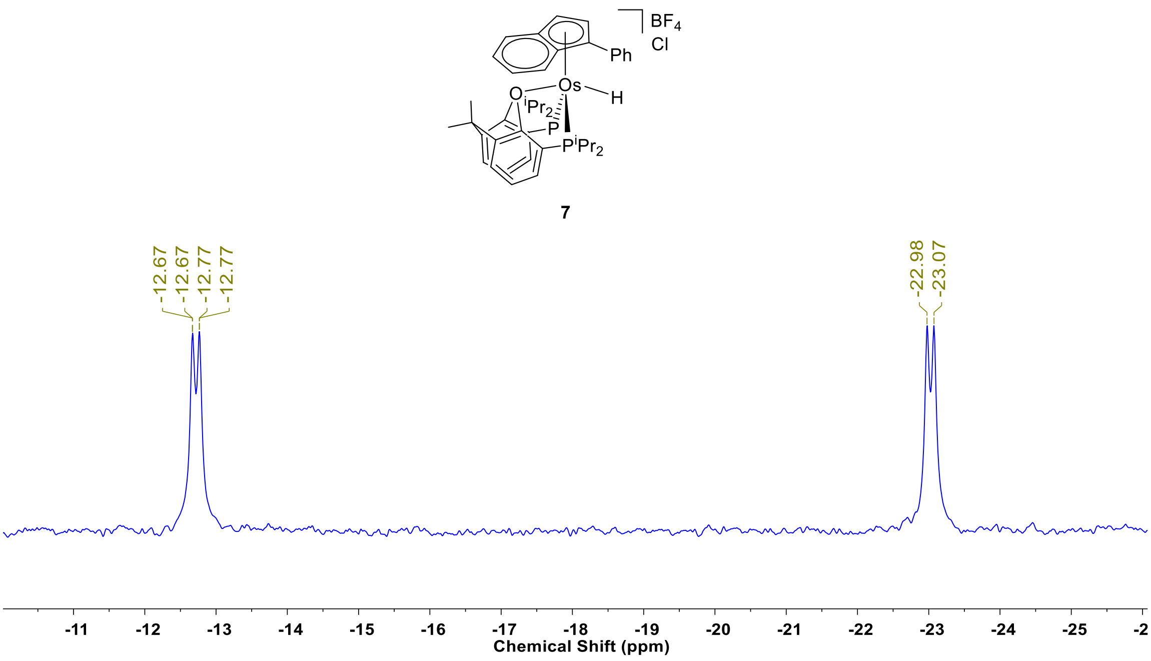

Figure S24. $^{31}P\{^{1}H\}$ NMR spectrum (121.49 MHz, CD_2Cl_2, 298 K) of compound **7**.

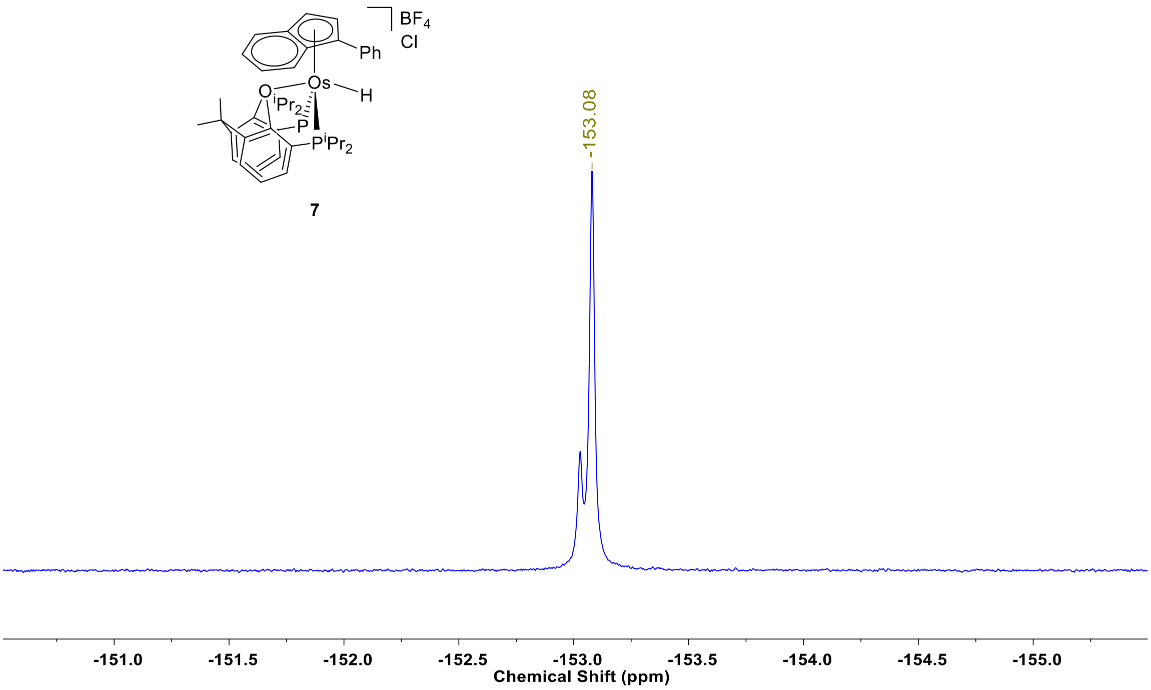

Figure S25. $^{19}F\{^{1}H\}$ NMR spectrum (282.38 MHz, CD_2Cl_2, 298 K) of compound **7**.

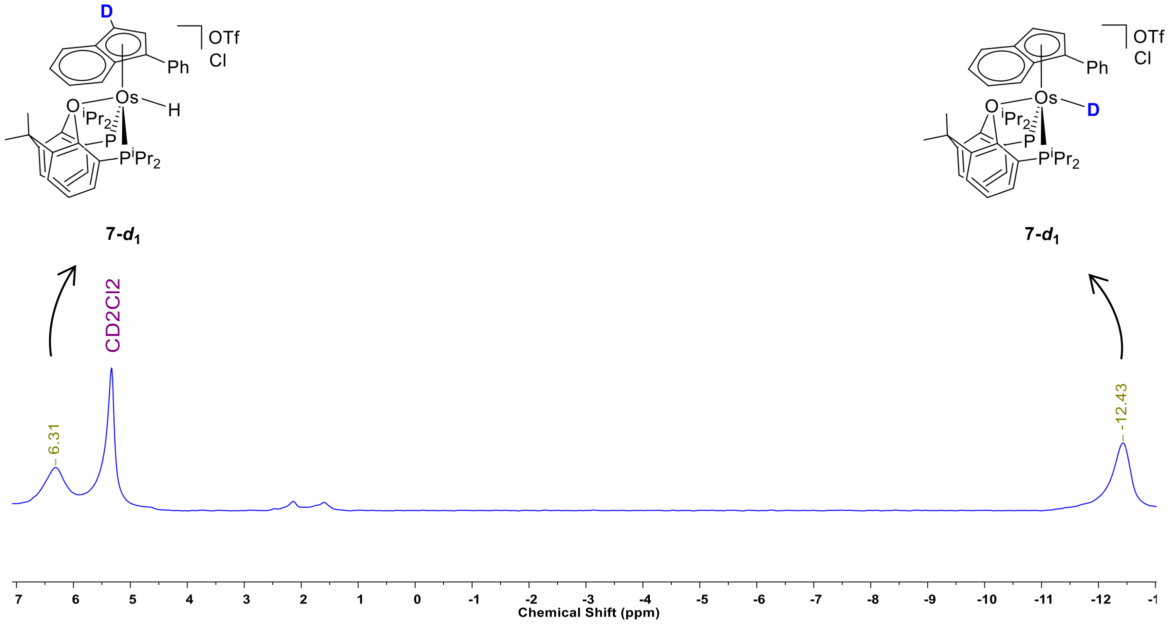

Figure S26. ^{2}H NMR spectrum (61.42 MHz, CH_2Cl_2, 298 K) of **7-*d*₁** isomers.

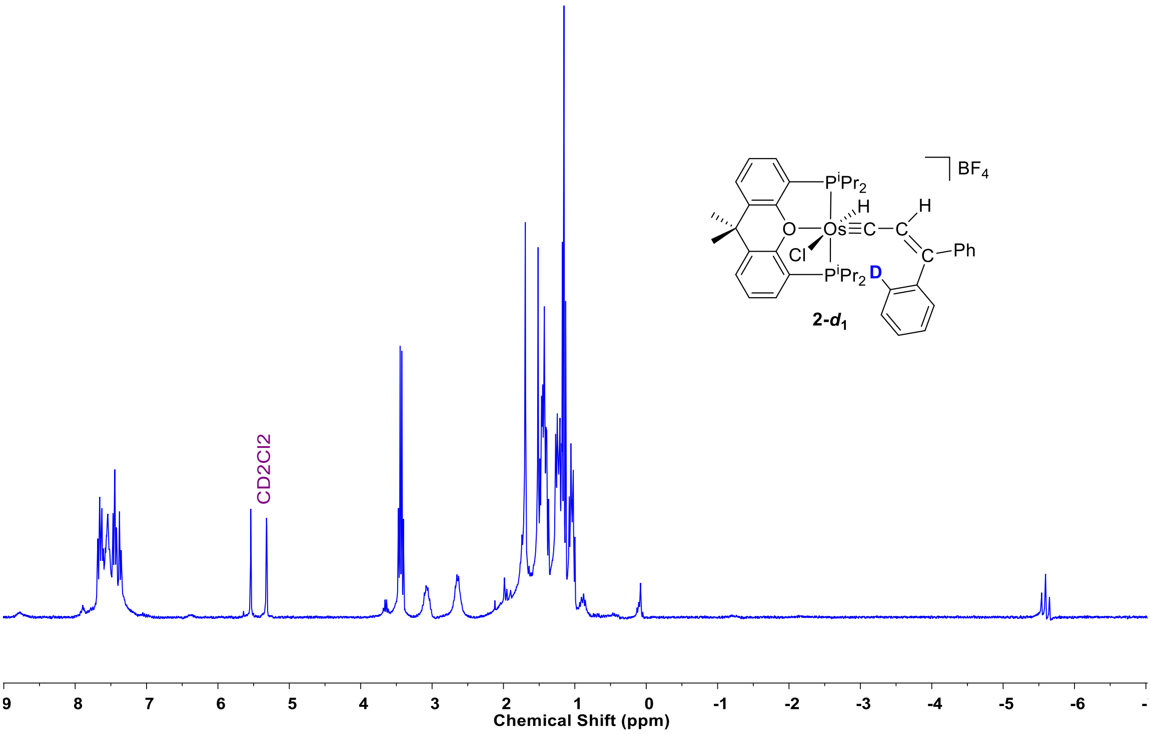

Figure S27. ^{1}H NMR spectrum (300.13 MHz, CD_2Cl_2, 298 K) of the protonation of compound **5** (complex **2-*d*1**).

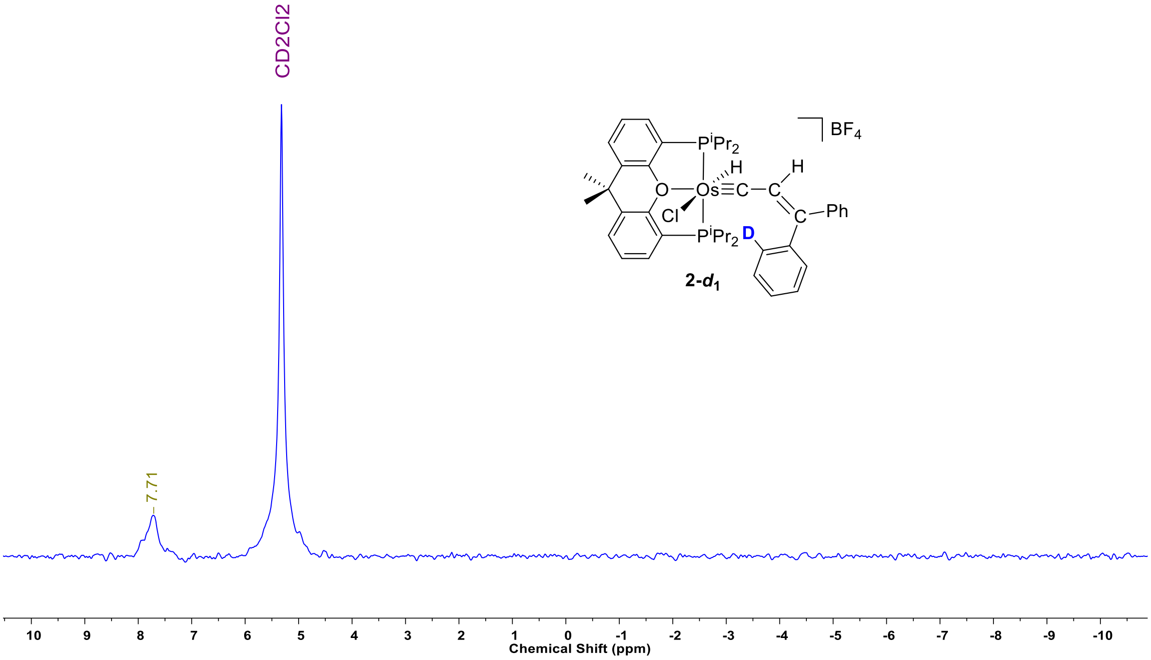

Figure S28. ^{2}H NMR spectrum (61.42 MHz, CH_2Cl_2, 298 K) of the protonation of compound **5** (complex **2-*d*1**).

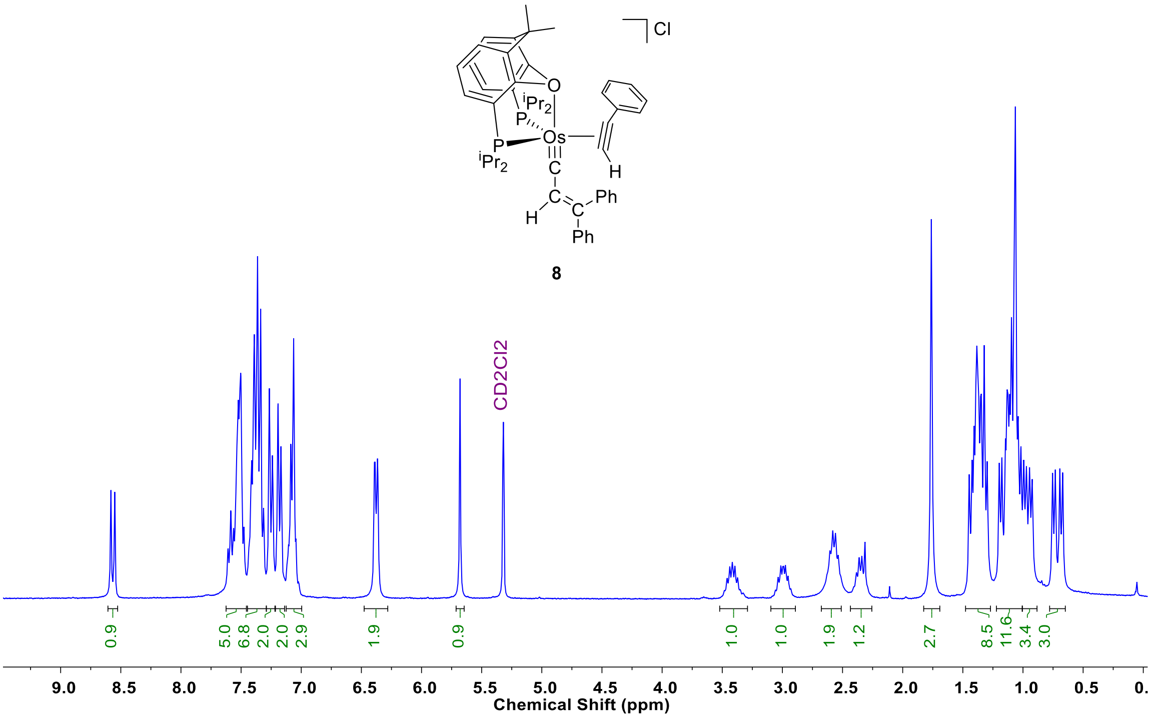

8

Figure S29. ^{1}H NMR spectrum (300.13 MHz, CD_2Cl_2, 253 K) of compound **8**.

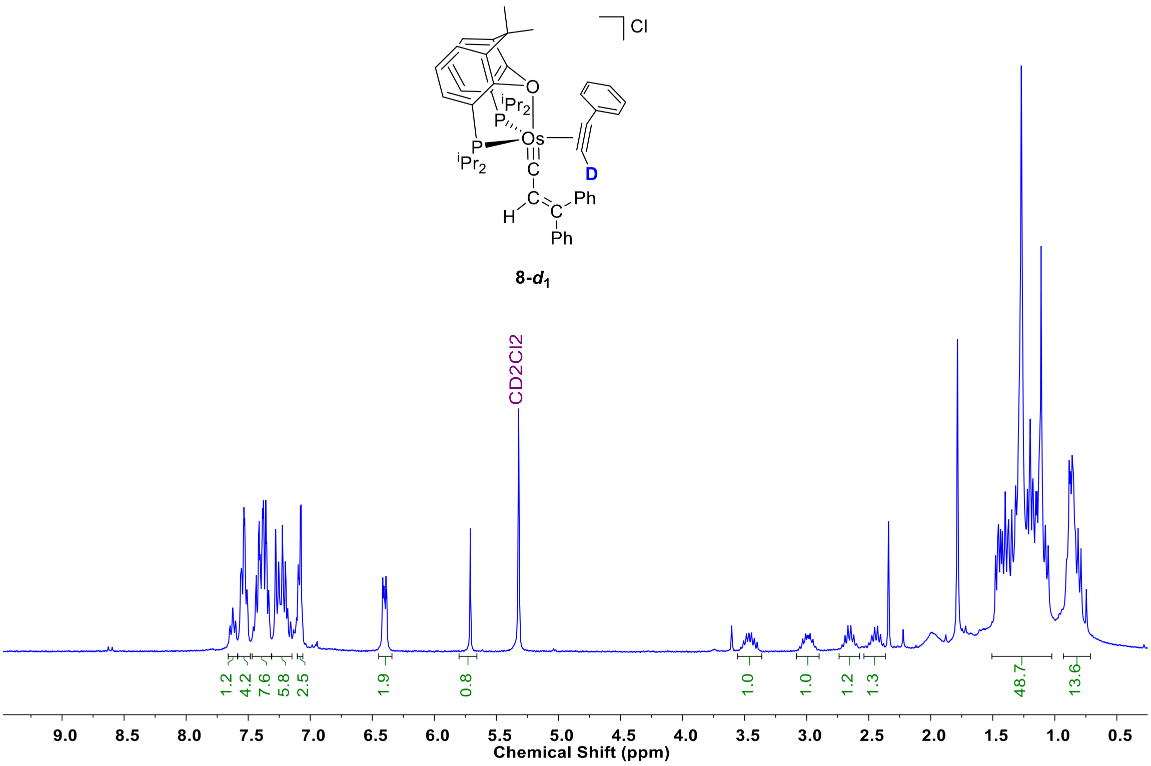

8-*d*$_1$

Figure S30. ^{1}H NMR spectrum (300.13 MHz, CD_2Cl_2, 298 K) of compound **8-*d*$_1$**.

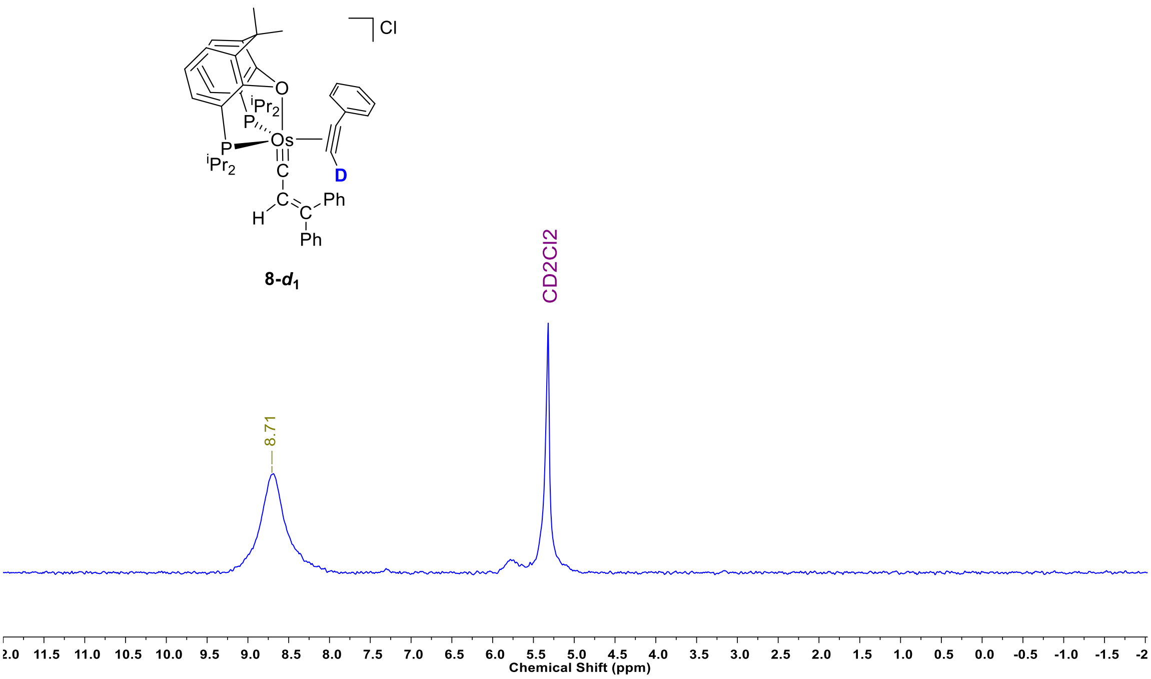

Figure S31. ^{2}H NMR spectrum (61.42 MHz, CH_2Cl_2, 298 K) of compound **8-*d*₁**.

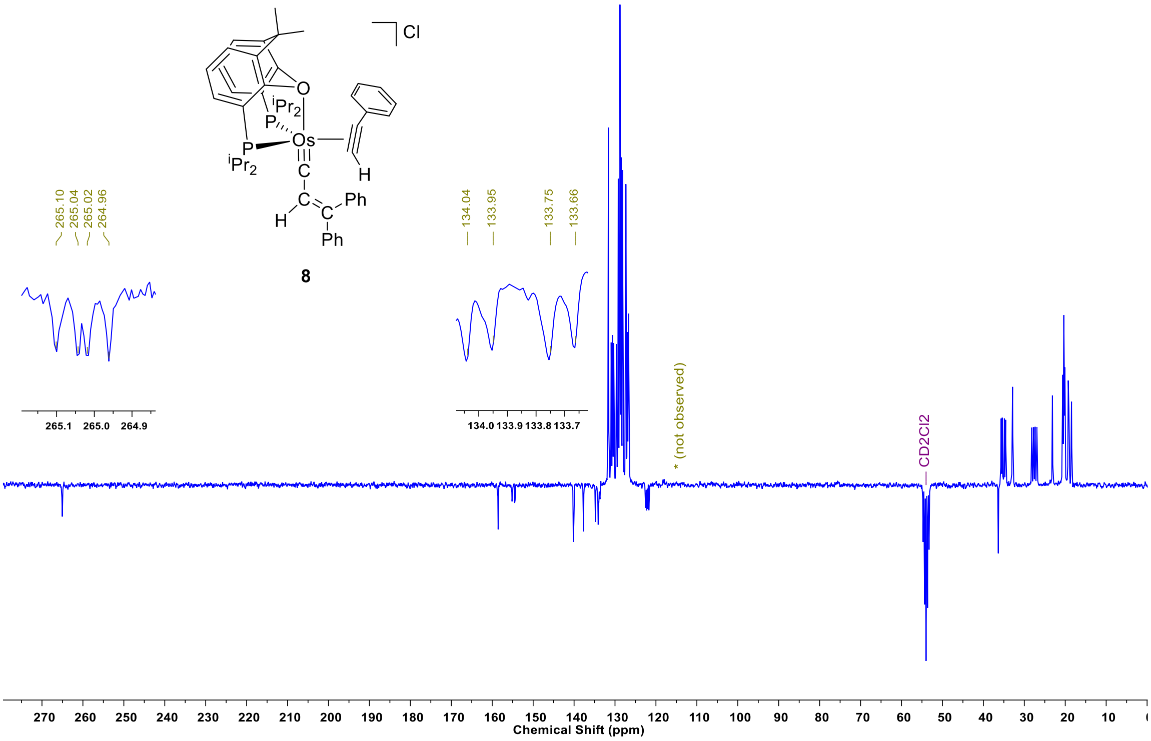

Figure S32. $^{13}C\{^{1}H\}$-APT NMR spectrum (75.48 MHz, CD_2Cl_2, 253 K) of compound **8**.

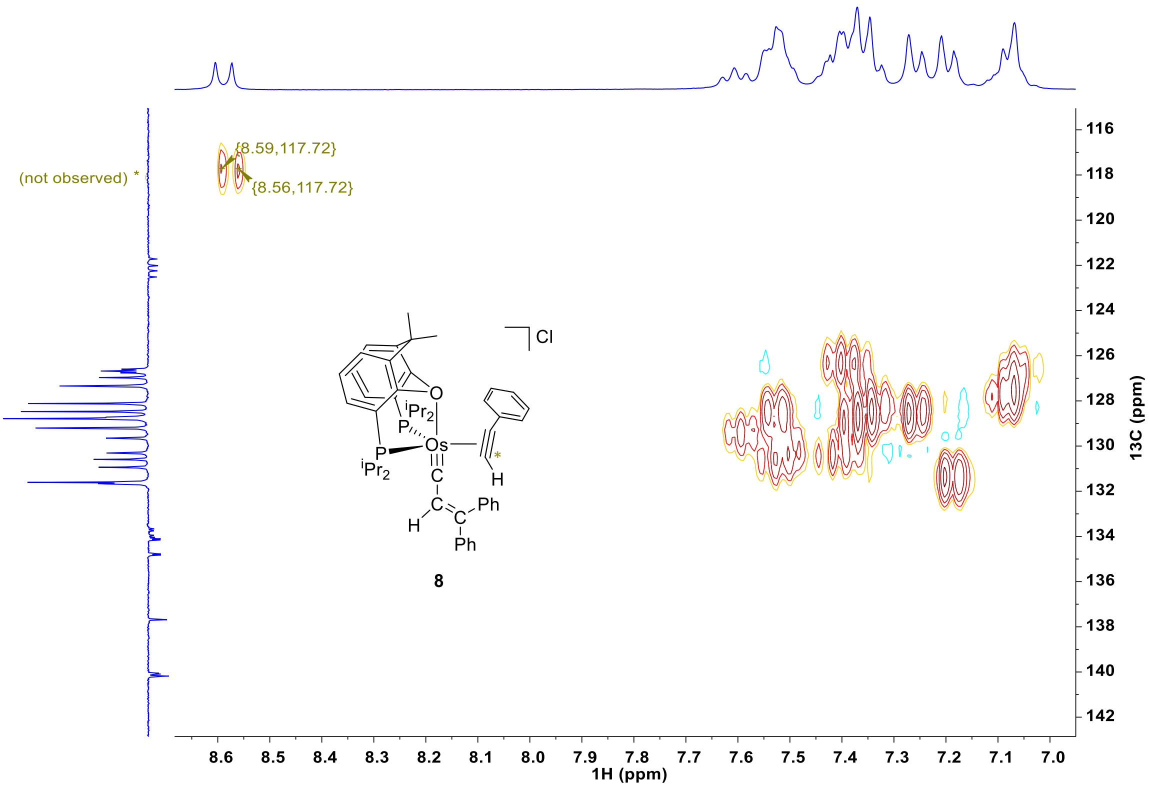

Figure S33. HSQC spectrum (75.48 MHz, CD_2Cl_2, 253 K) of compound **8**.

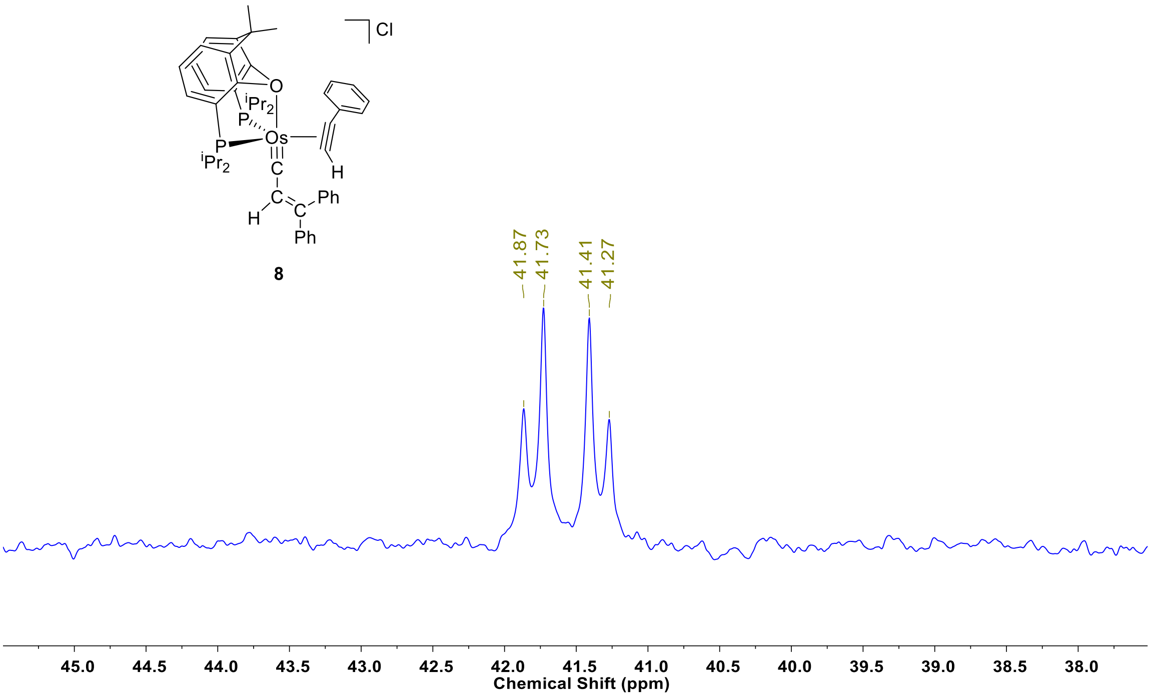

Figure S34. $^{31}P\{^1H\}$ NMR spectrum (121.49 MHz, CD_2Cl_2, 298 K) of compound **8**.

– Computational Details

All calculations in the mechanistic studies were performed at the DFT level using the B3LYP functional[4] supplemented with the Grimme's dispersioncorrection D3[5] including an ultrafine integration grid, as implemented in Gaussian09.[6] Os atom was described by means of an effective core potential SDD for the inner electron[7] and its associated double-ζ basis set for the outer ones, complemented with a set of f-polarization functions.[8] The 6-31G** basis set was used for the H, C, O, and P atoms.[9] All geometries were fully optimized in fluorobenzene (ε = 5.42) solvent using the continuum SMD model.[10] Transition states were identified by having one imaginary frequency in the Hessian matrix. It was confirmed that transition states connect with the corresponding intermediates by means of application of an eigenvector corresponding to the imaginary frequency and subsequent optimization of the resulting structures. Gibbs energies were computed at 298.15 K and 1 atmosphere. All values collected in schemes and figures correspond to Gibbs energies in toluene in kcal mol^{-1}.

To complexes **C** - **4** and **5**, -2.1 kcal/mol were added to compensate for the hydrogen bonds of the 2 explicit water molecules used in the calculations.

The Cartesian coordinates for the computed structures can be found in the supplemental file xyz. The file may be opened as a text file to read the coordinates, or opened directly by a molecular modeling program such as Mercury (version 3.3 or later, http://www.ccdc.cam.ac.uk/pages/Home.aspx) for visualization and analysis.

– DFT computed energy profiles

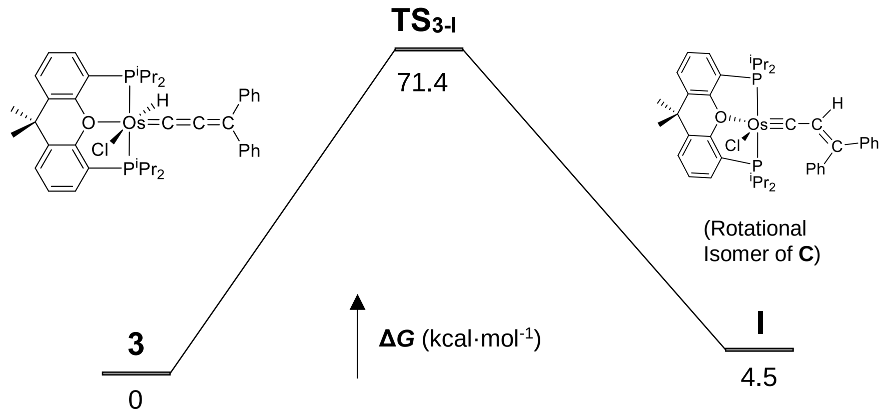

Figure S35. DFT-computed energy profile for complex **3** intramolecular isomerization. Relative free energies (Δ*G* at 298.15 K) are given in kcal·mol^{-1} and were computed at the SMD(fluorobenzene)-B3LYP-D3//SDD(f)/6-31-G** level.

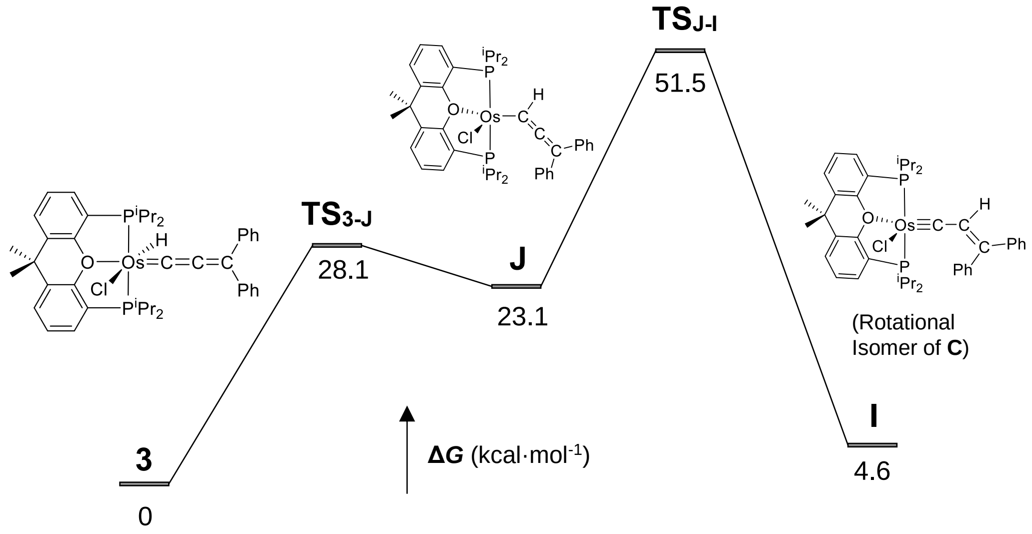

Figure S36. DFT-computed energy profile for complex **3** intramolecular isomerization via the allenyl intermediate **J**. Relative free energies (Δ*G* at 298.15 K) are given in kcal·mol^{-1} and were computed at the SMD(fluorobenzene)-B3LYP-D3//SDD(f)/6-31-G** level

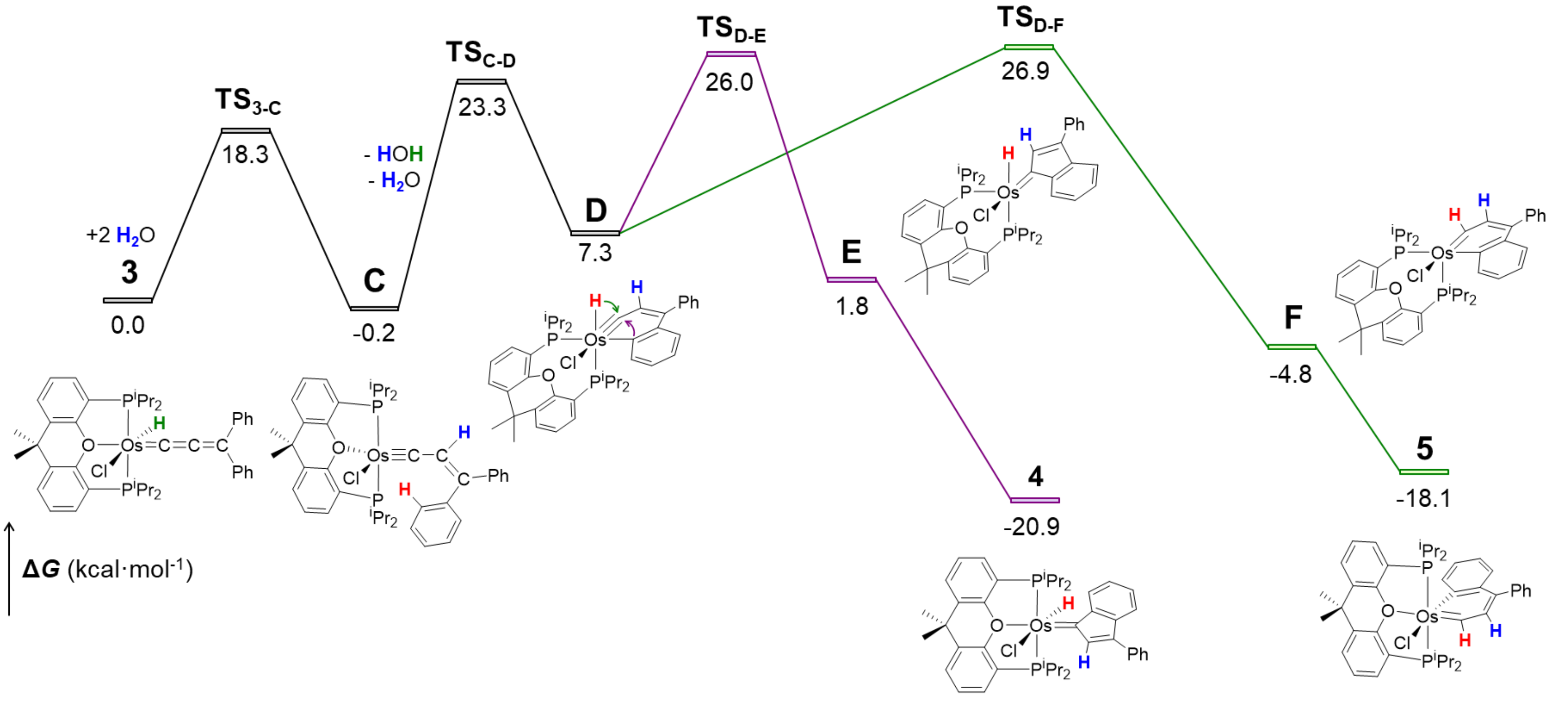

Figure S37. DFT-computed energy profile for complex **3** isomerization. Relative free energies (ΔG at 298.15 K) are given in kcal·mol^{-1} and were computed at the SMD(fluorobenzene)-B3LYP-D3//SDD(f)/6-31-G** level.

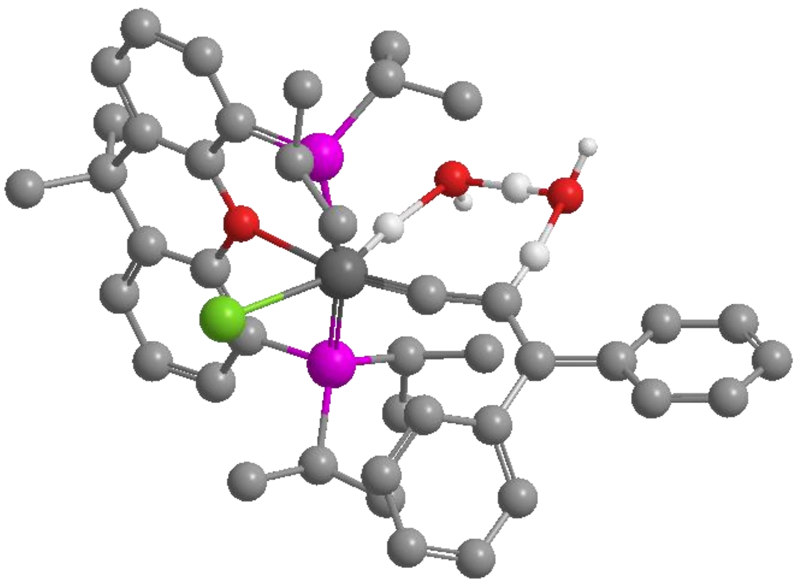

Figure S38. Molecuar diagram of transition state **TS$_{3\text{-C}}$**.

– Energies of Optimized Structures

3:

Zero-point correction= 0.823562 (Hartree/Particle)

Thermal correction to Energy= 0.873261

Thermal correction to Enthalpy= 0.874205

Thermal correction to Gibbs Free Energy= 0.739914

Sum of electronic and zero-point Energies= -2939.581334

Sum of electronic and thermal Energies= -2939.531635

Sum of electronic and thermal Enthalpies= -2939.530691

Sum of electronic and thermal Free Energies= -2939.664982

TS$_{3\text{-I}}$:

Zero-point correction= 0.818119 (Hartree/Particle)

Thermal correction to Energy= 0.868197

Thermal correction to Enthalpy= 0.869141

Thermal correction to Gibbs Free Energy= 0.733724

Sum of electronic and zero-point Energies= -2939.466733

Sum of electronic and thermal Energies= -2939.416655

Sum of electronic and thermal Enthalpies= -2939.415711

Sum of electronic and thermal Free Energies= -2939.551128

I:

Zero-point correction= 0.826067 (Hartree/Particle)

Thermal correction to Energy= 0.875544

Thermal correction to Enthalpy= 0.876488

Thermal correction to Gibbs Free Energy= 0.744351

Sum of electronic and zero-point Energies= -2939.576018

Sum of electronic and thermal Energies= -2939.526541

Sum of electronic and thermal Enthalpies= -2939.525597

Sum of electronic and thermal Free Energies= -2939.657734

TS$_{3\text{-}J}$:

Zero-point correction= 0.821395 (Hartree/Particle)

Thermal correction to Energy= 0.870670

Thermal correction to Enthalpy= 0.871614

Thermal correction to Gibbs Free Energy= 0.739803

Sum of electronic and zero-point Energies= -2939.538613

Sum of electronic and thermal Energies= -2939.489339

Sum of electronic and thermal Enthalpies= -2939.488394

Sum of electronic and thermal Free Energies= -2939.620205

J:

Zero-point correction= 0.824064 (Hartree/Particle)

Thermal correction to Energy= 0.873583

Thermal correction to Enthalpy= 0.874527

Thermal correction to Gibbs Free Energy= 0.742475

Sum of electronic and zero-point Energies= -2939.546611

Sum of electronic and thermal Energies= -2939.497092

Sum of electronic and thermal Enthalpies= -2939.496148

Sum of electronic and thermal Free Energies= -2939.628200

TS_{J-I}:

Zero-point correction= 0.819796 (Hartree/Particle)
Thermal correction to Energy= 0.869304
Thermal correction to Enthalpy= 0.870248
Thermal correction to Gibbs Free Energy= 0.737385
Sum of electronic and zero-point Energies= -2939.500579
Sum of electronic and thermal Energies= -2939.451071
Sum of electronic and thermal Enthalpies= -2939.450127
Sum of electronic and thermal Free Energies= -2939.582990

3+2H_2O:

Zero-point correction= 0.874125 (Hartree/Particle)
Thermal correction to Energy= 0.929011
Thermal correction to Enthalpy= 0.929956
Thermal correction to Gibbs Free Energy= 0.787064
Sum of electronic and zero-point Energies= -3092.422647
Sum of electronic and thermal Energies= -3092.367762
Sum of electronic and thermal Enthalpies= -3092.366817
Sum of electronic and thermal Free Energies= -3092.509709

TS_{3-C}:

Zero-point correction= 0.871437 (Hartree/Particle)
Thermal correction to Energy= 0.924517
Thermal correction to Enthalpy= 0.925461
Thermal correction to Gibbs Free Energy= 0.787904
Sum of electronic and zero-point Energies= -3092.396983
Sum of electronic and thermal Energies= -3092.343903
Sum of electronic and thermal Enthalpies= -3092.342959
Sum of electronic and thermal Free Energies= -3092.480516

C+2H_2O:

Zero-point correction= 0.875550 (Hartree/Particle)
Thermal correction to Energy= 0.930848
Thermal correction to Enthalpy= 0.931793
Thermal correction to Gibbs Free Energy= 0.787397
Sum of electronic and zero-point Energies= -3092.421951
Sum of electronic and thermal Energies= -3092.366653
Sum of electronic and thermal Enthalpies= -3092.365709
Sum of electronic and thermal Free Energies= -3092.510105

C:

Zero-point correction= 0.825924 (Hartree/Particle)
Thermal correction to Energy= 0.875425
Thermal correction to Enthalpy= 0.876369
Thermal correction to Gibbs Free Energy= 0.744089
Sum of electronic and zero-point Energies= -2939.580057
Sum of electronic and thermal Energies= -2939.530557
Sum of electronic and thermal Enthalpies= -2939.529612
Sum of electronic and thermal Free Energies= -2939.661892

TS_{C-D}:

Zero-point correction= 0.820855 (Hartree/Particle)
Thermal correction to Energy= 0.869263
Thermal correction to Enthalpy= 0.870208
Thermal correction to Gibbs Free Energy= 0.742660
Sum of electronic and zero-point Energies= -2939.546370
Sum of electronic and thermal Energies= -2939.497962
Sum of electronic and thermal Enthalpies= -2939.497018
Sum of electronic and thermal Free Energies= -2939.624565

D:

Zero-point correction= 0.824773 (Hartree/Particle)
Thermal correction to Energy= 0.873038
Thermal correction to Enthalpy= 0.873982
Thermal correction to Gibbs Free Energy= 0.747450
Sum of electronic and zero-point Energies= -2939.572738
Sum of electronic and thermal Energies= -2939.524474
Sum of electronic and thermal Enthalpies= -2939.523529
Sum of electronic and thermal Free Energies= -2939.650061

TS$_{D-E}$:

Zero-point correction= 0.823591 (Hartree/Particle)
Thermal correction to Energy= 0.872038
Thermal correction to Enthalpy= 0.872982
Thermal correction to Gibbs Free Energy= 0.744737
Sum of electronic and zero-point Energies= -2939.541359
Sum of electronic and thermal Energies= -2939.492912
Sum of electronic and thermal Enthalpies= -2939.491968
Sum of electronic and thermal Free Energies= -2939.620213

E:

Zero-point correction= 0.825010 (Hartree/Particle)
Thermal correction to Energy= 0.873717
Thermal correction to Enthalpy= 0.874661
Thermal correction to Gibbs Free Energy= 0.745057
Sum of electronic and zero-point Energies= -2939.578778
Sum of electronic and thermal Energies= -2939.530071
Sum of electronic and thermal Enthalpies= -2939.529127
Sum of electronic and thermal Free Energies= -2939.658731

4:

Zero-point correction= 0.825030 (Hartree/Particle)
Thermal correction to Energy= 0.873905
Thermal correction to Enthalpy= 0.874849
Thermal correction to Gibbs Free Energy= 0.743341
Sum of electronic and zero-point Energies= -2939.613196
Sum of electronic and thermal Energies= -2939.564321
Sum of electronic and thermal Enthalpies= -2939.563377
Sum of electronic and thermal Free Energies= -2939.694885

TS$_{D-F}$:

Zero-point correction= 0.822157 (Hartree/Particle)
Thermal correction to Energy= 0.870311
Thermal correction to Enthalpy= 0.871255
Thermal correction to Gibbs Free Energy= 0.744733
Sum of electronic and zero-point Energies= -2939.541276
Sum of electronic and thermal Energies= -2939.493123
Sum of electronic and thermal Enthalpies= -2939.492179
Sum of electronic and thermal Free Energies= -2939.618701

F:

Zero-point correction= 0.826983 (Hartree/Particle)
Thermal correction to Energy= 0.875185
Thermal correction to Enthalpy= 0.876129
Thermal correction to Gibbs Free Energy= 0.749595
Sum of electronic and zero-point Energies= -2939.591939
Sum of electronic and thermal Energies= -2939.543736
Sum of electronic and thermal Enthalpies= -2939.542792
Sum of electronic and thermal Free Energies= -2939.669326

5:

Zero-point correction= 0.826983 (Hartree/Particle)

Thermal correction to Energy= 0.875185

Thermal correction to Enthalpy= 0.876129

Thermal correction to Gibbs Free Energy= 0.749595

Sum of electronic and zero-point Energies= -2939.591939

Sum of electronic and thermal Energies= -2939.543736

Sum of electronic and thermal Enthalpies= -2939.542792

Sum of electronic and thermal Free Energies= -2939.669326

H_2O:

Zero-point correction= 0.021196 (Hartree/Particle)

Thermal correction to Energy= 0.024032

Thermal correction to Enthalpy= 0.024976

Thermal correction to Gibbs Free Energy= 0.003533

Sum of electronic and zero-point Energies= -76.404153

Sum of electronic and thermal Energies= -76.401318

Sum of electronic and thermal Enthalpies= -76.400374

Sum of electronic and thermal Free Energies= -76.421817

– **References**

(1) Esteruelas, M. A.; García-Yebra, C.; Martín, J.; Oñate, E. Mer, Fac, and Bidentate Coordination of an Alkyl-POP Ligand in the Chemistry of Nonclassical Osmium Hydrides. *Inorg. Chem.* **2017**, *56*, 676–683.

(2) Blessing, R. H. *Acta Crystallogr.* **1995**, *A51*, 33. SADABS: Area-detector absorption correction; Bruker- AXS, Madison, WI, 1996.

(3) SHELXL-2016/6. Sheldrick, G. M. *Acta Cryst.* **2008**, *A64*, 112-122.

(4) (a) Lee, C.; Yang, W.; Parr, R. G. Development of the Colle-Salvetti correlation-energy formula into a functional of the electron density. *Phys. Rev. B* **1988**, *37*, 785–789. (b) Becke, A. D. Density-functional thermochemistry .III. The role of exact exchange. *J. Chem. Phys.* **1993**, *98*, 5648–5652. (c) Stephens, P. J.; Devlin, F. J.; Chabalowski, C. F.; Frisch, M. J. Ab Initio Calculation of Vibrational Absorption and Circular Dichroism Spectra Using Density Functional Force Fields *J. Phys. Chem.* **1994**, *98*, 11623–11627.

(5) Grimme, S.; Antony, J.; Ehrlich, S.; Krieg, H. A consistent and accurate ab initio parametrization of density functional dispersion correction (DFT-D) for the 94 elements H-Pu. *J. Chem. Phys.*, **2010**, *132*, 154104-154123.

(6) Gaussian 09, Revision D.01, Frisch, M. J.; Trucks, G. W.; Schlegel H. B.; Scuseria, G. E.; Robb, M. A.; Cheeseman, J. R.; Scalmani, G.; Barone, V.; Mennucci, B.; Petersson, G. A.; Nakatsuji, H.; Caricato, M.; Li, X.; Hratchian, H. P.; Izmaylov, A. F.; Bloino, J.; Zheng, G.; Sonnenberg, J. L.; Hada, M.; Ehara, M.; Toyota, K.; Fukuda, R.; Hasegawa, J.; Ishida, M.; Nakajima, T.; Honda, Y.; Kitao, O.; Nakai, H.; Vreven, T.; Montgomery, J. A.; Peralta, Jr., J. E.; Ogliaro, F.; Bearpark, M.; Heyd, J. J.; Brothers, E.; Kudin, K. N.; Staroverov, V. N.; Keith, T.; Kobayashi, R.; Normand, J.; Raghavachari, K.; Rendell, A.; Burant, J. C.;

Iyengar, S. S.; Tomasi, J.; Cossi, M.; Rega, N.; Millam, J. M.; Klene, M.; Knox, J. E.; Cross, J. B.; Bakken, V.; Adamo, C.; Jaramillo, J.; Gomperts, R.; Stratmann, R. E.; Yazyev, O.; Austin, A. J.; Cammi, R.; Pomelli, C.; Ochterski, J. W.; Martin, R. L.; Morokuma, K.; Zakrzewski, V. G.; Voth, G. A.; Salvador, P.; Dannenberg, J. J.; Dapprich, S.; Daniels, A. D.; Farkas, O.; Foresman, J. B.; Ortiz, J. V.; Cioslowski, J.; Fox, D. J. Gaussian, Inc., Wallingford CT, 2013.

(7) Andrea, D.; Haeussermann, U. M.; Dolg, M.; Stoll, H.; Preuss, H. Energy-adjustedab initio pseudopotentials for the second and third row transition elements *Theor. Chim. Acta* **1990**, *77*, 123–141.

(8) Ehlers, A. W.; Bohme, M.; Dapprich, S.; Gobbi, A.; Hollwarth, A.; Jonas, V.; Kohler, K. F.; Stegmann, R.; Veldkamp, A.; Frenking, G. A set of f-polarization functions for pseudopotential basis sets of the transition metals SC-Cu, Y-Ag and La-Au. *Chem. Phys. Lett.* **1993**, *208*, 111–114.

(9) (a) Hehre, W. J.; Ditchfield, R.; Pople, J. A. Self-Consistent Molecular Orbital Methods. XII. Further Extensions of Gaussian-Type Basis Sets for Use in Molecular Orbital Studies of Organic Molecules. *J. Chem. Phys.* **1972**, *56*, 2257–2261. (b) Francl, M. M.; Pietro, W. J.; Hehre, W. J.; Binkley, J. S.; Gordon, M. S.; DeFrees, D. J.; Pople, J. A. Self_consistent molecular orbital methods. XXIII. A polarization_type basis set for second_row elements. *J. Chem. Phys.* **1982**, *77*, 3654–3665.

(10) Marenich, A. V.; Cramer, C. J.; Truhlar, D. G. Universal Solvation Model Based on Solute Electron Density and on a Continuum Model of the Solvent Defined by the Bulk Dielectric Constant and Atomic Surface Tensions *J. Phys. Chem. B* **2009**, *113*, 6378–6396.
